# #Yourpalaeolife: Interrogating the Status of Fieldwork Among Early Career Palaeontology Researchers

**DOI:** 10.1002/ece3.74032

**Published:** 2026-07-29

**Authors:** Harriet E. Nuttall

**Affiliations:** ^1^ Department of Earth Sciences University of Birmingham Birmingham UK

**Keywords:** early career researcher, extinction of experience, field skills, fieldwork, fossil hunting, geoheritage, palaeontology, research ethics

## Abstract

It has been proposed that palaeontology as an academic discipline is facing an ‘extinction of experience’, whereby junior researchers are not gaining enough fieldwork expertise to be able to perpetuate the practice into the next generation, risking the discipline disconnecting from nature and impacting the acquisition of new data. So far, this idea has been based on anecdotes and without empirical evidence. This study attempts to gather quantitative and qualitative data on the current status of field skills and fieldwork among early career palaeontologists globally by inviting them to complete an online survey. The target group was current PhD candidates and post‐doctoral researchers less than 5 years from finishing their PhD. The results indicate that most field skills training occurs prior to the PhD level and that soft skills (e.g., ethics) are less commonly taught than technical skills (e.g., fossil identification). ECRs generally indicate that they would like more, better advertised, formal skills training opportunities, particularly in palaeontoethics and communication. A lack of professional funding appears to be a more relevant problem in field skills training than in conducting fieldwork. ECRs report a lack of support from their colleagues and institutions and territorial behaviour by other palaeontologists over field sites as primary obstacles to fieldwork engagement. Gender‐based discrimination is common in the sphere of palaeontology fieldwork and lack of inclusivity is a prominent issue. Disputes between senior palaeontologists actively affect the development opportunities accessible to junior researchers. While most ECRs are interested and actively engaging in fieldwork, there are distinct areas of opportunity for the global palaeontological community to engage in cultural change to better support their junior colleagues and reduce barriers to field‐based professional development.

## Graphical abstract

This global survey of early career palaeontologists provides the first quantitative data regarding the prevalence of field skills training and field work among junior researchers of the discipline. The results reveal that ECRs may be underprepared by their education and institutions to undertake fieldwork independently, and that internal research culture is materially obstructing the conducting of field‐based research among this demographic.

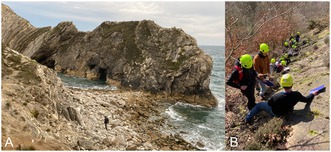

## 1 Introduction

Since the early 1990s, concern has been growing among the scientific community that some part of the fundamental connection that we have with nature may be on the way to weakening (Pyle 1993; Soga and Gaston 2016). The term ‘Extinction of Experience’, coined by Robert Pyle in 1993 to refer to a general segregation of human society and nature, has more recently been purloined by other authors to specify a progressive reduction of time spent conducting fieldwork among the newest generations of natural scientists (Noss 1996; Soga and Gaston 2025; Irwin 2026), with some concerned that a generalised reduction in cumulative experience in the present may perpetuate the decline because future researchers will not be able to learn from the expertise of this generation (Labonté and Trueman 2025; Soga and Gaston 2025). The Mammal Society (2025) in the UK recently published an impassioned open letter calling for greater support for field courses for undergraduate students, citing feedback from the private sector that graduates are no longer equipped with a skillset fit for the ecological field research conducted to understand and protect Britain's ecosystems. Around the same time, Maidment and Butler (2025) raised similar concerns in the discipline of palaeontology, pointing out that acquisition of specimens and data from the field underpins all palaeontological endeavour. Both specified a deficiency in funding for field‐based projects as a key driver of the problems they had described, although neither were able to provide direct quantitative evidence for their observations. Across all natural sciences, there remain very few empirical studies into the status of fieldwork and field skills among modern researchers, the drivers behind an anecdotal decline in fieldwork engagement, or how this may already be impacting our relationship with the natural world (Soga and Gaston 2025).

**FIGURE 1:**
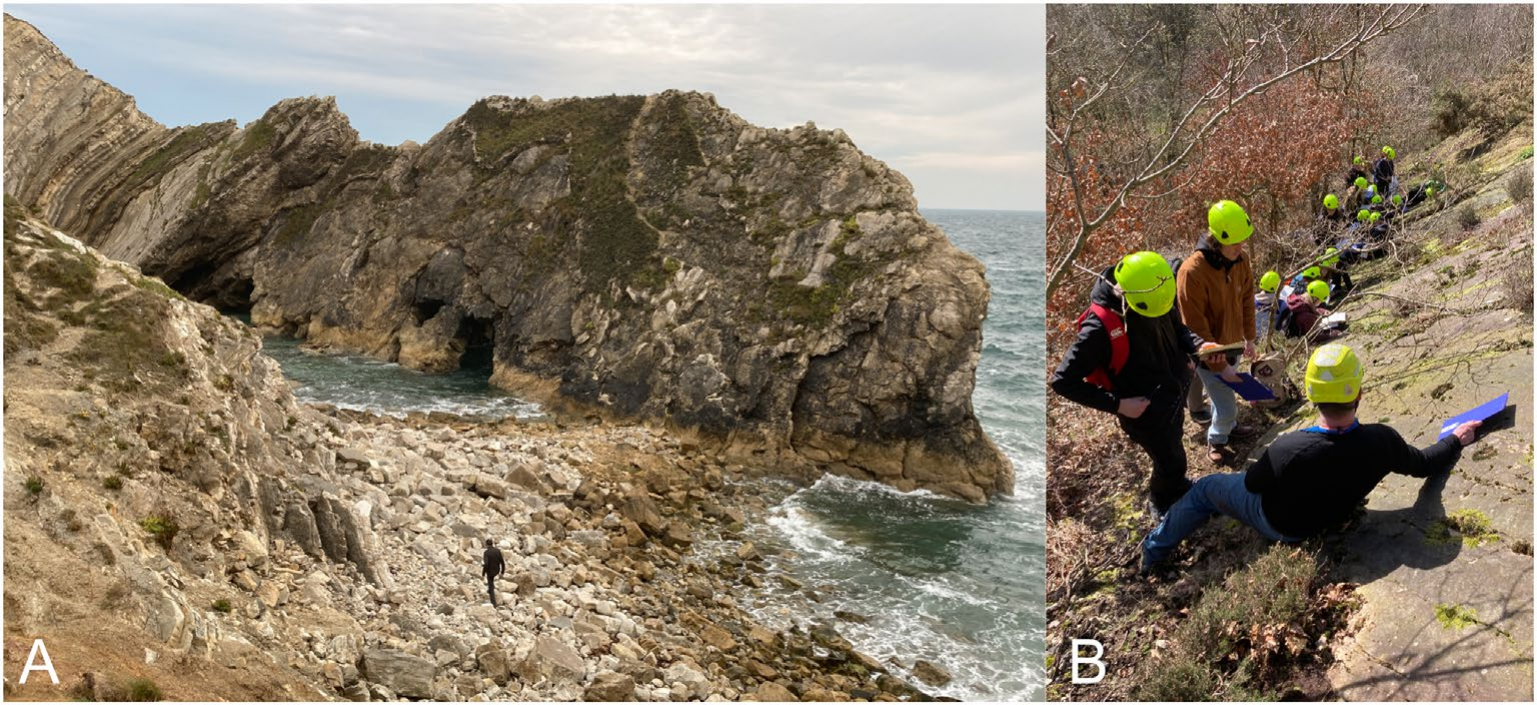
Practical field skills training among ECRs is largely gained before and during their PhD. (A) A PhD researcher prospects for fossils at Stair Hole in Dorset, UK. The spectacular stratigraphy exposed at this site makes it a popular place for UK universities to bring their undergraduates on field trips. (B) Undergraduates from the University of Birmingham receive instruction in field geology at The Wrekin, Shropshire, UK. Images are the author's own.

The present study, the first of a pair of projects intended to cover the breadth of academic palaeontology, is an attempt to reduce this glaring evidentiary shortfall. Here are presented the results of a survey project, in which early career palaeontologists were asked to provide details of their education and experience in the area of fieldwork (Figure 1), and to identify what factors (if any) they had contended with that made it more difficult to acquire associated professional development. From this data, I will outline a robust, empirical state‐of‐play with regards to palaeontology fieldwork among ECRs, with particular focus on the following areas of recent public speculation.

1. Does the early career researcher (ECR) community in palaeontology still have a strong interest in conducting fieldwork?
2. When and how are ECRs receiving training in field skills, and to what degree do they perceive that they have developed competence in applying such skills?
3. What proportion of ECRs are currently engaging in fieldwork and within what professional group structures?
4. Are there any common barriers to obtaining field skills training or fieldwork experienced by the palaeontology ECR community, and do they encounter any discriminatory behaviour during that process (Rafiq et al. 2024)?
5. What are the perceptions and awareness of ECRs with regards to the ethical landscape of scientific fieldwork, including its potential ramifications for the field sites themselves, the traditional stewards of those natural spaces and our collective geoheritage (palaeontoethics: DeMiguel et al. 2021)?

As this is a novel strand of research, it is not possible to put the resultant snapshot into a well‐supported contextual timeline, or demonstrate clear directional trends (such as increasing or decreasing levels of participation); instead, I aim to provide a baseline for future similar assessments such that the status of fieldwork in palaeontology may be subjected to serial health monitoring. I also hope to provoke reflection by individual researchers on their own career path up to this point, and on how they might enact positive change in research practice within their sphere of influence. Moreover, the data are able to distinguish tangible target areas for cultural change within palaeontology that are most likely to improve access to field experience and training for future junior researchers.

## 2 Materials and Methods

The research was conducted using a survey designed within Google Forms (see Supporting Information), and a cascade method of recruitment. Ethics approval was received from the University of Birmingham Science, Technology, Engineering and Mathematics Committee (Application identifier ERN_4269‐Aug2025) on 14 August 2025. Palaeontological researchers and societies were alerted to the project by email to publicly‐available contact addresses, inviting them to participate or distribute the survey to their eligible colleagues and contacts. Additionally, advertisement posts were placed on the author's personal Bluesky social media account, and physical flyers were displayed at the Society of Vertebrate Palaeontology Annual Meeting 2025, in Birmingham, UK. The survey was open for completion between 16 August and 18 November 2025. Written informed consent for data collection, processing and publication was obtained from respondents in the first four questions of the survey. Participation was strictly anonymous and respondent personal information was collected sparingly to prevent incidental identification of ECRs. This key principle was felt to be essential for contributors to feel safe giving full and uncensored responses, without fear of impacting their current and future careers, as well as to protect them from actual negative consequences relating to the information they volunteered. Unfortunately, this requirement meant that geographical information collected could only be extremely coarse‐grain in nature, and as such, it would not be possible to make reliable inferences from location data as part of the research. Primary focus was placed on experiences relating to the most recent 3 years of participants' research activity. Where appropriate, multiple‐selection answers were displayed in a random order to each participant to try to mitigate the effects of response‐order bias by even distribution across the cohort (Krosnik and Alwin 1987).

Eligibility was determined by career stage, with responses sought from self‐described palaeontologists working towards their PhD, or less than 5 years post‐completion of their PhD. The practical definition of an early career researcher is arbitrary, and many do not feel that PhD candidates yet fall within that scope. The Wellcome Trust (2026), for example, specifies completion of the PhD or equivalent research experience in their funding application guidelines. However, the second part of their definition states ‘You may be managing your own research project under the direction of a principal investigator, and will be beginning to develop your own research ideas and directions’; the author felt that this principle applied well enough to those conducting their doctoral studies that their exclusion from the cohort would be unjustified and likely detrimental to the project outcomes.

Data cleaning and the majority of the statistical analysis was performed in Microsoft Excel version 2601 (Microsoft Corporation 2026), and the programme add‐on XLSTAT version 2025.2.0 (Lumivero 2025). Logistic regression models were executed in IBM SPSS Statistics version 30.0.0.0 (IBM 2024).

## 3 Results

### 3.1 Respondent Cohort Characteristics

The survey received 157 responses, with representation of palaeontological research in 44 countries, across six continents (see Supporting Information). An equal number of respondents identified as male and female (43.95%), with 3.82% non‐binary and 8.28% preferring not to give gender information. 56.69% of respondents were current PhD candidates and the remaining 43.31% were researchers less than 5 years post‐PhD. Participant age followed an evident bell‐shaped distribution with the majority of participants between 26 and 35 years of age (69.43%). Age was clearly correlated with career stage (Chi square, *p* < 0.01), with post‐PhD respondents occupying a greater proportion of the upper age brackets. Gender was also significantly associated with career stage (Chi square, *p* = 0.04), with a greater proportion of female and non‐binary palaeontologists at PhD level, and a male majority of post‐PhD researchers. 84.71% of respondents declared they have an active interest in conducting palaeontology fieldwork, 10.19% have previously been interested but no longer are and only 5.10% have never been interested in fieldwork. Interest was not statistically associated with age, gender or career stage.

### 3.2 Pre‐PhD Experience

With regards to field skills, 45.22% of respondents reported receiving training from their undergraduate and/or Masters degree programmes, while 7.64% did not study Palaeontology as an undergraduate or Masters degree. 57.96% experienced fieldwork during their undergraduate degrees, dropping to 43.95% at Masters level. Only one participant (0.64%) declared they had no field skills or fieldwork training pre‐PhD through elective choice, whereas ten respondents (6.37%) reported they had no opportunity for training or fieldwork experience despite studying Palaeontology at undergraduate or Masters level. Several participants described seeking out supplementary experience from alternative sources, such as volunteering with museums.

### 3.3 Training in Specific Field Skills

**FIGURE 2:**
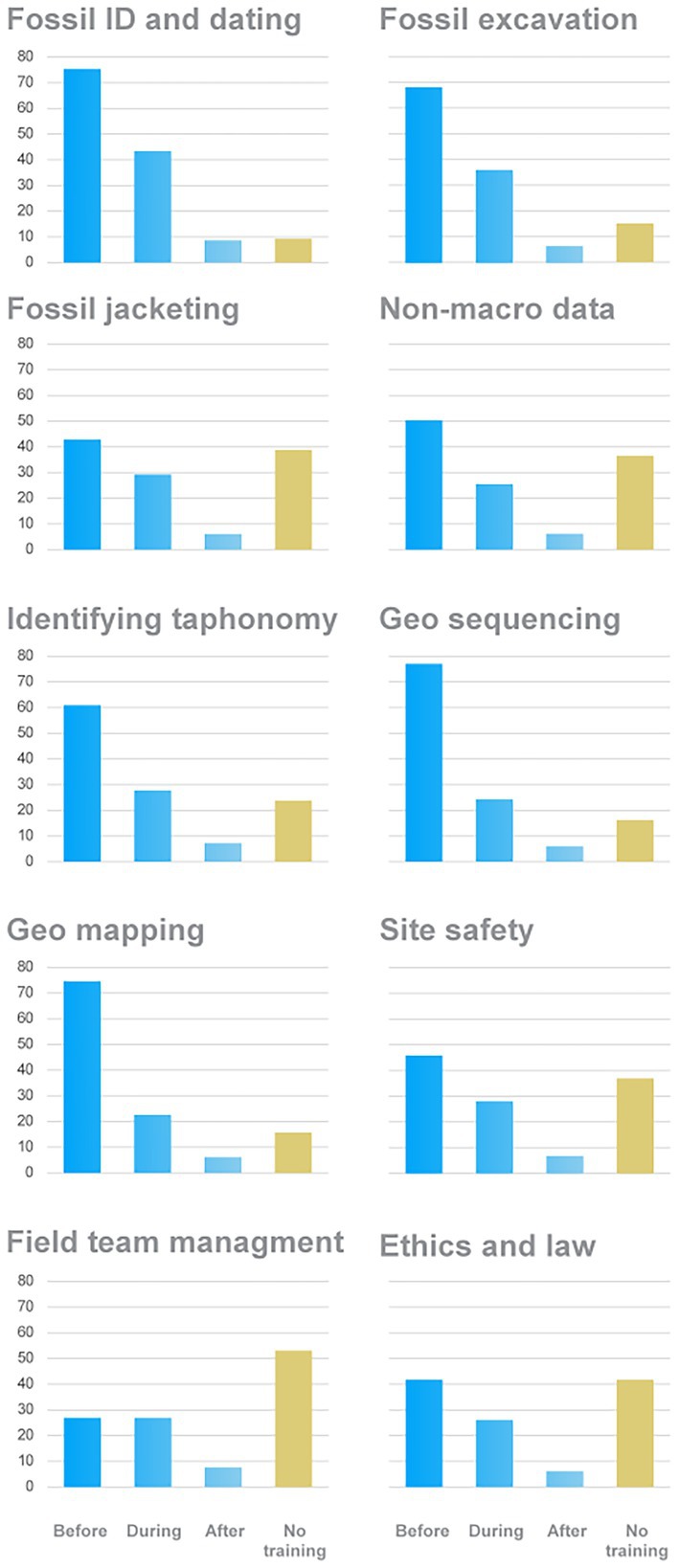
Training reported in individual field skills areas, with timing in relation to the PhD. The figures presented here represent the percentage of all responses to the question, regardless of career stage, but the same trends are replicated when only post‐doctoral researchers are included. See Supporting Information for complete tables.

Participants were asked to detail if and when they had received formal training in each of ten field skills areas (Figure 2). In broad terms, most training was reported to have occurred during the pre‐PhD period, both in the figures for the whole cohort and for the post‐doctoral subset, with training tapering off to much lower levels through the during‐ and after‐PhD phases. In a later open‐answer question regarding suggestions for improvement in training culture, increased emphasis on skills training during the PhD was in the top three proposed areas of action. Training in the ‘soft skills’ areas (safety and site management, field team management and practical application of fieldwork ethics and law) generally was reported less frequently than technical skills, such as fossil excavation and geological sequence interpretation. The current PhD candidates reported significantly higher levels of pre‐PhD skills training than post‐PhD researchers (Wilcoxon signed‐rank test, *p* < 0.01). There appears to be little association between receipt of training and gender, with the only statistical significance occurring with female post‐PhD researchers reporting more training received during their PhDs than males (Wilcoxon signed‐rank test, *p* < 0.05). Without supportive accompanying results in other subsets, this is likely anomalous.

Participants were asked to nominate any skill areas they felt were particularly important to fieldwork, in addition to those listed by the survey. By far the most commonly proposed for training was the ability to communicate sensitively and effectively with local people around field sites (Supporting Information).

### 3.4 Confidence in Specific Field Skills

**FIGURE 3:**
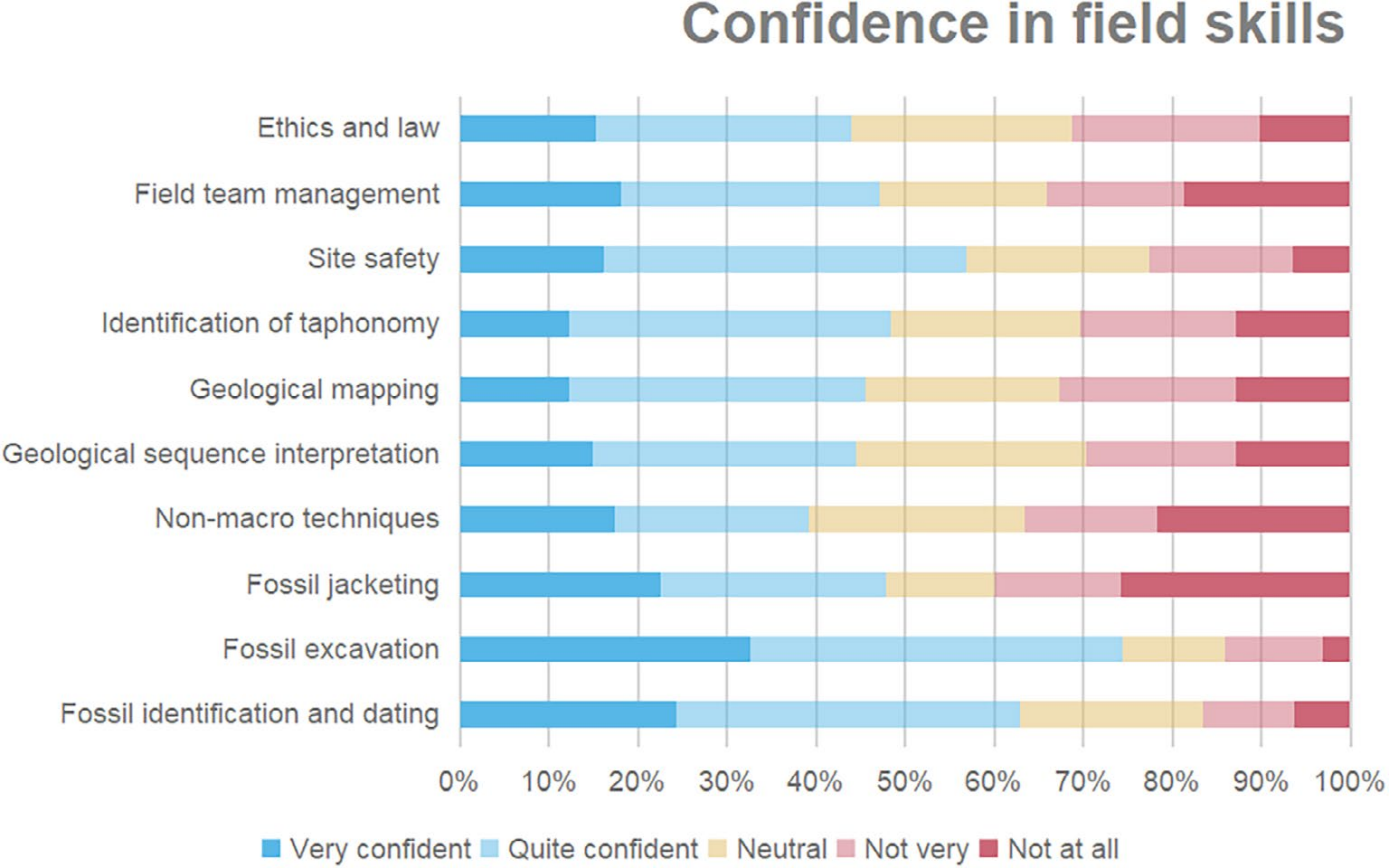
Confidence in field skills among early career researchers. Values presented are the percentage of all responses to the question.

Participants were asked to rate their level of confidence in each of the field skills areas on a five‐part scale from ‘Not at all confident’ to ‘Very confident’ (Figure 3). Of the ten skill categories, only fossil excavation and dating, fossil identification and site safety and management showed more than 50% of respondents reporting themselves ‘Quite confident’ or ‘Very confident’. Fossil excavation showed the highest levels of respondent confidence, with 74.36% ‘Quite confident’ or ‘Very confident’, whereas non‐macrofossil data collection produced the lowest result at 39.09%. Confidence in field skills was not significantly associated with either career stage or gender, while age and confidence displayed a very inconsistent, slightly positive relationship, which was mostly abolished when career stage groups were analysed separately (see Supporting Information). Only field team management maintained a consistently significant association with age across career stage subcategories, with older respondents displaying greater confidence (Spearman's rank correlation values 0.326–0.346, *p* < 0.01). Ordinal linear regression models were run for confidence level versus whether the participant reported receiving training in the particular skill, with gender, age and career stage as additional factors. For all skill areas, a participant reporting no training was significantly more likely to report a lower level of confidence (all *p* < 0.01). Full model outputs can be found in Supporting Information.

### 3.5 Barriers Encountered to Obtaining Field Skills Training

Less than a fifth (19.87%) of the respondent cohort reported they had experienced no barriers at all (Table 1). Financial obstacles featured prominently, with a lack of professional funding and the high cost of training comprising the first and fourth most reported issues (43.59% and 25.64% respectively), while financial aid was proposed several times in the subsequent open‐answer question about training improvements. Also conspicuous were difficulties associated with the ineffectual dissemination of information regarding training opportunities. Poor advertising was the second most frequently selected of the multiple‐choice options (almost a third of responses) and was raised often in open‐answer suggestions for improvement by the palaeontological community. Discrimination (type unspecified) was noted in only 8.97% of responses. Responses facilitated by the ‘other’ category highlighted the COVID‐19 pandemic and lack of direct relevance to current research projects as additional factors curtailing opportunity. Difficulties accessing training were not associated with gender or career stage. Comparison with the age category of respondent did yield significant associations with funding issues (Chi squared, *p* < 0.01), accessibility requirements (*p* = 0.04) and discrimination (*p* < 0.01), although at least the latter two results are not likely reliable as the cohort subcategories were very small. Indeed, further investigation via binary logistic regression modelling was supportive of a positive relationship between age and experience of funding deficiencies only (see Supporting Information).

**TABLE 1:** Barriers to field skills training experienced by ECRs. Percentages are calculated from the total cohort excluding the number of respondents who left the question blank (1).

	Response count	Percentage of responses
Funding	68	43.59
Geographical	38	24.36
No provision by institution	51	32.69
Poor logistical support	39	25.00
Poor support for development	33	21.15
Poor advertising	51	32.69
High cost	40	25.64
Personal circumstances	27	17.31
Need for accessibility adjustments	12	7.69
Low confidence	30	19.23
Travel safety	22	14.10
Don't know what skills	32	20.51
Don't know where to start	38	24.36
Discrimination	14	8.97
Other	11	7.05
No barriers	31	19.87

### 3.6 Fieldwork Participation and Organisation

Overall, fieldwork engagement appears to be prevalent, with nearly 7/10 attending fieldwork organised by their own institution and over half participating via external collaboration (Table 2). In contrast, the majority of researchers at this stage of their career seem to be disengaged from the organisation process of fieldwork trips (Table 3). Solo fieldwork appears to be particularly unpopular, with more than 8/10 reporting they did not attempt to conduct any. Importantly, inability to access fieldwork despite effort to do so appears to be relatively uncommon across the board. When respondents were asked to specify the reasons they had not sought out fieldwork, deficiency of time and irrelevance to current research were most often cited (Supporting Information).

**TABLE 2:** Fieldwork participation among ECRs over the 3 years prior to the survey. Percentages are calculated from the total cohort excluding the number of respondents who left the question blank.

	Successful	Not successful	Not attempted
Within organisation	69.72	10.56	22.54
Outside organisation	52.10	10.92	39.50
Open application	18.39	3.45	79.31

**TABLE 3:** Fieldwork organisation among ECRs over the 3 years prior to the survey. Percentages are calculated from the total cohort excluding the number of respondents who left the question blank.

	Successful	Not successful	Not attempted
Solo fieldwork	16.26	2.44	81.30
With others from own institution	38.24	7.35	55.88
With others outside institution	37.59	3.55	59.57

### 3.7 Barriers to Fieldwork Participation and Organisation

By asking specifically whether they had experienced a refusal to fund their fieldwork, the question regarding obstacles to fieldwork engagement made an attempt to distinguish between actual absence of funding and respondents' perception that funding would not be granted. Perhaps surprisingly, being turned down for funding to participate and organise fieldwork both appeared low on the list of hurdles faced by early career researchers (eighth and thirteenth respectively; Table 4). Open answers to the ‘other’ category included two references to other lack of financial resources. The problem encountered most frequently was a lack of support from ECRs' colleagues and their academic institution, at 22.86% prevalence. Close behind that, at 19.29%, was the situation in which a field site had been unofficially ‘claimed’ by another palaeontologist, and other researchers being consequently warned away from conducting their own investigations there. Official closure of field sites was cited by a small number of respondents via the ‘other’ category. Least commonly reported hurdles were the need for accessibility accommodations at 5.00% and discrimination (type unspecified) at 3.57%. A third of respondents reported experiencing no barriers to fieldwork engagement—decidedly more than for field skills training. Experience of barriers to fieldwork engagement were affected by participant gender, with males experiencing fewer issues than females (Wilcoxon signed‐rank, *p* = 0.04). Researchers who had completed their PhD reported significantly more barriers than those still working towards their doctorate (Wilcoxon signed‐rank, *p* < 0.01). Age was not a significant contributing factor.

**TABLE 4:** Barriers to fieldwork engagement experienced by ECRs. Percentages are calculated from the total cohort excluding the number of respondents who left the question blank (17).

	Response count	Percentage of responses
Declined funding to participate	14	10.00
Declined funding to instigate a trip	18	12.86
Lack of support from colleagues	32	22.86
Concerns regarding ethics of fieldwork	12	8.57
Travel safety	15	10.71
Lack of contacts in target region	20	14.29
Protectionism over fieldwork locations	27	19.29
Protectionism over field trips	16	11.43
Protectionism over data	17	12.14
Administrative burden	23	16.43
Legalities of target area	16	11.43
Personal circumstances	20	14.29
Accessibility requirements	7	5.00
Low confidence	19	13.57
Lack of field skills training	19	13.57
Discrimination	5	3.57

### 3.8 Discrimination Experienced or Perceived by Early Career Researchers

It is important to emphasise that while few respondents appear to have perceived discrimination of any kind as an obstruction to their endeavours in field skills or fieldwork, far more reported actually experiencing or observing some kind of discriminatory behaviour while engaged in these areas: 40.76% with regards to skills training and 33.12% around fieldwork. Prevalence values of specific types of discriminatory attitude are listed in Table 5. With the obvious exception of gender‐based discrimination, which is reported overwhelmingly more frequently by female (37.93%–42.37%) and non‐binary (40%) respondents and extremely rarely by male ones (1.51%–3.23%), protected characteristics are generally uncommon areas of incident. Rather, ECRs identified interpersonal relationships or conflicts between senior research staff, both within and between academic institutions, to be a major source of discriminatory behaviour in their professional lives (Table 5). Across all categories taken together, experience of the various forms of discrimination was not significantly associated with gender or career stage; however, binary logistic regression identified male contributors as much more likely than their female colleagues to select ‘No discrimination’ as their response. This was true in both field skills training (*p* < 0.01, Exp(*B*) = 4.63) and fieldwork (*p* < 0.01, Exp(*B*) = 3.89). Moreover, gender‐based discrimination was more likely to be reported by post‐doctoral researchers than by PhD candidates, with similar statistics in both field skills training (*p* = 0.02, Exp(*B*) = 3.82) and fieldwork (*p* = 0.04, Exp(*B*) = 3.55). No category demonstrated a consistently supported relationship to respondent age.

**TABLE 5:** Discrimination experienced or perceived by ECRs over the 3 years prior to the survey. The ‘prefer not to say’ category comprises both respondents who selected that option and those who chose to give no response to the question.

	Field skills training		Fieldwork engagement	
	Count of responses	Percentage	Count of responses	Percentage
Accessibility requirements	10	6.37	11	7.01
Age	8	5.10	8	5.10
Ethnicity	7	4.46	5	3.18
Gender	29	18.47	28	17.83
Inter‐institutional relationships/conflicts	23	14.65	19	12.10
Intradepartmental relationships/conflicts	25	15.92	19	12.10
Mode of work	6	3.82	6	3.82
Pregnancy and parental leave	0	0.00	0	0.00
Religion	1	0.64	0	0.00
Sexual orientation	3	1.91	1	0.64
Other	7	4.46	3	1.91
Prefer not to say	18	11.46	23	14.65
No discrimination encountered	77	49.04	83	52.87

### 3.9 Do ECRs Have Concerns About the Ethical Context of Fieldwork?

Out of a cohort of 157 researchers, 59 declared some sort of concern regarding the ethical position of conducting palaeontological fieldwork (Supporting Information). The most frequently mentioned considerations were poor collaborative efforts with local researchers, leading to ‘parachute science’ (which appeared 16 times), a disregard for the rights and feelings of the traditional owners of the land on which field sites are found (10 comments), and the vulnerabilities of fossiliferous sites to exploitation and plundering without adequate legal protection (12 comments). A number of respondents also highlighted the potential for abuse of power within field teams, and the enhanced vulnerability of individuals to harassment or social exclusion when travelling to isolated regions with their professional colleagues.

### 3.10 Ethical Malpractice in Palaeontology at Large

A total of 31 respondents chose to anonymously report perceived ethical malpractice by unnamed palaeontology researchers (Supporting Information). Of these, approximately a quarter related to legal infractions around excavation documentation, including two cases of manufacturing fraudulent permits and six of digging outside the legally permitted area. Keeping fossil finds as personal trophies and poor behaviour towards colleagues and employees comprised the next most common areas of disquiet.

### 3.11 Palaeontology's Approach to Fieldwork

In the final open question of the survey, which asked for general commentary surrounding fieldwork and the palaeontological community's approach to it, many of the same themes raised earlier in the form were reiterated (Supporting Information). Most frequently expressed was the desire for improved inclusivity in palaeontology fieldwork culture. Several contributors noted a widespread ‘clique’ mentality in palaeontology, which they had observed perpetuated exclusionary attitudes. Almost as many respondents wished for an expansion of opportunities for field skills training and improved advertising of courses and excursions to early career researchers. Rather than hoping simply for more chances to take up education, many respondents suggested that some training should be emphasised or made mandatory, principally management and planning for fieldwork leaders and ethics and safety principles for all participants. Additionally, some contributors chose to highlight the positive experience they had generally had of fieldwork and collaboration with other researchers outside their own departments.

## 4 Discussion

### 4.1 Prevalence and Timing of Field Skills Training

In many vocational and professional degrees, ‘learning by doing’ via practical and actualised training is a cornerstone of the educational process (e.g., Thompson et al. 2023; Fjeldheim and Kleppe 2025; Baillie et al. 2026; The Geological Society 2026). In contrast, there is little consensus, or even discussion, regarding how to teach early career palaeontologists field skills for maximum opportunity and efficacy. The results of this study clearly indicate that the majority of training occurs pre‐PhD (Figure 2), either within undergraduate and Masters programmes or via voluntary self‐sought experiences. This, of course, makes sense as at this time the focus of budding palaeontologists is firmly on education rather than production of research. Even so, there is clear opportunity for expansion of skills training during this phase, with less than half of respondents reporting receiving any at all at university. Perhaps lessons may be forthcoming from those aforementioned professional and vocational degrees, many of which now increase their skills training capacity by various forms of simulation (e.g., Al‐Elq 2010; Mansell et al. 2024). These workshops can be as simple as role‐play for interpersonal and communication skills (Perez‐Ecija et al. 2025) or as complex as virtual reality scenarios (Wang et al. 2023). Palaeontology regularly engages in simulated activities for outreach, such as children digging in sandpits for buried ‘fossils’, but more sophisticated examples in formal teaching remain rare. Thus, the burden is still placed on short, intensive, real‐world field excursions with minimal classroom support—the responses to the open questions reflected this, with comments such as ‘all of the skills that I have learned originally came from participating in fieldwork’ and ‘cramming all the field techniques into a month or so did not help with retention’ being typical. If we, as palaeontologists, are concerned about the future preparedness of our researchers, perhaps this is an area we can consider evolving.

Moreover, opportunities for catch‐up training at PhD level and after seem to be limited, despite there being an appetite for them among the cohort. Respondents highlighted this: ‘In my experience, field skills training via fieldtrips is rarely offered to PhD students, especially those with no or little previous experience.’ At this career stage, many PhD students are dependent primarily on their PI for mentorship and access to skills teaching. Increasingly, universities and doctoral funding organisations are requiring PhD candidates to formally reflect on their need for training (e.g., Adams and Neary 2022; University of Birmingham 2026) and these documents are generally intended to be shared with the supervisory team. There is joint responsibility, therefore, between the PhD candidate and their PI to identify and arrange access to training—something that is most likely to be successful when there is a strong mentoring relationship (Amador‐Campos et al. 2023). Given the evidence that mentoring can be taught, effectively improving outcomes for both parties (Johnson and Gandhi 2014; Sheri et al. 2019), it seems likely that a general embracing of professional development in mentoring across academic palaeontology would lead to significant cascading benefits in field competency among ECRs.

### 4.2 Confidence Versus Competence

There is an opportunity for error where one is forced, by necessity, to assess confidence as a proxy for competence, namely that asking people to self‐assess renders their answers subject to their own inherent biases (Belotelova and Martin 2025). This study attempts to sensitivity test for this effect by analysing confidence against respondent characteristics, and finds no particularly influential associations. Confidence does appear to be improved by receiving training, as one might anticipate, with the possible (and slightly worrying) exception of site safety, where higher confidence is reported than might be expected from concurrent low levels of instruction. Given reporting in the open questions that leaders of field trips sometimes ignore or under‐prepare for site safety issues, there is a question of whether this is an example of ‘the less you know, the less you know you don't know’ (Kruger and Dunning 1999; Sanchez and Dunning 2018), and more detailed investigation is warranted.

Positive confidence responses sit below half in 7/10 field skills categories—we need to decide, as a community, if we consider this appropriate and sufficient for researchers at this career stage, or whether this represents an under‐achievement that we can collectively act to improve. Simple confidence assessments can be effective in small‐group teaching situations, helping trainers with feedback and fueling reflective, practical teaching improvement (He 2026): these could be leveraged in field skills training contexts. It must also be noted that low confidence, particularly in soft skills involved at the planning stage, may act as a subconscious impediment to ECR engagement with instigating field excursions.

### 4.3 Are Early Career Palaeontologists Doing Enough Fieldwork?

A loaded question. As we have no data to indicate how prevalent fieldwork must be among the palaeontological community to prevent an ‘extinction of experience’ occurring, ‘how much is enough’ is debatable, contentious and likely to be heavily influenced by how much individuals perceive time spent in direct contact with natural fossil sites as important to our identities as researchers. The second #Yourpalaeolife survey, directed at palaeontologists more than 5 years post PhD and run over the winter of 2025–2026, will try to elucidate this issue further. For the present, we could consider ‘enough’ as the equivalent of ‘as much as ECRs would like to be doing’.

When asked why they were not engaging in fieldwork, respondents primarily cited irrelevance to their current research. This survey did not collect information regarding the research directions of participants, but it would seem likely that a proportion are involved to some degree in analytical or quantitative palaeobiology, a digitally‐driven research avenue which utilises data from past discoveries, documentation and museum collections rather than field‐based methods (Dillon et al. 2023; Dean and Thompson 2025). Across the natural sciences, our collective technological and analytical capabilities are continually advancing into new, untrammelled spheres of inquiry (Deshpande et al. 2025). In a diverse and varied academic landscape, fieldwork may not always be a priority to every researcher at every stage of their career. This is perfectly understandable and acceptable, but it is only so if ECRs are not forced into this position by a highly pressurised research culture (Ríos‐Saldaña et al. 2018). Furthermore, one can speculate that the advancement of AI tools in computational biology may swing the balance in either direction (Yu et al. 2024; Silvestro and Pimiento 2025): expanding potential may shift more researchers down analytical routes (Irwin 2026), or the shunting of virtual research onto virtual shoulders could free up resources for ECRs to pursue field‐based projects, boldly going where no AI can go.

### 4.4 Running the Gauntlet of Professional Development

Progression and development are inevitably achieved by the facing down of a series of challenges. It is not in the interests of palaeontology, however, for there to be significant obstructions for ECRs even to access field skills and experiential opportunities in the first place. Barriers to training and field trips are not necessarily the same, and this study did not treat them so, but some common themes did emerge which warrant consideration by the community.

Funding, the perceived reduction or absolute lack of it, is frequently cited in palaeontology as a most important factor in a fieldwork crunch (Rafiq et al. 2024; Labonté and Trueman 2025; Maidment and Butler 2025; H. Nuttall, personal conversations)—but is this actually the case in the evidence? ECRs certainly feel that their personal budgets for training do not readily match up to the costs of improving their field skills. This appears to be exacerbated by a lack of training provision by academic departments (Table 1), which one would imagine would reduce costs to in‐house trainees. The funding environment looks to be less obstructive for fieldwork engagement, at least where applications are actually made (Table 4). External data is difficult to come by when it comes to grant applications made and accepted for fieldwork‐containing projects; queries to several major European bodies, including The Leverhulme Trust, Royal Commission 1851 and Marie Skłodowska‐Curie Actions (MSCA), did not provide any numbers. Freedom of Information requests to UKRI did yield some data (Table 6). This scant information at least implies that planned fieldwork is not necessarily a negative factor in application success. Palaeontology should therefore take care not to create an internal culture whereby we assume a funding application for fieldwork will be unsuccessful and so we decline to make one at all.

**TABLE 6:** Results of a Freedom of Information request made to UKRI, ref. FOI2026/00036 and FOI2026/00273*. This data was as much as could be obtained within FOI budget restrictions, with priority given to years before and after the height of the COVID pandemic. The following questions were posed with regards to applications to the IRF and Doctoral funding schemes: (a) How many palaeontological research applications did they receive in the years 2014–2024, (b) What proportion of those included a fieldwork component and (c) What proportion of funded palaeontology projects included a provision for fieldwork.

Year	No. palaeontological research applications	Proportion including fieldwork proposal	Proportion of successful applications involving fieldwork
2014	27	22%*	40%*
2015	40	33%*	33%*
2016	40	25%	55%
2017	34	38%	33%
2018	48	27%*	0%*
2019	25	—	—
2020	25	—	—
2021	43	—	—
2022	32	38%*	75%*
2023	38	21%	50%
2024	50	20%	0%

If we put aside funding as our favourite boogeyman, even just because it is less within our direct control, in what areas do the results indicate the palaeontological community can make the most efficient positive action? A need for improved information networks is a stand‐out, including more effective publicising of opportunities to ECRs and the creation of open‐access digital resources to better prepare them for planning and conducting fieldwork. Mentorship is important, but peer support can also improve educational and mental health outcomes in fieldwork‐conducting disciplines (Martin and Edwards 1998; Hummel and El Kurd 2021)—it is startling that a lack of enthusiasm from colleagues and institution ranked first out of all impediments to fieldwork engagement in this study (Table 4). Peer support is an internal community issue for palaeontology and questions must be asked as to why this is so.

Perhaps a connected factor is the prevalence of a possessive attitude among researchers with regards to fieldwork locations, termed a form of ‘protectionism’ in this survey, but discussed by ecologists Valdez et al. (2025) as ‘The Gollum Effect’. They demonstrated that territorial behaviour by fellow academics over field sites and data disproportionately impacts early career researchers, to the extent that a proportion change research direction or leave academia entirely. A respondent in this survey described their experience thus: ‘I didn't learn how to approach fieldwork ethically but rather which areas to avoid because someone else is studying them’. Individual palaeontologists can actively reduce this problem by consciously embracing behaviours that are its antithesis: accepting collaboration with ECRs in other institutions, offering advice and support in planning their research trips, making space for external ECRs to join field trips and participate in subsequent publications. In other words, a positive, collaborative, non‐competitive research culture.

Through many parts of the survey, contributors called out for increased inclusivity in fieldwork and in palaeontology in general, describing the current situation with terms such as ‘macho’. Many respondents highlighted a closed‐minded attitude towards accommodating medical needs. Gender‐based discrimination, reported almost exclusively by non‐male respondents, is disappointingly prominent in both the option‐selection and open answer results, and appears to stem from ignorance (‘I think this sort of stuff is often forgotten if people running the trips do not face these issues’) and from active misogyny (‘male older postdocs or professors either looked down on female students or hit on them’). The undervaluing of women scientists is not new; rather, this finding undoubtedly resonates across a raft of academic fields (Boivin et al. 2024), and it is vital that it is not dismissed as some sort of minority concern (Wang et al. 2025). Additionally, some senior researchers seem to be allowing their personal disputes with other palaeontologists to affect their junior researchers' careers (‘I have had no experience of fieldwork with other colleagues … largely due to conflicts within the department’). Overall, it is clear there is a need in palaeontology for widespread community introspection and concomitant cultural change to ensure that, whatever the demographic of the field, there is equal and maximum opportunity for the next generation to develop and grow as researchers (Smith et al. 2025).

### 4.5 Palaeontoethics: Protecting the Relationship Between Researchers and the Earth

Ethics in palaeontology can be discussed (as above) regarding the welfare of the field research teams themselves and with respect to external parties, such as the field sites and their geological and biotic context, the traditional owners and caretakers of the land and the needs of society at large. In recent years, there has been a positive movement in palaeontology to explore the latter as a ‘Big Question’ for the future of the field, particularly with regards to the commercial fossil trade and ‘parachute science’, or the failure of scientists to consult, involve and collaborate with residents and researchers of the areas they excavate (Larson and Russell 2014; DeMiguel et al. 2021; Dunne et al. 2022; Smith et al. 2025). Despite these awareness efforts, the majority of ECRs did not declare any concerns about the ethical considerations of fieldwork generally, and almost 1/5 chose to give details of dubious ethical practices they had observed. A hindrance may be that there is not yet a set of basic ethical principles, widely accepted and fit to be built upon, in palaeontology globally. A number of academic, governmental and independent bodies have proposed general ethical codes of practice which could be adopted or adapted for palaeontological work (e.g., Schroeder et al. 2019; Picot and Grasham 2022; Chartered Institute for Archaeologists 2025; Development Geographies Research Group 2026). Any framework would undoubtedly need to offer an approach of practicality, reasonability, and a willingness to understand the motivations of all parties interested in fossil excavation, with a dominant consideration being the safety and preservation of the field locations themselves.

Particularly evident in this project was a recognition that field sites are vulnerable to exploitation and, in many cases, have little to no enforcement of protection in law. As scientists, we must deeply consider how our research activities may damage or erase the subject of our curiosity—fossils, geological outcrops, the modern counterparts of the ancient species and ecological systems we write our papers about.

This survey demonstrates that there is a broad appetite for expanded teaching of ethics, law and communication among palaeontological ECRs. It would be prudent for academics, therefore, to carefully consider building these topics into their education strategies, so as to create a resilient, responsible palaeontology fit for the challenges of this century.

### 4.6 Limitations of the Methodology and the Study Cohort

It is widely acknowledged that voluntary survey methodology is fundamentally vulnerable to bias, particularly towards the viewpoints of those who feel they have something they wish to say (Cheung et al. 2017; Andrade 2020; Hiratsuka 2025). Non‐response bias may certainly be an issue in this project for several reasons: ECRs may simply not hear about the survey despite efforts to promote it; they may feel (despite specifications to the contrary in the recruitment texts) that if they do not do fieldwork their response will not be useful; they may not feel sufficient incentive to give the project their time. Unfortunately, there were no resources in this case to devote to material incentives, which can help reduce non‐response bias where response rate is low (Singer 2002), and no information regarding the actual present size of the target population. Nevertheless, the total number of respondents was more than adequate for statistical assessment, with an encouraging degree of gender representation and an expected age distribution, so the cohort data appear to be of reasonable quality. Future projects could address the issue via compulsory participation or coordinated random sampling; however, this would be very challenging to implement across such a disparate target group.

The project aimed to provide a broad perspective from the global palaeontological ECR community, and performance here also appears to be quite good, given the number and distribution of countries from which the data was drawn. It should be acknowledged that the USA and UK perspectives are likely over‐represented (see Supporting Information); however, many respondents who mentioned these two countries did also list other places they have worked in the last 3 years, which probably lessens the effect slightly. To an extent, over‐representation by these countries may be a genuine reflection of the large number of palaeontological academic institutions located there compared to other parts of the world (Raja et al. 2022), and the somewhat nomadic lifestyle of an ECR, passing through various countries on short‐term contracts, will often pitch them up in one or the other place at some point in their career. It should, even so, be a qualifier when interpreting the survey results, as should the distinct deficiency of data from South‐East Asia and the Indian subcontinent, a region which did not yield answers to emailed enquiries. Future studies, hopefully with greater resources and more widespread professional contacts, should attempt to generate better data—and minimise biases resulting from the legacy of colonialism (Smith et al. 2025)—by translating materials into multiple languages and partnering with academics in areas that seem to be more difficult to reach by cold emailing (however enthusiastically that was undertaken in this study), whilst at all times being careful to preserve anonymity of respondents.

The present work does not investigate the perspectives and motivations of any other group than early career researchers, and so cannot present information about how these might interplay with those of, most notably, their senior research colleagues. The paired survey ‘#Yourpalaeolife II: the PI perspective’ will efface this deficit in a future publication.

## 5 Conclusions

The ECR community in palaeontology generally appears to be committed to the practice of fieldwork, both in intention and in realised engagement with field trips. Cross‐institutional collaboration is common, although not as frequent as participation within intra‐institutional teams. There is no obvious signal that junior researchers are disconnected from or unenthused by field‐based boots‐on‐the‐ground work, and while this cannot exactly rule out a future ‘Extinction of Experience’, the present outlook is positive. Support and encouragement within institutional and departmental structures are, however, reported to be lacking, and interpersonal behaviours influence professional experiences around fieldwork to an undesirable degree. Opportunities to improve field skills become scarcer as researchers progress to more independent roles: the community demonstrates a desire for this to change, and for learning to continue beyond the higher education phase, particularly with regards to communication and palaeontoethics. The illustration here is not one of external forces or inescapable trends acting directly upon the next generation to stifle or sublimate their field research ambitions; rather, it is vitally important that we, as a discipline, acknowledge that most significant impediments to near‐future field research by ECRs are factors that are within our power to alter, if only we can gather the communal will to do so.

## Author Contributions

**Harriet E. Nuttall:** conceptualization (lead), data curation (lead), formal analysis (lead), investigation (lead), methodology (lead), project administration (lead), resources (lead), software (lead), supervision (lead), validation (lead), visualization (lead), writing – original draft (lead), writing – review and editing (lead).

## Funding

This work was supported by the Natural Environment Research Council (NE/S007350/1).

## Disclosure

Statement on inclusion and positionality: This study was conceived to snapshot the perspectives of early career researchers in palaeontology worldwide. Diverse perspectives are therefore inherent to the study design and the author did her best to contact palaeontologists in as many countries as possible, with reasonable success. The author is resident in the UK: this is likely to have negatively affected recruitment in regions geographically and linguistically distant from that position. There were ethics considerations surrounding the anonymity of participants that restricted the recruitment of international co‐authors in this case, but a larger collective authorship would likely be more effective at reaching participants in some regions, and it is hoped that other academic groups will be inspired by the data presented here. The survey form was drafted only in English, and so speakers of local languages were reliant on browser‐based translation if they were not also English speakers. This is a failing of the methodology, which should be remedied where possible in future research to improve accessibility globally (UNESCO 2021; Steigerwald et al. 2022). The author has only been working in the field of palaeontology for a relatively short time: while this necessarily limits immediate contacts for survey recruitment, it is felt that a less‐internal perspective may have been beneficial to the execution of this work by enhancing objectivity.

## Conflicts of Interest

The author has no known conflicts of interest. Harriet E. Nuttall is a doctoral candidate supported by NERC CENTA2 grant NE/S007350/1.

## Supporting information


**Data S1:** ece374032‐sup‐0001‐Supinfo1.zip.

**Data S2:** ece374032‐sup‐0002‐Supinfo2.zip.

**Data S3:** ece374032‐sup‐0003‐Supinfo3.zip.

## Data Availability

Anonymised survey results are available in the Supporting Information. Some long‐form answers have been removed where consent was not given for their publication. Data is archived in the UBIRA eData repository (https://doi.org/10.25500/edata.bham.00001596), administered by the University of Birmingham.

## References

[ece374032-bib-0001] Adams, E. , and J. Neary . 2022. Strengthening the Role of Training Needs Analysis in Doctoral Training. UKRI. https://www.ukri.org/wp‐content/uploads/2022/06/Strengthening‐the‐role‐of‐TNA‐Report‐April‐2022.pdf.

[ece374032-bib-0002] Al‐Elq, A. H. 2010. “Simulation‐Based Medical Training.” Journal of Family and Community Medicine 17, no. 1: 35–40. 10.4103/1319-1683.68787. 22022669 PMC3195067

[ece374032-bib-0003] Amador‐Campos, J. A. , M. Peró‐Cebollero , M. Feliu‐Torruella , et al. 2023. “Mentoring and Research Self‐Efficacy of Doctoral Students: A Psychometric Approach.” Education in Science 13, no. 4: 358. 10.3390/educsci13040358.

[ece374032-bib-0004] Andrade, C. 2020. “The Limitations of Online Surveys.” Indian Journal of Psychological Medicine 42, no. 6: 575–576. 10.1177/025371762095749. 33354086 PMC7735245

[ece374032-bib-0005] Baillie, S. , N. Booth , A. Catterall , et al. 2026. “A Guide to Veterinary Clinical Skills Laboratories.” https://www.bristol.ac.uk/vetscience/media/docs/csl‐guide.pdf.

[ece374032-bib-0006] Belotelova, A. , and A. K. Martin . 2025. “Confidence Does Not Equal Competence: Socially Dominant Individuals Are More Confident in Their Decisions Without Being More Accurate.” Personality and Individual Differences 236: 113037. 10.1016/j.paid.2024.113037.

[ece374032-bib-0007] Boivin, N. , S. Täuber , U. Beisiegel , U. Keller , and J. G. Hering . 2024. “Sexism in Academia Is Bad for Science and a Waste of Public Funding.” Nature Reviews Materials 9: 1–3. 10.1038/s41578-023-00624-3.

[ece374032-bib-0008] Chartered Institute for Archaeologists . 2025. “Code of Conduct: Professional Ethics in Archaeology.” https://www.archaeologists.net/sites/default/files/2025‐10/Code‐of‐conduct_revOct‐2025.pdf.

[ece374032-bib-0009] Cheung, K. L. , P. M. ten Klooster , C. Smit , H. de Vries , and M. E. Pieterse . 2017. “The Impact of Non‐Response Bias due to Sampling in Public Health Studies: A Comparison of Voluntary Versus Mandatory Recruitment in a Dutch National Survey on Adolescent Health.” BMC Public Health 17, no. 1: 276. 10.1186/s12889-017-4189-8. 28330465 PMC5363011

[ece374032-bib-0010] Dean, C. D. , and J. R. Thompson . 2025. “Museum ‘Dark Data’ Show Variable Impacts on Deep‐Time Biogeographic and Evolutionary History.” Proceedings of the Royal Society B 292, no. 2041: 20242481. 10.1098/rspb.2024.2481. 39999885 PMC11858742

[ece374032-bib-0011] DeMiguel, D. , J. Brilha , L. Alegret , et al. 2021. “Linking Geological Heritage and Geoethics With a Particular Emphasis on Palaeontological Heritage: The New Concept of ‘Palaeontoethics’.” Geoheritage 13: 69. 10.1007/s12371-021-00595-3.

[ece374032-bib-0012] Deshpande, D. , K. Chhugani , T. Ramesh , et al. 2025. “The Evolution of Computational Research in a Data‐Centric World.” Cell 187, no. 17: 4449–4457. 10.1016/j.cell.2024.07.045. PMC11938813 39178828

[ece374032-bib-0013] Development Geographies Research Group . 2026. “DevGRG Ethical Guidelines.” https://developmentgeographiesrg.org/darg‐ethical‐guidelines.

[ece374032-bib-0014] Dillon, E. M. , E. M. Dunne , T. M. Womack , et al. 2023. “Challenges and Directions in Analytical Paleobiology.” Paleobiology 49, no. 3: 377–393. 10.1017/pab.2023.3. 37809321 PMC7615171

[ece374032-bib-0015] Dunne, E. M. , N. B. Raja , P. P. Stewens , Zin‐Maung‐Maung‐Thein , and K. Zaw . 2022. “Ethics, Law, and Politics in Palaeontological Research: The Case of Myanmar Amber.” Communications Biology 5: 1023. 10.1038/s42003-022-03847-2. 36175597 PMC9522859

[ece374032-bib-0016] Fjeldheim, S. , and L. C. Kleppe . 2025. “Facilitating the Integration of Theory and Practice in Skills Training in Social Work Education.” European Journal of Social Work 29, no. 2: 242–256. 10.1080/13691457.2025.2485372.

[ece374032-bib-0017] He, Y. 2026. “I Wasn't Sure Students Were Grasping My Lessons—So I Devised an Experiment.” Science 391, no. 6787: 842.41712734

[ece374032-bib-0018] Hiratsuka, T. 2025. “The Volunteer Participation Paradox: Ethical Tensions Between Self‐Selection and Targeted Sampling.” Research Methods in Applied Linguistics 4, no. 2: 100206. 10.1016/j.rmal.2025.100206.

[ece374032-bib-0019] Hummel, C. , and D. El Kurd . 2021. “Mental Health and Fieldwork.” PS: Political Science & Politics 54, no. 1: 121–125. 10.1017/S1049096520001055.

[ece374032-bib-0057] IBM Corp . 2024. “IBM SPSS Statistics for Windows (Version 30.0.0.0)” [Computer Software]. IBM Corp.

[ece374032-bib-0020] Irwin, A. 2026. “‘I Rarely Get Outside’: Scientists Ditch Fieldwork in the Age of AI.” Nature 649: 278–281. 10.1038/d41586-025-04150-w. 41501204

[ece374032-bib-0021] Johnson, M. O. , and M. Gandhi . 2014. “A Mentor Training Program Improves Mentoring Competency for Researchers Working With Early‐Career Investigators From Underrepresented Backgrounds.” Advances in Health Sciences Education 20: 683–689. 10.1007/s10459-014-9555-z. 25274417 PMC4383738

[ece374032-bib-0022] Krosnik, J. A. , and D. F. Alwin . 1987. “An Evaluation of a Cognitive Theory of Response‐Order Effects in Survey Measurement.” Public Opinion Quarterly 51, no. 2: 201–219. 10.1086/269029.

[ece374032-bib-0023] Kruger, J. , and D. Dunning . 1999. “Unskilled and Unaware of It: How Difficulties in Recognizing One's Own Incompetence Lead to Inflated Self‐Assessments.” Journal of Personality and Social Psychology 77, no. 6: 1121–1134. 10.1037/0022-3514.77.6.1121. 10626367

[ece374032-bib-0024] Labonté, C. , and R. Trueman . 2025. “The Vanishing Field: Why Ecologists Are Spending Less Time Outdoors.” Biodiversity 26, no. 4: 309–311. 10.1080/14888386.2025.2571206.

[ece374032-bib-0025] Larson, P. , and D. Russell . 2014. “The Benefits of Commercial Fossil Sales to 21st Century Paleontology.” Palaeontologia Electronica 17. 10.26879/142.

[ece374032-bib-0056] Lumivero . 2025. “XLSTAT, Version 2025.2.0” [Computer Software]. https://www.xlstat.com/.

[ece374032-bib-0026] Maidment, S. , and R. J. Butler . 2025. “New Frontiers in Dinosaur Exploration.” Biology Letters 21: 20250045. 10.1098/rsbl.2025.0045. 40304201 PMC12042219

[ece374032-bib-0027] Mansell, S. K. , K. Grafton , E. Barnfield , et al. 2024. “Simulation‐Based Education Within Respiratory Physiotherapy Training: A Scoping Review.” ACPRC Journal 56, no. 1: 37–51. 10.56792/KEPM1936.

[ece374032-bib-0028] Martin, M. , and L. Edwards . 1998. “Peer Learning on Fieldwork Placements.” British Journal of Occupational Therapy 61, no. 6: 249–252. 10.1177/030802269806100603.

[ece374032-bib-0055] Microsoft Corporation . 2026. “Microsoft Excel.” https://office.microsoft.com/excel.

[ece374032-bib-0029] Noss, R. F. 1996. “The Naturalists Are Dying Off.” Conservation Biology 10, no. 1: 1–3. 10.1046/j.1523-1739.1996.10010001.x.

[ece374032-bib-0030] Perez‐Ecija, A. , A. Buzon‐Cuevas , A. De Las Heras , and F. J. Mendoza . 2025. “Hierarchically Structured Role‐Playing Simulation as a Tool for Promoting Soft Skills in Veterinary Undergraduates.” Animals 15, no. 11: 1638. 10.3390/ani15111638. 40509104 PMC12153876

[ece374032-bib-0031] Picot, L. , and C. F. Grasham . 2022. Code of Conduct for Ethical Fieldwork. University of Oxford. https://intranet.qeh.ox.ac.uk/sites/default/files/inline‐files/Code%20of%20Conduct%20for%20Ethical%20Fieldwork%20%28June%202022%29%20SoGE.pdf.

[ece374032-bib-0032] Pyle, R. M. 1993. The Thunder Tree: Lessons From an Urban Wildland. Houghton Mifflin.

[ece374032-bib-0033] Rafiq, K. , N. R. Jordan , J. W. McNutt , et al. 2024. “Removing Institutional Barriers to Long‐Term Fieldwork Is Critical for Advancing Ecology.” Trends in Ecology & Evolution 39, no. 12: 1059–1062. 10.1016/j.tree.2024.10.003. 39547845

[ece374032-bib-0034] Raja, N. B. , E. M. Dunne , A. Matiwane , et al. 2022. “Colonial History and Global Economics Distort Our Understanding of Deep‐Time Biodiversity.” Nature Ecology & Evolution 6: 145–154. 10.1038/s41559-021-01608-8. 34969991

[ece374032-bib-0035] Ríos‐Saldaña, C. A. , M. Delibes‐Mateos , and C. C. Ferreira . 2018. “Are Fieldwork Studies Being Relegated to Second Place in Conservation Science?” Global Ecology and Conservation 14: e00389. 10.1016/j.gecco.2018.e00389.

[ece374032-bib-0036] Sanchez, C. , and D. Dunning . 2018. “Overconfidence Among Beginners: Is a Little Learning a Dangerous Thing?” Journal of Personality and Social Psychology 114, no. 1: 10–28. 10.1037/pspa0000102. 29094960

[ece374032-bib-0037] Schroeder, D. , K. Chatfield , M. Singh , R. Chennells , and P. Herissone‐Kelly . 2019. Equitable Research Partnerships: A Global Code of Conduct to Counter Ethics Dumping. Springer.

[ece374032-bib-0038] Sheri, K. , J. Y. J. Too , S. E. L. Chuah , Y. P. Toh , S. Mason , and L. K. Radha Krishna . 2019. “A Scoping Review of Mentor Training Programs in Medicine Between 1990 and 2017.” Medical Education Online 24, no. 1: 1555435. 10.1080/10872981.2018.1555435. 31671284 PMC6327936

[ece374032-bib-0039] Silvestro, D. , and C. Pimiento . 2025. “Emerging Uses of Artificial Intelligence in Deep Time Biodiversity Research.” Nature Reviews Biodiversity 1: 671–677. 10.1038/s44358-025-00075-4.

[ece374032-bib-0040] Singer, E. 2002. “The Use of Incentives to Reduce Nonresponse in Household Surveys.” In Survey Nonresponse, edited by R. M. Groves , D. A. Dillman , J. L. Eltinge , and R. J. A. Little , 163–178. Wiley‐Interscience.

[ece374032-bib-0041] Smith, J. A. , E. M. Dowding , A. A. Abdelhady , et al. 2025. “Identifying the Big Questions in Paleontology: A Community‐Driven Project.” Paleobiology 51, no. 3: 408–431. 10.1017/pab.2025.10042.

[ece374032-bib-0042] Soga, M. , and K. J. Gaston . 2016. “Extinction of Experience: The Loss of Human‐Nature Interactions.” Frontiers in Ecology and the Environment 14, no. 2: 94–101. 10.1002/fee.1225.

[ece374032-bib-0043] Soga, M. , and K. J. Gaston . 2025. “Extinction of Experience Among Ecologists.” Trends in Ecology & Evolution 40, no. 3: 212–215. 10.1016/j.tree.2024.12.010. 39794266

[ece374032-bib-0044] Steigerwald, E. , V. Ramírez‐Castañeda , D. Y. C. Brandt , et al. 2022. “Overcoming Language Barriers in Academia: Machine Translation Tools and a Vision for a Multilingual Future.” Bioscience 72, no. 10: 988–998. 10.1093/biosci/biac062. 36196221 PMC9525128

[ece374032-bib-0045] The Geological Society . 2026. “Chartership.” https://www.geolsoc.org.uk/careers‐and‐training/chartership/.

[ece374032-bib-0046] The Mammal Society . 2025. “Cross‐Sector Concerns About Decline of Fieldwork Opportunities in UK Life Science Courses.” https://mammal.org.uk/press‐hub/cross‐sector‐concerns‐about‐decline‐of‐fieldwork‐opportunities‐in‐uk‐life‐science‐courses.

[ece374032-bib-0047] Thompson, J.‐L. , J. MacKay , and K. B. Blacklock . 2023. “Evaluation of Veterinary Students' Confidence and Competence With Surgical Entrustable Professional Activities After Repeated Use of Low‐Fidelity Training Models.” Veterinary Record 192, no. 8: e2779. 10.1002/vetr.2779. 36912203

[ece374032-bib-0048] UNESCO . 2021. “UNESCO Recommendation on Open Science.” https://unesdoc.unesco.org/ark:/48223/pf0000379949.

[ece374032-bib-0049] University of Birmingham . 2026. “Development Needs Analysis.” https://intranet.birmingham.ac.uk/student/libraries/research/pgr‐development/dna.aspx.

[ece374032-bib-0050] Valdez, J. W. , S. Sharma , and J. Gould . 2025. “Systemic Territoriality in Academia: The Gollum Effect's Impact on Scientific Research and Careers.” One Earth 8, no. 6: 101314. 10.1016/j.oneear.2025.101314.

[ece374032-bib-0051] Wang, H. , J. Sterli , V. Dupret , et al. 2025. “A Decade of Vertebrate Palaeontology Research: Global Taxa Distribution, Gender Dynamics and Evolving Methodologies.” Royal Society Open Science 12, no. 5: 250263. 10.1098/rsos.250263. 40370610 PMC12074809

[ece374032-bib-0052] Wang, L. , F. Zhang , and H. Xie . 2023. “Application of Virtual Simulation in Clinical Skills and Operation Courses.” Frontiers in Medicine 10: 1184392. 10.3389/fmed.2023.1184392. 37305127 PMC10248466

[ece374032-bib-0053] Wellcome Trust . 2026. “Early‐Career Researcher.” https://wellcome.org/research‐funding/guidance/prepare‐to‐apply/early‐career‐research.

[ece374032-bib-0054] Yu, C. , F. Qin , A. Watanabe , et al. 2024. “Artificial Intelligence in Paleontology.” Earth‐Science Reviews 252: 104765. 10.1016/j.earscirev.2024.104765.

