# Supplementary material for: #Yourpalaeolife: Interrogating the Status of Fieldwork Among Early Career Palaeontology Researchers
Source: Ecol Evol. 2026 Jul 29;16(8):e74032. doi: 10.1002/ece3.74032 (PMC13420382; doi:10.1002/ece3.74032)
Supplement: Supplementary file 1 — Data S1: ece374032‐sup‐0001‐Supinfo1.zip. [file ECE3-16-e74032-s003.zip › M1 BLR_BarriersFS_AccexRC.docx]

**Logistic Regression**

| **Notes** |  |  |
| --- | --- | --- |
| Output Created |  | 03-FEB-2026 15:00:46 |
| Comments |  |  |
| Input | Active Dataset | DataSet3 |
|  | Filter | <none> |
|  | Weight | <none> |
|  | Split File | <none> |
|  | N of Rows in Working Data File | 157 |
| Missing Value Handling | Definition of Missing | User-defined missing values are treated as missing |
| Syntax |  | LOGISTIC REGRESSION VARIABLES BTR_Acce /METHOD=ENTER Age_category Career_stage Gender_ID /CONTRAST (Age_category)=Indicator(1) /CONTRAST (Career_stage)=Indicator(1) /CONTRAST (Gender_ID)=Indicator(1) /PRINT=GOODFIT CI(95) /CRITERIA=PIN(0.05) POUT(0.10) ITERATE(20) CUT(0.5). |
| Resources | Processor Time | 00:00:00.02 |
|  | Elapsed Time | 00:00:00.01 |

| **Warnings** |
| --- |
| Text: Age_category Command: LOGISTIC REGRESSION This procedure cannot use string variables longer than 8 bytes. The values will be truncated. |
| Text: Career_stage Command: LOGISTIC REGRESSION This procedure cannot use string variables longer than 8 bytes. The values will be truncated. |

| **Case Processing Summary** |  |  |  |
| --- | --- | --- | --- |
| Unweighted Cases^a^ |  | N | Percent |
| Selected Cases | Included in Analysis | 156 | 99.4 |
|  | Missing Cases | 1 | .6 |
|  | Total | 157 | 100.0 |
| Unselected Cases |  | 0 | .0 |
| Total |  | 157 | 100.0 |

| a. If weight is in effect, see classification table for the total number of cases. |  |  |  |
| --- | --- | --- | --- |

| **Dependent Variable Encoding** |  |
| --- | --- |
| Original Value | Internal Value |
| 0 | 0 |
| 1 | 1 |

| **Categorical Variables Codings** |  |  |  |  |  |  |
| --- | --- | --- | --- | --- | --- | --- |
|  |  | Frequency | Parameter coding |  |  |  |
|  |  |  | (1) | (2) | (3) | (4) |
| Age_category | <25 year | 22 | .000 | .000 | .000 | .000 |
|  | 26-30 ye | 57 | 1.000 | .000 | .000 | .000 |
|  | 31-35 ye | 51 | .000 | 1.000 | .000 | .000 |
|  | 36-40 ye | 18 | .000 | .000 | 1.000 | .000 |
|  | 41+ year | 8 | .000 | .000 | .000 | 1.000 |
| Gender_ID | F | 68 | .000 | .000 | .000 |  |
|  | M | 69 | 1.000 | .000 | .000 |  |
|  | N | 6 | .000 | 1.000 | .000 |  |
|  | U | 13 | .000 | .000 | 1.000 |  |
| Career_stage | PhD cand | 88 | .000 |  |  |  |
|  | Research | 68 | 1.000 |  |  |  |

**Block 0: Beginning Block**

| **Classification Table**^a,b^ |  |  |  |  |  |
| --- | --- | --- | --- | --- | --- |
|  | Observed |  | Predicted |  |  |
|  |  |  | BTR_Acce |  | Percentage Correct |
|  |  |  | 0 | 1 |  |
| Step 0 | BTR_Acce | 0 | 144 | 0 | 100.0 |
|  |  | 1 | 12 | 0 | .0 |
|  | Overall Percentage |  |  |  | 92.3 |

| a. Constant is included in the model. |  |  |  |  |  |
| --- | --- | --- | --- | --- | --- |
| b. The cut value is .500 |  |  |  |  |  |

| **Variables in the Equation** |  |  |  |  |  |  |  |
| --- | --- | --- | --- | --- | --- | --- | --- |
|  |  | B | S.E. | Wald | df | Sig. | Exp(B) |
| Step 0 | Constant | -2.485 | .300 | 68.397 | 1 | <.001 | .083 |

| **Variables not in the Equation** |  |  |  |  |  |
| --- | --- | --- | --- | --- | --- |
|  |  |  | Score | df | Sig. |
| Step 0 | Variables | Age_category | 9.744 | 4 | .045 |
|  |  | Age_category(1) | 7.485 | 1 | .006 |
|  |  | Age_category(2) | 1.770 | 1 | .183 |
|  |  | Age_category(3) | 2.308 | 1 | .129 |
|  |  | Age_category(4) | .703 | 1 | .402 |
|  |  | Career_stage(1) | .217 | 1 | .641 |
|  |  | Gender_ID | 3.377 | 3 | .337 |
|  |  | Gender_ID(1) | 2.653 | 1 | .103 |
|  |  | Gender_ID(2) | .520 | 1 | .471 |
|  |  | Gender_ID(3) | 1.182 | 1 | .277 |
|  | Overall Statistics |  | 12.839 | 8 | .118 |

**Block 1: Method = Enter**

| **Omnibus Tests of Model Coefficients** |  |  |  |  |
| --- | --- | --- | --- | --- |
|  |  | Chi-square | df | Sig. |
| Step 1 | Step | 18.619 | 8 | .017 |
|  | Block | 18.619 | 8 | .017 |
|  | Model | 18.619 | 8 | .017 |

| **Model Summary** |  |  |  |
| --- | --- | --- | --- |
| Step | -2 Log likelihood | Cox & Snell R Square | Nagelkerke R Square |
| 1 | 65.992^a^ | .113 | .269 |

| a. Estimation terminated at iteration number 20 because maximum iterations has been reached. Final solution cannot be found. |  |  |  |
| --- | --- | --- | --- |

| **Hosmer and Lemeshow Test** |  |  |  |
| --- | --- | --- | --- |
| Step | Chi-square | df | Sig. |
| 1 | 2.298 | 8 | .970 |

| **Contingency Table for Hosmer and Lemeshow Test** |  |  |  |  |  |  |
| --- | --- | --- | --- | --- | --- | --- |
|  |  | BTR_Acce = 0 |  | BTR_Acce = 1 |  | Total |
|  |  | Observed | Expected | Observed | Expected |  |
| Step 1 | 1 | 18 | 18.000 | 0 | .000 | 18 |
|  | 2 | 18 | 18.000 | 0 | .000 | 18 |
|  | 3 | 16 | 16.000 | 0 | .000 | 16 |
|  | 4 | 16 | 16.000 | 0 | .000 | 16 |
|  | 5 | 19 | 18.996 | 1 | 1.004 | 20 |
|  | 6 | 11 | 10.812 | 1 | 1.188 | 12 |
|  | 7 | 14 | 14.191 | 2 | 1.809 | 16 |
|  | 8 | 15 | 14.194 | 2 | 2.806 | 17 |
|  | 9 | 10 | 11.806 | 5 | 3.194 | 15 |
|  | 10 | 7 | 5.999 | 1 | 2.001 | 8 |

| **Classification Table**^a^ |  |  |  |  |  |
| --- | --- | --- | --- | --- | --- |
|  | Observed |  | Predicted |  |  |
|  |  |  | BTR_Acce |  | Percentage Correct |
|  |  |  | 0 | 1 |  |
| Step 1 | BTR_Acce | 0 | 144 | 0 | 100.0 |
|  |  | 1 | 12 | 0 | .0 |
|  | Overall Percentage |  |  |  | 92.3 |

| a. The cut value is .500 |  |  |  |  |  |
| --- | --- | --- | --- | --- | --- |

| **Variables in the Equation** |  |  |  |  |  |  |  |
| --- | --- | --- | --- | --- | --- | --- | --- |
|  |  | B | S.E. | Wald | df | Sig. | Exp(B) |
|  |  |  |  |  |  |  |  |
| Step 1^a^ | Age_category |  |  | .105 | 4 | .999 |  |
|  | Age_category(1) | -19.306 | 5098.909 | .000 | 1 | .997 | .000 |
|  | Age_category(2) | -.079 | .931 | .007 | 1 | .933 | .924 |
|  | Age_category(3) | .176 | 1.086 | .026 | 1 | .871 | 1.192 |
|  | Age_category(4) | -19.547 | 14013.419 | .000 | 1 | .999 | .000 |
|  | Career_stage(1) | -.399 | .791 | .254 | 1 | .614 | .671 |
|  | Gender_ID |  |  | 1.879 | 3 | .598 |  |
|  | Gender_ID(1) | .940 | .686 | 1.879 | 1 | .170 | 2.560 |
|  | Gender_ID(2) | -18.055 | 14800.407 | .000 | 1 | .999 | .000 |
|  | Gender_ID(3) | -18.043 | 9925.955 | .000 | 1 | .999 | .000 |
|  | Constant | -2.083 | .704 | 8.749 | 1 | .003 | .124 |

| **Variables in the Equation** |  |  |  |
| --- | --- | --- | --- |
|  |  | 95% C.I.for EXP(B) |  |
|  |  | Lower | Upper |
| Step 1^a^ | Age_category |  |  |
|  | Age_category(1) | .000 | . |
|  | Age_category(2) | .149 | 5.731 |
|  | Age_category(3) | .142 | 10.024 |
|  | Age_category(4) | .000 | . |
|  | Career_stage(1) | .142 | 3.164 |
|  | Gender_ID |  |  |
|  | Gender_ID(1) | .668 | 9.815 |
|  | Gender_ID(2) | .000 | . |
|  | Gender_ID(3) | .000 | . |
|  | Constant |  |  |

|  |  |  |  |  |  |  |  |
| --- | --- | --- | --- | --- | --- | --- | --- |

| a. Variable(s) entered on step 1: Age_category, Career_stage, Gender_ID. |  |  |  |
| --- | --- | --- | --- |
