# Supplementary material for: #Yourpalaeolife: Interrogating the Status of Fieldwork Among Early Career Palaeontology Researchers
Source: Ecol Evol. 2026 Jul 29;16(8):e74032. doi: 10.1002/ece3.74032 (PMC13420382; doi:10.1002/ece3.74032)
Supplement: Supplementary file 1 — Data S1: ece374032‐sup‐0001‐Supinfo1.zip. [file ECE3-16-e74032-s003.zip › M10 BLR_BarriersFS_FundxRC.docx]

**Logistic Regression**

| **Notes** |  |  |
| --- | --- | --- |
| Output Created |  | 03-FEB-2026 14:29:54 |
| Comments |  |  |
| Input | Active Dataset | DataSet3 |
|  | Filter | <none> |
|  | Weight | <none> |
|  | Split File | <none> |
|  | N of Rows in Working Data File | 157 |
| Missing Value Handling | Definition of Missing | User-defined missing values are treated as missing |
| Syntax |  | LOGISTIC REGRESSION VARIABLES BTR_Fund /METHOD=ENTER Age_category Career_stage Gender_ID /CONTRAST (Age_category)=Indicator(1) /CONTRAST (Career_stage)=Indicator(1) /CONTRAST (Gender_ID)=Indicator(1) /PRINT=GOODFIT CI(95) /CRITERIA=PIN(0.05) POUT(0.10) ITERATE(20) CUT(0.5). |
| Resources | Processor Time | 00:00:00.00 |
|  | Elapsed Time | 00:00:00.01 |

| **Warnings** |
| --- |
| Text: Age_category Command: LOGISTIC REGRESSION This procedure cannot use string variables longer than 8 bytes. The values will be truncated. |
| Text: Career_stage Command: LOGISTIC REGRESSION This procedure cannot use string variables longer than 8 bytes. The values will be truncated. |

| **Case Processing Summary** |  |  |  |
| --- | --- | --- | --- |
| Unweighted Cases^a^ |  | N | Percent |
| Selected Cases | Included in Analysis | 156 | 99.4 |
|  | Missing Cases | 1 | .6 |
|  | Total | 157 | 100.0 |
| Unselected Cases |  | 0 | .0 |
| Total |  | 157 | 100.0 |

| a. If weight is in effect, see classification table for the total number of cases. |  |  |  |
| --- | --- | --- | --- |

| **Dependent Variable Encoding** |  |
| --- | --- |
| Original Value | Internal Value |
| 0 | 0 |
| 1 | 1 |

| **Categorical Variables Codings** |  |  |  |  |  |  |
| --- | --- | --- | --- | --- | --- | --- |
|  |  | Frequency | Parameter coding |  |  |  |
|  |  |  | (1) | (2) | (3) | (4) |
| Age_category | <25 year | 22 | .000 | .000 | .000 | .000 |
|  | 26-30 ye | 57 | 1.000 | .000 | .000 | .000 |
|  | 31-35 ye | 51 | .000 | 1.000 | .000 | .000 |
|  | 36-40 ye | 18 | .000 | .000 | 1.000 | .000 |
|  | 41+ year | 8 | .000 | .000 | .000 | 1.000 |
| Gender_ID | F | 68 | .000 | .000 | .000 |  |
|  | M | 69 | 1.000 | .000 | .000 |  |
|  | N | 6 | .000 | 1.000 | .000 |  |
|  | U | 13 | .000 | .000 | 1.000 |  |
| Career_stage | PhD cand | 88 | .000 |  |  |  |
|  | Research | 68 | 1.000 |  |  |  |

**Block 0: Beginning Block**

| **Classification Table**^a,b^ |  |  |  |  |  |
| --- | --- | --- | --- | --- | --- |
|  | Observed |  | Predicted |  |  |
|  |  |  | BTR_Fund |  | Percentage Correct |
|  |  |  | 0 | 1 |  |
| Step 0 | BTR_Fund | 0 | 88 | 0 | 100.0 |
|  |  | 1 | 68 | 0 | .0 |
|  | Overall Percentage |  |  |  | 56.4 |

| a. Constant is included in the model. |  |  |  |  |  |
| --- | --- | --- | --- | --- | --- |
| b. The cut value is .500 |  |  |  |  |  |

| **Variables in the Equation** |  |  |  |  |  |  |  |
| --- | --- | --- | --- | --- | --- | --- | --- |
|  |  | B | S.E. | Wald | df | Sig. | Exp(B) |
| Step 0 | Constant | -.258 | .161 | 2.550 | 1 | .110 | .773 |

| **Variables not in the Equation** |  |  |  |  |  |
| --- | --- | --- | --- | --- | --- |
|  |  |  | Score | df | Sig. |
| Step 0 | Variables | Age_category | 18.231 | 4 | .001 |
|  |  | Age_category(1) | 5.269 | 1 | .022 |
|  |  | Age_category(2) | 5.429 | 1 | .020 |
|  |  | Age_category(3) | 2.540 | 1 | .111 |
|  |  | Age_category(4) | 3.383 | 1 | .066 |
|  |  | Career_stage(1) | 4.287 | 1 | .038 |
|  |  | Gender_ID | 4.935 | 3 | .177 |
|  |  | Gender_ID(1) | .090 | 1 | .764 |
|  |  | Gender_ID(2) | 4.008 | 1 | .045 |
|  |  | Gender_ID(3) | .948 | 1 | .330 |
|  | Overall Statistics |  | 23.985 | 8 | .002 |

**Block 1: Method = Enter**

| **Omnibus Tests of Model Coefficients** |  |  |  |  |
| --- | --- | --- | --- | --- |
|  |  | Chi-square | df | Sig. |
| Step 1 | Step | 25.538 | 8 | .001 |
|  | Block | 25.538 | 8 | .001 |
|  | Model | 25.538 | 8 | .001 |

| **Model Summary** |  |  |  |
| --- | --- | --- | --- |
| Step | -2 Log likelihood | Cox & Snell R Square | Nagelkerke R Square |
| 1 | 188.152^a^ | .151 | .202 |

| a. Estimation terminated at iteration number 4 because parameter estimates changed by less than .001. |  |  |  |
| --- | --- | --- | --- |

| **Hosmer and Lemeshow Test** |  |  |  |
| --- | --- | --- | --- |
| Step | Chi-square | df | Sig. |
| 1 | 2.319 | 7 | .940 |

| **Contingency Table for Hosmer and Lemeshow Test** |  |  |  |  |  |  |
| --- | --- | --- | --- | --- | --- | --- |
|  |  | BTR_Fund = 0 |  | BTR_Fund = 1 |  | Total |
|  |  | Observed | Expected | Observed | Expected |  |
| Step 1 | 1 | 18 | 17.675 | 3 | 3.325 | 21 |
|  | 2 | 16 | 16.210 | 6 | 5.790 | 22 |
|  | 3 | 10 | 11.882 | 7 | 5.118 | 17 |
|  | 4 | 12 | 10.366 | 3 | 4.634 | 15 |
|  | 5 | 10 | 8.987 | 9 | 10.013 | 19 |
|  | 6 | 7 | 7.505 | 10 | 9.495 | 17 |
|  | 7 | 6 | 6.681 | 10 | 9.319 | 16 |
|  | 8 | 6 | 6.019 | 10 | 9.981 | 16 |
|  | 9 | 3 | 2.675 | 10 | 10.325 | 13 |

| **Classification Table**^a^ |  |  |  |  |  |
| --- | --- | --- | --- | --- | --- |
|  | Observed |  | Predicted |  |  |
|  |  |  | BTR_Fund |  | Percentage Correct |
|  |  |  | 0 | 1 |  |
| Step 1 | BTR_Fund | 0 | 59 | 29 | 67.0 |
|  |  | 1 | 20 | 48 | 70.6 |
|  | Overall Percentage |  |  |  | 68.6 |

| a. The cut value is .500 |  |  |  |  |  |
| --- | --- | --- | --- | --- | --- |

| **Variables in the Equation** |  |  |  |  |  |  |  |
| --- | --- | --- | --- | --- | --- | --- | --- |
|  |  | B | S.E. | Wald | df | Sig. | Exp(B) |
|  |  |  |  |  |  |  |  |
| Step 1^a^ | Age_category |  |  | 14.034 | 4 | .007 |  |
|  | Age_category(1) | .784 | .665 | 1.391 | 1 | .238 | 2.191 |
|  | Age_category(2) | 1.862 | .699 | 7.086 | 1 | .008 | 6.435 |
|  | Age_category(3) | 2.094 | .814 | 6.617 | 1 | .010 | 8.116 |
|  | Age_category(4) | 2.726 | 1.035 | 6.936 | 1 | .008 | 15.276 |
|  | Career_stage(1) | .092 | .402 | .052 | 1 | .820 | 1.096 |
|  | Gender_ID |  |  | 4.853 | 3 | .183 |  |
|  | Gender_ID(1) | -.091 | .379 | .058 | 1 | .810 | .913 |
|  | Gender_ID(2) | 2.356 | 1.168 | 4.068 | 1 | .044 | 10.553 |
|  | Gender_ID(3) | -.436 | .689 | .400 | 1 | .527 | .647 |
|  | Constant | -1.627 | .594 | 7.504 | 1 | .006 | .197 |

| **Variables in the Equation** |  |  |  |
| --- | --- | --- | --- |
|  |  | 95% C.I.for EXP(B) |  |
|  |  | Lower | Upper |
| Step 1^a^ | Age_category |  |  |
|  | Age_category(1) | .595 | 8.070 |
|  | Age_category(2) | 1.634 | 25.344 |
|  | Age_category(3) | 1.646 | 40.010 |
|  | Age_category(4) | 2.008 | 116.190 |
|  | Career_stage(1) | .498 | 2.410 |
|  | Gender_ID |  |  |
|  | Gender_ID(1) | .434 | 1.919 |
|  | Gender_ID(2) | 1.069 | 104.188 |
|  | Gender_ID(3) | .167 | 2.497 |
|  | Constant |  |  |

|  |  |  |  |  |  |  |  |
| --- | --- | --- | --- | --- | --- | --- | --- |

| a. Variable(s) entered on step 1: Age_category, Career_stage, Gender_ID. |  |  |  |
| --- | --- | --- | --- |
