# Supplementary material for: #Yourpalaeolife: Interrogating the Status of Fieldwork Among Early Career Palaeontology Researchers
Source: Ecol Evol. 2026 Jul 29;16(8):e74032. doi: 10.1002/ece3.74032 (PMC13420382; doi:10.1002/ece3.74032)
Supplement: Supplementary file 1 — Data S1: ece374032‐sup‐0001‐Supinfo1.zip. [file ECE3-16-e74032-s003.zip › M22 BLR_BarriersFW_ContxRC.docx]

**Logistic Regression**

| **Notes** |  |  |
| --- | --- | --- |
| Output Created |  | 03-FEB-2026 15:32:44 |
| Comments |  |  |
| Input | Active Dataset | DataSet6 |
|  | Filter | <none> |
|  | Weight | <none> |
|  | Split File | <none> |
|  | N of Rows in Working Data File | 157 |
| Missing Value Handling | Definition of Missing | User-defined missing values are treated as missing |
| Syntax |  | LOGISTIC REGRESSION VARIABLES BFW_Cont /METHOD=ENTER Career_stage Age_category Gender_ID /CONTRAST (Career_stage)=Indicator(1) /CONTRAST (Age_category)=Indicator(1) /CONTRAST (Gender_ID)=Indicator(1) /PRINT=GOODFIT CI(95) /CRITERIA=PIN(0.05) POUT(0.10) ITERATE(20) CUT(0.5). |
| Resources | Processor Time | 00:00:00.00 |
|  | Elapsed Time | 00:00:00.01 |

| **Warnings** |
| --- |
| Text: Career_stage Command: LOGISTIC REGRESSION This procedure cannot use string variables longer than 8 bytes. The values will be truncated. |
| Text: Age_category Command: LOGISTIC REGRESSION This procedure cannot use string variables longer than 8 bytes. The values will be truncated. |

| **Case Processing Summary** |  |  |  |
| --- | --- | --- | --- |
| Unweighted Cases^a^ |  | N | Percent |
| Selected Cases | Included in Analysis | 140 | 89.2 |
|  | Missing Cases | 17 | 10.8 |
|  | Total | 157 | 100.0 |
| Unselected Cases |  | 0 | .0 |
| Total |  | 157 | 100.0 |

| a. If weight is in effect, see classification table for the total number of cases. |  |  |  |
| --- | --- | --- | --- |

| **Dependent Variable Encoding** |  |
| --- | --- |
| Original Value | Internal Value |
| 0 | 0 |
| 1 | 1 |

| **Categorical Variables Codings** |  |  |  |  |  |  |
| --- | --- | --- | --- | --- | --- | --- |
|  |  | Frequency | Parameter coding |  |  |  |
|  |  |  | (1) | (2) | (3) | (4) |
| Age_category | <25 year | 18 | .000 | .000 | .000 | .000 |
|  | 26-30 ye | 51 | 1.000 | .000 | .000 | .000 |
|  | 31-35 ye | 46 | .000 | 1.000 | .000 | .000 |
|  | 36-40 ye | 18 | .000 | .000 | 1.000 | .000 |
|  | 41+ year | 7 | .000 | .000 | .000 | 1.000 |
| Gender_ID | F | 60 | .000 | .000 | .000 |  |
|  | M | 63 | 1.000 | .000 | .000 |  |
|  | N | 6 | .000 | 1.000 | .000 |  |
|  | U | 11 | .000 | .000 | 1.000 |  |
| Career_stage | PhD cand | 81 | .000 |  |  |  |
|  | Research | 59 | 1.000 |  |  |  |

**Block 0: Beginning Block**

| **Classification Table**^a,b^ |  |  |  |  |  |
| --- | --- | --- | --- | --- | --- |
|  | Observed |  | Predicted |  |  |
|  |  |  | BFW_Cont |  | Percentage Correct |
|  |  |  | 0 | 1 |  |
| Step 0 | BFW_Cont | 0 | 120 | 0 | 100.0 |
|  |  | 1 | 20 | 0 | .0 |
|  | Overall Percentage |  |  |  | 85.7 |

| a. Constant is included in the model. |  |  |  |  |  |
| --- | --- | --- | --- | --- | --- |
| b. The cut value is .500 |  |  |  |  |  |

| **Variables in the Equation** |  |  |  |  |  |  |  |
| --- | --- | --- | --- | --- | --- | --- | --- |
|  |  | B | S.E. | Wald | df | Sig. | Exp(B) |
| Step 0 | Constant | -1.792 | .242 | 55.035 | 1 | <.001 | .167 |

| **Variables not in the Equation** |  |  |  |  |  |
| --- | --- | --- | --- | --- | --- |
|  |  |  | Score | df | Sig. |
| Step 0 | Variables | Career_stage(1) | .078 | 1 | .780 |
|  |  | Age_category | 6.063 | 4 | .195 |
|  |  | Age_category(1) | 4.627 | 1 | .031 |
|  |  | Age_category(2) | .540 | 1 | .463 |
|  |  | Age_category(3) | .096 | 1 | .757 |
|  |  | Age_category(4) | .000 | 1 | 1.000 |
|  |  | Gender_ID | 7.011 | 3 | .072 |
|  |  | Gender_ID(1) | .943 | 1 | .332 |
|  |  | Gender_ID(2) | 1.857 | 1 | .173 |
|  |  | Gender_ID(3) | 4.752 | 1 | .029 |
|  | Overall Statistics |  | 14.874 | 8 | .062 |

**Block 1: Method = Enter**

| **Omnibus Tests of Model Coefficients** |  |  |  |  |
| --- | --- | --- | --- | --- |
|  |  | Chi-square | df | Sig. |
| Step 1 | Step | 14.497 | 8 | .070 |
|  | Block | 14.497 | 8 | .070 |
|  | Model | 14.497 | 8 | .070 |

| **Model Summary** |  |  |  |
| --- | --- | --- | --- |
| Step | -2 Log likelihood | Cox & Snell R Square | Nagelkerke R Square |
| 1 | 100.335^a^ | .098 | .176 |

| a. Estimation terminated at iteration number 6 because parameter estimates changed by less than .001. |  |  |  |
| --- | --- | --- | --- |

| **Hosmer and Lemeshow Test** |  |  |  |
| --- | --- | --- | --- |
| Step | Chi-square | df | Sig. |
| 1 | 3.023 | 8 | .933 |

| **Contingency Table for Hosmer and Lemeshow Test** |  |  |  |  |  |  |
| --- | --- | --- | --- | --- | --- | --- |
|  |  | BFW_Cont = 0 |  | BFW_Cont = 1 |  | Total |
|  |  | Observed | Expected | Observed | Expected |  |
| Step 1 | 1 | 14 | 13.617 | 0 | .383 | 14 |
|  | 2 | 15 | 15.545 | 1 | .455 | 16 |
|  | 3 | 13 | 12.259 | 0 | .741 | 13 |
|  | 4 | 13 | 12.860 | 1 | 1.140 | 14 |
|  | 5 | 8 | 8.017 | 1 | .983 | 9 |
|  | 6 | 12 | 12.678 | 3 | 2.322 | 15 |
|  | 7 | 12 | 11.748 | 2 | 2.252 | 14 |
|  | 8 | 12 | 12.463 | 3 | 2.537 | 15 |
|  | 9 | 7 | 7.922 | 3 | 2.078 | 10 |
|  | 10 | 14 | 12.891 | 6 | 7.109 | 20 |

| **Classification Table**^a^ |  |  |  |  |  |
| --- | --- | --- | --- | --- | --- |
|  | Observed |  | Predicted |  |  |
|  |  |  | BFW_Cont |  | Percentage Correct |
|  |  |  | 0 | 1 |  |
| Step 1 | BFW_Cont | 0 | 119 | 1 | 99.2 |
|  |  | 1 | 17 | 3 | 15.0 |
|  | Overall Percentage |  |  |  | 87.1 |

| a. The cut value is .500 |  |  |  |  |  |
| --- | --- | --- | --- | --- | --- |

| **Variables in the Equation** |  |  |  |  |  |  |  |
| --- | --- | --- | --- | --- | --- | --- | --- |
|  |  | B | S.E. | Wald | df | Sig. | Exp(B) |
|  |  |  |  |  |  |  |  |
| Step 1^a^ | Career_stage(1) | .749 | .716 | 1.096 | 1 | .295 | 2.115 |
|  | Age_category |  |  | 6.798 | 4 | .147 |  |
|  | Age_category(1) | -2.322 | .896 | 6.714 | 1 | .010 | .098 |
|  | Age_category(2) | -1.197 | .861 | 1.934 | 1 | .164 | .302 |
|  | Age_category(3) | -1.104 | 1.019 | 1.174 | 1 | .279 | .332 |
|  | Age_category(4) | -1.134 | 1.341 | .715 | 1 | .398 | .322 |
|  | Gender_ID |  |  | 8.039 | 3 | .045 |  |
|  | Gender_ID(1) | -.041 | .601 | .005 | 1 | .946 | .960 |
|  | Gender_ID(2) | 1.815 | 1.073 | 2.860 | 1 | .091 | 6.138 |
|  | Gender_ID(3) | 2.099 | .875 | 5.758 | 1 | .016 | 8.155 |
|  | Constant | -1.208 | .597 | 4.100 | 1 | .043 | .299 |

| **Variables in the Equation** |  |  |  |
| --- | --- | --- | --- |
|  |  | 95% C.I.for EXP(B) |  |
|  |  | Lower | Upper |
| Step 1^a^ | Career_stage(1) | .520 | 8.599 |
|  | Age_category |  |  |
|  | Age_category(1) | .017 | .568 |
|  | Age_category(2) | .056 | 1.633 |
|  | Age_category(3) | .045 | 2.442 |
|  | Age_category(4) | .023 | 4.454 |
|  | Gender_ID |  |  |
|  | Gender_ID(1) | .295 | 3.120 |
|  | Gender_ID(2) | .750 | 50.266 |
|  | Gender_ID(3) | 1.469 | 45.276 |
|  | Constant |  |  |

|  |  |  |  |  |  |  |  |
| --- | --- | --- | --- | --- | --- | --- | --- |

| a. Variable(s) entered on step 1: Career_stage, Age_category, Gender_ID. |  |  |  |
| --- | --- | --- | --- |
