# Supplementary material for: #Yourpalaeolife: Interrogating the Status of Fieldwork Among Early Career Palaeontology Researchers
Source: Ecol Evol. 2026 Jul 29;16(8):e74032. doi: 10.1002/ece3.74032 (PMC13420382; doi:10.1002/ece3.74032)
Supplement: Supplementary file 1 — Data S1: ece374032‐sup‐0001‐Supinfo1.zip. [file ECE3-16-e74032-s003.zip › M30 BLR_BarriersFW_LegaxRC.docx]

**Logistic Regression**

| **Notes** |  |  |
| --- | --- | --- |
| Output Created |  | 03-FEB-2026 15:40:10 |
| Comments |  |  |
| Input | Active Dataset | DataSet6 |
|  | Filter | <none> |
|  | Weight | <none> |
|  | Split File | <none> |
|  | N of Rows in Working Data File | 157 |
| Missing Value Handling | Definition of Missing | User-defined missing values are treated as missing |
| Syntax |  | LOGISTIC REGRESSION VARIABLES BFW_Lega /METHOD=ENTER Career_stage Age_category Gender_ID /CONTRAST (Career_stage)=Indicator(1) /CONTRAST (Age_category)=Indicator(1) /CONTRAST (Gender_ID)=Indicator(1) /PRINT=GOODFIT CI(95) /CRITERIA=PIN(0.05) POUT(0.10) ITERATE(20) CUT(0.5). |
| Resources | Processor Time | 00:00:00.00 |
|  | Elapsed Time | 00:00:00.01 |

| **Warnings** |
| --- |
| Text: Career_stage Command: LOGISTIC REGRESSION This procedure cannot use string variables longer than 8 bytes. The values will be truncated. |
| Text: Age_category Command: LOGISTIC REGRESSION This procedure cannot use string variables longer than 8 bytes. The values will be truncated. |

| **Case Processing Summary** |  |  |  |
| --- | --- | --- | --- |
| Unweighted Cases^a^ |  | N | Percent |
| Selected Cases | Included in Analysis | 140 | 89.2 |
|  | Missing Cases | 17 | 10.8 |
|  | Total | 157 | 100.0 |
| Unselected Cases |  | 0 | .0 |
| Total |  | 157 | 100.0 |

| a. If weight is in effect, see classification table for the total number of cases. |  |  |  |
| --- | --- | --- | --- |

| **Dependent Variable Encoding** |  |
| --- | --- |
| Original Value | Internal Value |
| 0 | 0 |
| 1 | 1 |

| **Categorical Variables Codings** |  |  |  |  |  |  |
| --- | --- | --- | --- | --- | --- | --- |
|  |  | Frequency | Parameter coding |  |  |  |
|  |  |  | (1) | (2) | (3) | (4) |
| Age_category | <25 year | 18 | .000 | .000 | .000 | .000 |
|  | 26-30 ye | 51 | 1.000 | .000 | .000 | .000 |
|  | 31-35 ye | 46 | .000 | 1.000 | .000 | .000 |
|  | 36-40 ye | 18 | .000 | .000 | 1.000 | .000 |
|  | 41+ year | 7 | .000 | .000 | .000 | 1.000 |
| Gender_ID | F | 60 | .000 | .000 | .000 |  |
|  | M | 63 | 1.000 | .000 | .000 |  |
|  | N | 6 | .000 | 1.000 | .000 |  |
|  | U | 11 | .000 | .000 | 1.000 |  |
| Career_stage | PhD cand | 81 | .000 |  |  |  |
|  | Research | 59 | 1.000 |  |  |  |

**Block 0: Beginning Block**

| **Classification Table**^a,b^ |  |  |  |  |  |
| --- | --- | --- | --- | --- | --- |
|  | Observed |  | Predicted |  |  |
|  |  |  | BFW_Lega |  | Percentage Correct |
|  |  |  | 0 | 1 |  |
| Step 0 | BFW_Lega | 0 | 124 | 0 | 100.0 |
|  |  | 1 | 16 | 0 | .0 |
|  | Overall Percentage |  |  |  | 88.6 |

| a. Constant is included in the model. |  |  |  |  |  |
| --- | --- | --- | --- | --- | --- |
| b. The cut value is .500 |  |  |  |  |  |

| **Variables in the Equation** |  |  |  |  |  |  |  |
| --- | --- | --- | --- | --- | --- | --- | --- |
|  |  | B | S.E. | Wald | df | Sig. | Exp(B) |
| Step 0 | Constant | -2.048 | .266 | 59.421 | 1 | <.001 | .129 |

| **Variables not in the Equation** |  |  |  |  |  |
| --- | --- | --- | --- | --- | --- |
|  |  |  | Score | df | Sig. |
| Step 0 | Variables | Career_stage(1) | .160 | 1 | .689 |
|  |  | Age_category | 4.939 | 4 | .294 |
|  |  | Age_category(1) | 1.019 | 1 | .313 |
|  |  | Age_category(2) | 4.481 | 1 | .034 |
|  |  | Age_category(3) | .704 | 1 | .401 |
|  |  | Age_category(4) | .059 | 1 | .807 |
|  |  | Gender_ID | 11.915 | 3 | .008 |
|  |  | Gender_ID(1) | 7.709 | 1 | .005 |
|  |  | Gender_ID(2) | .170 | 1 | .680 |
|  |  | Gender_ID(3) | 7.333 | 1 | .007 |
|  | Overall Statistics |  | 17.309 | 8 | .027 |

**Block 1: Method = Enter**

| **Omnibus Tests of Model Coefficients** |  |  |  |  |
| --- | --- | --- | --- | --- |
|  |  | Chi-square | df | Sig. |
| Step 1 | Step | 16.906 | 8 | .031 |
|  | Block | 16.906 | 8 | .031 |
|  | Model | 16.906 | 8 | .031 |

| **Model Summary** |  |  |  |
| --- | --- | --- | --- |
| Step | -2 Log likelihood | Cox & Snell R Square | Nagelkerke R Square |
| 1 | 82.601^a^ | .114 | .224 |

| a. Estimation terminated at iteration number 6 because parameter estimates changed by less than .001. |  |  |  |
| --- | --- | --- | --- |

| **Hosmer and Lemeshow Test** |  |  |  |
| --- | --- | --- | --- |
| Step | Chi-square | df | Sig. |
| 1 | 6.910 | 7 | .438 |

| **Contingency Table for Hosmer and Lemeshow Test** |  |  |  |  |  |  |
| --- | --- | --- | --- | --- | --- | --- |
|  |  | BFW_Lega = 0 |  | BFW_Lega = 1 |  | Total |
|  |  | Observed | Expected | Observed | Expected |  |
| Step 1 | 1 | 12 | 12.824 | 1 | .176 | 13 |
|  | 2 | 9 | 8.859 | 0 | .141 | 9 |
|  | 3 | 14 | 13.748 | 0 | .252 | 14 |
|  | 4 | 19 | 19.010 | 1 | .990 | 20 |
|  | 5 | 12 | 11.247 | 0 | .753 | 12 |
|  | 6 | 16 | 14.792 | 0 | 1.208 | 16 |
|  | 7 | 14 | 14.438 | 2 | 1.562 | 16 |
|  | 8 | 14 | 15.052 | 5 | 3.948 | 19 |
|  | 9 | 14 | 14.030 | 7 | 6.970 | 21 |

| **Classification Table**^a^ |  |  |  |  |  |
| --- | --- | --- | --- | --- | --- |
|  | Observed |  | Predicted |  |  |
|  |  |  | BFW_Lega |  | Percentage Correct |
|  |  |  | 0 | 1 |  |
| Step 1 | BFW_Lega | 0 | 122 | 2 | 98.4 |
|  |  | 1 | 14 | 2 | 12.5 |
|  | Overall Percentage |  |  |  | 88.6 |

| a. The cut value is .500 |  |  |  |  |  |
| --- | --- | --- | --- | --- | --- |

| **Variables in the Equation** |  |  |  |  |  |  |  |
| --- | --- | --- | --- | --- | --- | --- | --- |
|  |  | B | S.E. | Wald | df | Sig. | Exp(B) |
|  |  |  |  |  |  |  |  |
| Step 1^a^ | Career_stage(1) | -.184 | .678 | .074 | 1 | .786 | .832 |
|  | Age_category |  |  | 4.979 | 4 | .289 |  |
|  | Age_category(1) | .480 | 1.187 | .163 | 1 | .686 | 1.616 |
|  | Age_category(2) | 1.781 | 1.175 | 2.298 | 1 | .130 | 5.933 |
|  | Age_category(3) | .526 | 1.542 | .116 | 1 | .733 | 1.692 |
|  | Age_category(4) | 1.777 | 1.602 | 1.231 | 1 | .267 | 5.910 |
|  | Gender_ID |  |  | 8.433 | 3 | .038 |  |
|  | Gender_ID(1) | -1.777 | .822 | 4.668 | 1 | .031 | .169 |
|  | Gender_ID(2) | .149 | 1.203 | .015 | 1 | .901 | 1.161 |
|  | Gender_ID(3) | 1.197 | .792 | 2.283 | 1 | .131 | 3.311 |
|  | Constant | -2.704 | 1.060 | 6.506 | 1 | .011 | .067 |

| **Variables in the Equation** |  |  |  |
| --- | --- | --- | --- |
|  |  | 95% C.I.for EXP(B) |  |
|  |  | Lower | Upper |
| Step 1^a^ | Career_stage(1) | .220 | 3.142 |
|  | Age_category |  |  |
|  | Age_category(1) | .158 | 16.540 |
|  | Age_category(2) | .593 | 59.315 |
|  | Age_category(3) | .082 | 34.770 |
|  | Age_category(4) | .256 | 136.393 |
|  | Gender_ID |  |  |
|  | Gender_ID(1) | .034 | .848 |
|  | Gender_ID(2) | .110 | 12.268 |
|  | Gender_ID(3) | .701 | 15.647 |
|  | Constant |  |  |

|  |  |  |  |  |  |  |  |
| --- | --- | --- | --- | --- | --- | --- | --- |

| a. Variable(s) entered on step 1: Career_stage, Age_category, Gender_ID. |  |  |  |
| --- | --- | --- | --- |
