# Supplementary material for: #Yourpalaeolife: Interrogating the Status of Fieldwork Among Early Career Palaeontology Researchers
Source: Ecol Evol. 2026 Jul 29;16(8):e74032. doi: 10.1002/ece3.74032 (PMC13420382; doi:10.1002/ece3.74032)
Supplement: Supplementary file 1 — Data S1: ece374032‐sup‐0001‐Supinfo1.zip. [file ECE3-16-e74032-s003.zip › M38 BLR_BarriersFW_TrFSxRC.docx]

**Logistic Regression**

| **Notes** |  |  |
| --- | --- | --- |
| Output Created |  | 03-FEB-2026 15:45:13 |
| Comments |  |  |
| Input | Active Dataset | DataSet6 |
|  | Filter | <none> |
|  | Weight | <none> |
|  | Split File | <none> |
|  | N of Rows in Working Data File | 157 |
| Missing Value Handling | Definition of Missing | User-defined missing values are treated as missing |
| Syntax |  | LOGISTIC REGRESSION VARIABLES BFW_TrFS /METHOD=ENTER Career_stage Age_category Gender_ID /CONTRAST (Career_stage)=Indicator(1) /CONTRAST (Age_category)=Indicator(1) /CONTRAST (Gender_ID)=Indicator(1) /PRINT=GOODFIT CI(95) /CRITERIA=PIN(0.05) POUT(0.10) ITERATE(20) CUT(0.5). |
| Resources | Processor Time | 00:00:00.00 |
|  | Elapsed Time | 00:00:00.01 |

| **Warnings** |
| --- |
| Text: Career_stage Command: LOGISTIC REGRESSION This procedure cannot use string variables longer than 8 bytes. The values will be truncated. |
| Text: Age_category Command: LOGISTIC REGRESSION This procedure cannot use string variables longer than 8 bytes. The values will be truncated. |

| **Case Processing Summary** |  |  |  |
| --- | --- | --- | --- |
| Unweighted Cases^a^ |  | N | Percent |
| Selected Cases | Included in Analysis | 140 | 89.2 |
|  | Missing Cases | 17 | 10.8 |
|  | Total | 157 | 100.0 |
| Unselected Cases |  | 0 | .0 |
| Total |  | 157 | 100.0 |

| a. If weight is in effect, see classification table for the total number of cases. |  |  |  |
| --- | --- | --- | --- |

| **Dependent Variable Encoding** |  |
| --- | --- |
| Original Value | Internal Value |
| 0 | 0 |
| 1 | 1 |

| **Categorical Variables Codings** |  |  |  |  |  |  |
| --- | --- | --- | --- | --- | --- | --- |
|  |  | Frequency | Parameter coding |  |  |  |
|  |  |  | (1) | (2) | (3) | (4) |
| Age_category | <25 year | 18 | .000 | .000 | .000 | .000 |
|  | 26-30 ye | 51 | 1.000 | .000 | .000 | .000 |
|  | 31-35 ye | 46 | .000 | 1.000 | .000 | .000 |
|  | 36-40 ye | 18 | .000 | .000 | 1.000 | .000 |
|  | 41+ year | 7 | .000 | .000 | .000 | 1.000 |
| Gender_ID | F | 60 | .000 | .000 | .000 |  |
|  | M | 63 | 1.000 | .000 | .000 |  |
|  | N | 6 | .000 | 1.000 | .000 |  |
|  | U | 11 | .000 | .000 | 1.000 |  |
| Career_stage | PhD cand | 81 | .000 |  |  |  |
|  | Research | 59 | 1.000 |  |  |  |

**Block 0: Beginning Block**

| **Classification Table**^a,b^ |  |  |  |  |  |
| --- | --- | --- | --- | --- | --- |
|  | Observed |  | Predicted |  |  |
|  |  |  | BFW_TrFS |  | Percentage Correct |
|  |  |  | 0 | 1 |  |
| Step 0 | BFW_TrFS | 0 | 121 | 0 | 100.0 |
|  |  | 1 | 19 | 0 | .0 |
|  | Overall Percentage |  |  |  | 86.4 |

| a. Constant is included in the model. |  |  |  |  |  |
| --- | --- | --- | --- | --- | --- |
| b. The cut value is .500 |  |  |  |  |  |

| **Variables in the Equation** |  |  |  |  |  |  |  |
| --- | --- | --- | --- | --- | --- | --- | --- |
|  |  | B | S.E. | Wald | df | Sig. | Exp(B) |
| Step 0 | Constant | -1.851 | .247 | 56.284 | 1 | <.001 | .157 |

| **Variables not in the Equation** |  |  |  |  |  |
| --- | --- | --- | --- | --- | --- |
|  |  |  | Score | df | Sig. |
| Step 0 | Variables | Career_stage(1) | .246 | 1 | .620 |
|  |  | Age_category | 4.229 | 4 | .376 |
|  |  | Age_category(1) | 2.244 | 1 | .134 |
|  |  | Age_category(2) | 2.098 | 1 | .147 |
|  |  | Age_category(3) | .169 | 1 | .681 |
|  |  | Age_category(4) | 1.157 | 1 | .282 |
|  |  | Gender_ID | 5.771 | 3 | .123 |
|  |  | Gender_ID(1) | 5.094 | 1 | .024 |
|  |  | Gender_ID(2) | .051 | 1 | .821 |
|  |  | Gender_ID(3) | 1.911 | 1 | .167 |
|  | Overall Statistics |  | 10.667 | 8 | .221 |

**Block 1: Method = Enter**

| **Omnibus Tests of Model Coefficients** |  |  |  |  |
| --- | --- | --- | --- | --- |
|  |  | Chi-square | df | Sig. |
| Step 1 | Step | 12.156 | 8 | .144 |
|  | Block | 12.156 | 8 | .144 |
|  | Model | 12.156 | 8 | .144 |

| **Model Summary** |  |  |  |
| --- | --- | --- | --- |
| Step | -2 Log likelihood | Cox & Snell R Square | Nagelkerke R Square |
| 1 | 99.034^a^ | .083 | .152 |

| a. Estimation terminated at iteration number 20 because maximum iterations has been reached. Final solution cannot be found. |  |  |  |
| --- | --- | --- | --- |

| **Hosmer and Lemeshow Test** |  |  |  |
| --- | --- | --- | --- |
| Step | Chi-square | df | Sig. |
| 1 | 4.043 | 7 | .775 |

| **Contingency Table for Hosmer and Lemeshow Test** |  |  |  |  |  |  |
| --- | --- | --- | --- | --- | --- | --- |
|  |  | BFW_TrFS = 0 |  | BFW_TrFS = 1 |  | Total |
|  |  | Observed | Expected | Observed | Expected |  |
| Step 1 | 1 | 21 | 20.646 | 0 | .354 | 21 |
|  | 2 | 15 | 14.228 | 0 | .772 | 15 |
|  | 3 | 21 | 20.181 | 1 | 1.819 | 22 |
|  | 4 | 10 | 10.798 | 2 | 1.202 | 12 |
|  | 5 | 13 | 13.426 | 2 | 1.574 | 15 |
|  | 6 | 7 | 8.423 | 3 | 1.577 | 10 |
|  | 7 | 11 | 11.234 | 3 | 2.766 | 14 |
|  | 8 | 10 | 9.947 | 3 | 3.053 | 13 |
|  | 9 | 13 | 12.117 | 5 | 5.883 | 18 |

| **Classification Table**^a^ |  |  |  |  |  |
| --- | --- | --- | --- | --- | --- |
|  | Observed |  | Predicted |  |  |
|  |  |  | BFW_TrFS |  | Percentage Correct |
|  |  |  | 0 | 1 |  |
| Step 1 | BFW_TrFS | 0 | 121 | 0 | 100.0 |
|  |  | 1 | 19 | 0 | .0 |
|  | Overall Percentage |  |  |  | 86.4 |

| a. The cut value is .500 |  |  |  |  |  |
| --- | --- | --- | --- | --- | --- |

| **Variables in the Equation** |  |  |  |  |  |  |  |
| --- | --- | --- | --- | --- | --- | --- | --- |
|  |  | B | S.E. | Wald | df | Sig. | Exp(B) |
|  |  |  |  |  |  |  |  |
| Step 1^a^ | Career_stage(1) | .562 | .646 | .758 | 1 | .384 | 1.755 |
|  | Age_category |  |  | 2.247 | 4 | .690 |  |
|  | Age_category(1) | -.905 | .862 | 1.103 | 1 | .294 | .405 |
|  | Age_category(2) | .041 | .859 | .002 | 1 | .962 | 1.041 |
|  | Age_category(3) | .017 | 1.032 | .000 | 1 | .987 | 1.017 |
|  | Age_category(4) | -19.606 | 14733.856 | .000 | 1 | .999 | .000 |
|  | Gender_ID |  |  | 5.989 | 3 | .112 |  |
|  | Gender_ID(1) | -1.341 | .644 | 4.339 | 1 | .037 | .262 |
|  | Gender_ID(2) | .025 | 1.183 | .000 | 1 | .983 | 1.025 |
|  | Gender_ID(3) | .705 | .814 | .750 | 1 | .387 | 2.023 |
|  | Constant | -1.406 | .662 | 4.502 | 1 | .034 | .245 |

| **Variables in the Equation** |  |  |  |
| --- | --- | --- | --- |
|  |  | 95% C.I.for EXP(B) |  |
|  |  | Lower | Upper |
| Step 1^a^ | Career_stage(1) | .495 | 6.224 |
|  | Age_category |  |  |
|  | Age_category(1) | .075 | 2.191 |
|  | Age_category(2) | .194 | 5.604 |
|  | Age_category(3) | .135 | 7.690 |
|  | Age_category(4) | .000 | . |
|  | Gender_ID |  |  |
|  | Gender_ID(1) | .074 | .924 |
|  | Gender_ID(2) | .101 | 10.422 |
|  | Gender_ID(3) | .411 | 9.969 |
|  | Constant |  |  |

|  |  |  |  |  |  |  |  |
| --- | --- | --- | --- | --- | --- | --- | --- |

| a. Variable(s) entered on step 1: Career_stage, Age_category, Gender_ID. |  |  |  |
| --- | --- | --- | --- |
