# Supplementary material for: #Yourpalaeolife: Interrogating the Status of Fieldwork Among Early Career Palaeontology Researchers
Source: Ecol Evol. 2026 Jul 29;16(8):e74032. doi: 10.1002/ece3.74032 (PMC13420382; doi:10.1002/ece3.74032)
Supplement: Supplementary file 1 — Data S1: ece374032‐sup‐0001‐Supinfo1.zip. [file ECE3-16-e74032-s003.zip › M44 BLR_DiscFS_GendxRC.docx]

**Logistic Regression**

| **Notes** |  |  |
| --- | --- | --- |
| Output Created |  | 03-FEB-2026 15:56:12 |
| Comments |  |  |
| Input | Active Dataset | DataSet7 |
|  | Filter | <none> |
|  | Weight | <none> |
|  | Split File | <none> |
|  | N of Rows in Working Data File | 157 |
| Missing Value Handling | Definition of Missing | User-defined missing values are treated as missing |
| Syntax |  | LOGISTIC REGRESSION VARIABLES DTR_Gend /METHOD=ENTER Career_stage Age_category Gender_ID /CONTRAST (Career_stage)=Indicator(1) /CONTRAST (Age_category)=Indicator(1) /CONTRAST (Gender_ID)=Indicator(1) /PRINT=GOODFIT CI(95) /CRITERIA=PIN(0.05) POUT(0.10) ITERATE(20) CUT(0.5). |
| Resources | Processor Time | 00:00:00.02 |
|  | Elapsed Time | 00:00:00.01 |

| **Warnings** |
| --- |
| Text: Career_stage Command: LOGISTIC REGRESSION This procedure cannot use string variables longer than 8 bytes. The values will be truncated. |
| Text: Age_category Command: LOGISTIC REGRESSION This procedure cannot use string variables longer than 8 bytes. The values will be truncated. |

| **Case Processing Summary** |  |  |  |
| --- | --- | --- | --- |
| Unweighted Cases^a^ |  | N | Percent |
| Selected Cases | Included in Analysis | 142 | 90.4 |
|  | Missing Cases | 15 | 9.6 |
|  | Total | 157 | 100.0 |
| Unselected Cases |  | 0 | .0 |
| Total |  | 157 | 100.0 |

| a. If weight is in effect, see classification table for the total number of cases. |  |  |  |
| --- | --- | --- | --- |

| **Dependent Variable Encoding** |  |
| --- | --- |
| Original Value | Internal Value |
| 0 | 0 |
| 1 | 1 |

| **Categorical Variables Codings** |  |  |  |  |  |  |
| --- | --- | --- | --- | --- | --- | --- |
|  |  | Frequency | Parameter coding |  |  |  |
|  |  |  | (1) | (2) | (3) | (4) |
| Age_category | <25 year | 19 | .000 | .000 | .000 | .000 |
|  | 26-30 ye | 54 | 1.000 | .000 | .000 | .000 |
|  | 31-35 ye | 44 | .000 | 1.000 | .000 | .000 |
|  | 36-40 ye | 17 | .000 | .000 | 1.000 | .000 |
|  | 41+ year | 8 | .000 | .000 | .000 | 1.000 |
| Gender_ID | F | 59 | .000 | .000 | .000 |  |
|  | M | 66 | 1.000 | .000 | .000 |  |
|  | N | 5 | .000 | 1.000 | .000 |  |
|  | U | 12 | .000 | .000 | 1.000 |  |
| Career_stage | PhD cand | 81 | .000 |  |  |  |
|  | Research | 61 | 1.000 |  |  |  |

**Block 0: Beginning Block**

| **Classification Table**^a,b^ |  |  |  |  |  |
| --- | --- | --- | --- | --- | --- |
|  | Observed |  | Predicted |  |  |
|  |  |  | DTR_Gend |  | Percentage Correct |
|  |  |  | 0 | 1 |  |
| Step 0 | DTR_Gend | 0 | 113 | 0 | 100.0 |
|  |  | 1 | 29 | 0 | .0 |
|  | Overall Percentage |  |  |  | 79.6 |

| a. Constant is included in the model. |  |  |  |  |  |
| --- | --- | --- | --- | --- | --- |
| b. The cut value is .500 |  |  |  |  |  |

| **Variables in the Equation** |  |  |  |  |  |  |  |
| --- | --- | --- | --- | --- | --- | --- | --- |
|  |  | B | S.E. | Wald | df | Sig. | Exp(B) |
| Step 0 | Constant | -1.360 | .208 | 42.690 | 1 | <.001 | .257 |

| **Variables not in the Equation** |  |  |  |  |  |
| --- | --- | --- | --- | --- | --- |
|  |  |  | Score | df | Sig. |
| Step 0 | Variables | Career_stage(1) | 1.143 | 1 | .285 |
|  |  | Age_category | 1.832 | 4 | .767 |
|  |  | Age_category(1) | .756 | 1 | .384 |
|  |  | Age_category(2) | .822 | 1 | .365 |
|  |  | Age_category(3) | .092 | 1 | .762 |
|  |  | Age_category(4) | .327 | 1 | .567 |
|  |  | Gender_ID | 34.268 | 3 | <.001 |
|  |  | Gender_ID(1) | 27.126 | 1 | <.001 |
|  |  | Gender_ID(2) | 1.222 | 1 | .269 |
|  |  | Gender_ID(3) | 1.179 | 1 | .278 |
|  | Overall Statistics |  | 38.751 | 8 | <.001 |

**Block 1: Method = Enter**

| **Omnibus Tests of Model Coefficients** |  |  |  |  |
| --- | --- | --- | --- | --- |
|  |  | Chi-square | df | Sig. |
| Step 1 | Step | 45.886 | 8 | <.001 |
|  | Block | 45.886 | 8 | <.001 |
|  | Model | 45.886 | 8 | <.001 |

| **Model Summary** |  |  |  |
| --- | --- | --- | --- |
| Step | -2 Log likelihood | Cox & Snell R Square | Nagelkerke R Square |
| 1 | 97.876^a^ | .276 | .434 |

| a. Estimation terminated at iteration number 7 because parameter estimates changed by less than .001. |  |  |  |
| --- | --- | --- | --- |

| **Hosmer and Lemeshow Test** |  |  |  |
| --- | --- | --- | --- |
| Step | Chi-square | df | Sig. |
| 1 | 2.867 | 8 | .942 |

| **Contingency Table for Hosmer and Lemeshow Test** |  |  |  |  |  |  |
| --- | --- | --- | --- | --- | --- | --- |
|  |  | DTR_Gend = 0 |  | DTR_Gend = 1 |  | Total |
|  |  | Observed | Expected | Observed | Expected |  |
| Step 1 | 1 | 19 | 18.901 | 0 | .099 | 19 |
|  | 2 | 15 | 14.863 | 0 | .137 | 15 |
|  | 3 | 17 | 17.629 | 1 | .371 | 18 |
|  | 4 | 14 | 13.607 | 0 | .393 | 14 |
|  | 5 | 14 | 13.331 | 1 | 1.669 | 15 |
|  | 6 | 9 | 10.329 | 5 | 3.671 | 14 |
|  | 7 | 7 | 7.324 | 4 | 3.676 | 11 |
|  | 8 | 9 | 8.405 | 5 | 5.595 | 14 |
|  | 9 | 5 | 4.752 | 6 | 6.248 | 11 |
|  | 10 | 4 | 3.859 | 7 | 7.141 | 11 |

| **Classification Table**^a^ |  |  |  |  |  |
| --- | --- | --- | --- | --- | --- |
|  | Observed |  | Predicted |  |  |
|  |  |  | DTR_Gend |  | Percentage Correct |
|  |  |  | 0 | 1 |  |
| Step 1 | DTR_Gend | 0 | 104 | 9 | 92.0 |
|  |  | 1 | 16 | 13 | 44.8 |
|  | Overall Percentage |  |  |  | 82.4 |

| a. The cut value is .500 |  |  |  |  |  |
| --- | --- | --- | --- | --- | --- |

| **Variables in the Equation** |  |  |  |  |  |  |  |
| --- | --- | --- | --- | --- | --- | --- | --- |
|  |  | B | S.E. | Wald | df | Sig. | Exp(B) |
|  |  |  |  |  |  |  |  |
| Step 1^a^ | Career_stage(1) | 1.341 | .604 | 4.929 | 1 | .026 | 3.824 |
|  | Age_category |  |  | 1.259 | 4 | .868 |  |
|  | Age_category(1) | -.612 | .749 | .668 | 1 | .414 | .542 |
|  | Age_category(2) | -.303 | .799 | .144 | 1 | .705 | .739 |
|  | Age_category(3) | -.629 | 1.081 | .339 | 1 | .561 | .533 |
|  | Age_category(4) | -1.300 | 1.447 | .808 | 1 | .369 | .272 |
|  | Gender_ID |  |  | 18.014 | 3 | <.001 |  |
|  | Gender_ID(1) | -4.160 | 1.068 | 15.173 | 1 | <.001 | .016 |
|  | Gender_ID(2) | .440 | .995 | .196 | 1 | .658 | 1.553 |
|  | Gender_ID(3) | -2.007 | 1.111 | 3.265 | 1 | .071 | .134 |
|  | Constant | -.423 | .583 | .526 | 1 | .468 | .655 |

| **Variables in the Equation** |  |  |  |
| --- | --- | --- | --- |
|  |  | 95% C.I.for EXP(B) |  |
|  |  | Lower | Upper |
| Step 1^a^ | Career_stage(1) | 1.170 | 12.495 |
|  | Age_category |  |  |
|  | Age_category(1) | .125 | 2.353 |
|  | Age_category(2) | .154 | 3.534 |
|  | Age_category(3) | .064 | 4.433 |
|  | Age_category(4) | .016 | 4.644 |
|  | Gender_ID |  |  |
|  | Gender_ID(1) | .002 | .127 |
|  | Gender_ID(2) | .221 | 10.908 |
|  | Gender_ID(3) | .015 | 1.185 |
|  | Constant |  |  |

|  |  |  |  |  |  |  |  |
| --- | --- | --- | --- | --- | --- | --- | --- |

| a. Variable(s) entered on step 1: Career_stage, Age_category, Gender_ID. |  |  |  |
| --- | --- | --- | --- |
