# Supplementary material for: #Yourpalaeolife: Interrogating the Status of Fieldwork Among Early Career Palaeontology Researchers
Source: Ecol Evol. 2026 Jul 29;16(8):e74032. doi: 10.1002/ece3.74032 (PMC13420382; doi:10.1002/ece3.74032)
Supplement: Supplementary file 1 — Data S1: ece374032‐sup‐0001‐Supinfo1.zip. [file ECE3-16-e74032-s003.zip › M46 BLR_DiscFS_InterxRC.docx]

**Logistic Regression**

| **Notes** |  |  |
| --- | --- | --- |
| Output Created |  | 03-FEB-2026 16:01:03 |
| Comments |  |  |
| Input | Active Dataset | DataSet7 |
|  | Filter | <none> |
|  | Weight | <none> |
|  | Split File | <none> |
|  | N of Rows in Working Data File | 157 |
| Missing Value Handling | Definition of Missing | User-defined missing values are treated as missing |
| Syntax |  | LOGISTIC REGRESSION VARIABLES DTR_Inter /METHOD=ENTER Career_stage Age_category Gender_ID /CONTRAST (Career_stage)=Indicator(1) /CONTRAST (Age_category)=Indicator(1) /CONTRAST (Gender_ID)=Indicator(1) /PRINT=GOODFIT CI(95) /CRITERIA=PIN(0.05) POUT(0.10) ITERATE(20) CUT(0.5). |
| Resources | Processor Time | 00:00:00.02 |
|  | Elapsed Time | 00:00:00.01 |

| **Warnings** |
| --- |
| Text: Career_stage Command: LOGISTIC REGRESSION This procedure cannot use string variables longer than 8 bytes. The values will be truncated. |
| Text: Age_category Command: LOGISTIC REGRESSION This procedure cannot use string variables longer than 8 bytes. The values will be truncated. |

| **Case Processing Summary** |  |  |  |
| --- | --- | --- | --- |
| Unweighted Cases^a^ |  | N | Percent |
| Selected Cases | Included in Analysis | 142 | 90.4 |
|  | Missing Cases | 15 | 9.6 |
|  | Total | 157 | 100.0 |
| Unselected Cases |  | 0 | .0 |
| Total |  | 157 | 100.0 |

| a. If weight is in effect, see classification table for the total number of cases. |  |  |  |
| --- | --- | --- | --- |

| **Dependent Variable Encoding** |  |
| --- | --- |
| Original Value | Internal Value |
| 0 | 0 |
| 1 | 1 |

| **Categorical Variables Codings** |  |  |  |  |  |  |
| --- | --- | --- | --- | --- | --- | --- |
|  |  | Frequency | Parameter coding |  |  |  |
|  |  |  | (1) | (2) | (3) | (4) |
| Age_category | <25 year | 19 | .000 | .000 | .000 | .000 |
|  | 26-30 ye | 54 | 1.000 | .000 | .000 | .000 |
|  | 31-35 ye | 44 | .000 | 1.000 | .000 | .000 |
|  | 36-40 ye | 17 | .000 | .000 | 1.000 | .000 |
|  | 41+ year | 8 | .000 | .000 | .000 | 1.000 |
| Gender_ID | F | 59 | .000 | .000 | .000 |  |
|  | M | 66 | 1.000 | .000 | .000 |  |
|  | N | 5 | .000 | 1.000 | .000 |  |
|  | U | 12 | .000 | .000 | 1.000 |  |
| Career_stage | PhD cand | 81 | .000 |  |  |  |
|  | Research | 61 | 1.000 |  |  |  |

**Block 0: Beginning Block**

| **Classification Table**^a,b^ |  |  |  |  |  |
| --- | --- | --- | --- | --- | --- |
|  | Observed |  | Predicted |  |  |
|  |  |  | DTR_Inter |  | Percentage Correct |
|  |  |  | 0 | 1 |  |
| Step 0 | DTR_Inter | 0 | 119 | 0 | 100.0 |
|  |  | 1 | 23 | 0 | .0 |
|  | Overall Percentage |  |  |  | 83.8 |

| a. Constant is included in the model. |  |  |  |  |  |
| --- | --- | --- | --- | --- | --- |
| b. The cut value is .500 |  |  |  |  |  |

| **Variables in the Equation** |  |  |  |  |  |  |  |
| --- | --- | --- | --- | --- | --- | --- | --- |
|  |  | B | S.E. | Wald | df | Sig. | Exp(B) |
| Step 0 | Constant | -1.644 | .228 | 52.071 | 1 | <.001 | .193 |

| **Variables not in the Equation** |  |  |  |  |  |
| --- | --- | --- | --- | --- | --- |
|  |  |  | Score | df | Sig. |
| Step 0 | Variables | Career_stage(1) | 5.550 | 1 | .018 |
|  |  | Age_category | 16.579 | 4 | .002 |
|  |  | Age_category(1) | 10.020 | 1 | .002 |
|  |  | Age_category(2) | 3.640 | 1 | .056 |
|  |  | Age_category(3) | .765 | 1 | .382 |
|  |  | Age_category(4) | 7.136 | 1 | .008 |
|  |  | Gender_ID | 1.866 | 3 | .601 |
|  |  | Gender_ID(1) | 1.113 | 1 | .291 |
|  |  | Gender_ID(2) | 1.002 | 1 | .317 |
|  |  | Gender_ID(3) | .002 | 1 | .963 |
|  | Overall Statistics |  | 18.967 | 8 | .015 |

**Block 1: Method = Enter**

| **Omnibus Tests of Model Coefficients** |  |  |  |  |
| --- | --- | --- | --- | --- |
|  |  | Chi-square | df | Sig. |
| Step 1 | Step | 20.196 | 8 | .010 |
|  | Block | 20.196 | 8 | .010 |
|  | Model | 20.196 | 8 | .010 |

| **Model Summary** |  |  |  |
| --- | --- | --- | --- |
| Step | -2 Log likelihood | Cox & Snell R Square | Nagelkerke R Square |
| 1 | 105.595^a^ | .133 | .226 |

| a. Estimation terminated at iteration number 20 because maximum iterations has been reached. Final solution cannot be found. |  |  |  |
| --- | --- | --- | --- |

| **Hosmer and Lemeshow Test** |  |  |  |
| --- | --- | --- | --- |
| Step | Chi-square | df | Sig. |
| 1 | 5.846 | 8 | .664 |

| **Contingency Table for Hosmer and Lemeshow Test** |  |  |  |  |  |  |
| --- | --- | --- | --- | --- | --- | --- |
|  |  | DTR_Inter = 0 |  | DTR_Inter = 1 |  | Total |
|  |  | Observed | Expected | Observed | Expected |  |
| Step 1 | 1 | 18 | 18.697 | 1 | .303 | 19 |
|  | 2 | 15 | 14.543 | 0 | .457 | 15 |
|  | 3 | 10 | 10.488 | 1 | .512 | 11 |
|  | 4 | 9 | 8.469 | 0 | .531 | 9 |
|  | 5 | 13 | 13.530 | 2 | 1.470 | 15 |
|  | 6 | 14 | 12.072 | 0 | 1.928 | 14 |
|  | 7 | 10 | 10.428 | 3 | 2.572 | 13 |
|  | 8 | 8 | 8.219 | 3 | 2.781 | 11 |
|  | 9 | 8 | 8.560 | 4 | 3.440 | 12 |
|  | 10 | 14 | 13.993 | 9 | 9.007 | 23 |

| **Classification Table**^a^ |  |  |  |  |  |
| --- | --- | --- | --- | --- | --- |
|  | Observed |  | Predicted |  |  |
|  |  |  | DTR_Inter |  | Percentage Correct |
|  |  |  | 0 | 1 |  |
| Step 1 | DTR_Inter | 0 | 117 | 2 | 98.3 |
|  |  | 1 | 20 | 3 | 13.0 |
|  | Overall Percentage |  |  |  | 84.5 |

| a. The cut value is .500 |  |  |  |  |  |
| --- | --- | --- | --- | --- | --- |

| **Variables in the Equation** |  |  |  |  |  |  |  |
| --- | --- | --- | --- | --- | --- | --- | --- |
|  |  | B | S.E. | Wald | df | Sig. | Exp(B) |
|  |  |  |  |  |  |  |  |
| Step 1^a^ | Career_stage(1) | .691 | .582 | 1.408 | 1 | .235 | 1.995 |
|  | Age_category |  |  | 9.822 | 4 | .044 |  |
|  | Age_category(1) | -1.541 | 1.097 | 1.973 | 1 | .160 | .214 |
|  | Age_category(2) | .497 | .924 | .290 | 1 | .590 | 1.644 |
|  | Age_category(3) | .301 | 1.068 | .080 | 1 | .778 | 1.352 |
|  | Age_category(4) | 1.617 | 1.104 | 2.145 | 1 | .143 | 5.040 |
|  | Gender_ID |  |  | 1.055 | 3 | .788 |  |
|  | Gender_ID(1) | .353 | .531 | .443 | 1 | .506 | 1.424 |
|  | Gender_ID(2) | -18.415 | 17128.876 | .000 | 1 | .999 | .000 |
|  | Gender_ID(3) | .905 | .955 | .898 | 1 | .343 | 2.472 |
|  | Constant | -2.272 | .791 | 8.244 | 1 | .004 | .103 |

| **Variables in the Equation** |  |  |  |
| --- | --- | --- | --- |
|  |  | 95% C.I.for EXP(B) |  |
|  |  | Lower | Upper |
| Step 1^a^ | Career_stage(1) | .637 | 6.247 |
|  | Age_category |  |  |
|  | Age_category(1) | .025 | 1.839 |
|  | Age_category(2) | .269 | 10.045 |
|  | Age_category(3) | .167 | 10.963 |
|  | Age_category(4) | .579 | 43.907 |
|  | Gender_ID |  |  |
|  | Gender_ID(1) | .503 | 4.034 |
|  | Gender_ID(2) | .000 | . |
|  | Gender_ID(3) | .380 | 16.073 |
|  | Constant |  |  |

|  |  |  |  |  |  |  |  |
| --- | --- | --- | --- | --- | --- | --- | --- |

| a. Variable(s) entered on step 1: Career_stage, Age_category, Gender_ID. |  |  |  |
| --- | --- | --- | --- |
