# Supplementary material for: #Yourpalaeolife: Interrogating the Status of Fieldwork Among Early Career Palaeontology Researchers
Source: Ecol Evol. 2026 Jul 29;16(8):e74032. doi: 10.1002/ece3.74032 (PMC13420382; doi:10.1002/ece3.74032)
Supplement: Supplementary file 1 — Data S1: ece374032‐sup‐0001‐Supinfo1.zip. [file ECE3-16-e74032-s003.zip › M50 BLR_DiscFS_NonexRC.docx]

**Logistic Regression**

| **Notes** |  |  |
| --- | --- | --- |
| Output Created |  | 03-FEB-2026 16:04:59 |
| Comments |  |  |
| Input | Active Dataset | DataSet7 |
|  | Filter | <none> |
|  | Weight | <none> |
|  | Split File | <none> |
|  | N of Rows in Working Data File | 157 |
| Missing Value Handling | Definition of Missing | User-defined missing values are treated as missing |
| Syntax |  | LOGISTIC REGRESSION VARIABLES DTR_NoDE /METHOD=ENTER Career_stage Age_category Gender_ID /CONTRAST (Career_stage)=Indicator(1) /CONTRAST (Age_category)=Indicator(1) /CONTRAST (Gender_ID)=Indicator(1) /PRINT=GOODFIT CI(95) /CRITERIA=PIN(0.05) POUT(0.10) ITERATE(20) CUT(0.5). |
| Resources | Processor Time | 00:00:00.02 |
|  | Elapsed Time | 00:00:00.01 |

| **Warnings** |
| --- |
| Text: Career_stage Command: LOGISTIC REGRESSION This procedure cannot use string variables longer than 8 bytes. The values will be truncated. |
| Text: Age_category Command: LOGISTIC REGRESSION This procedure cannot use string variables longer than 8 bytes. The values will be truncated. |

| **Case Processing Summary** |  |  |  |
| --- | --- | --- | --- |
| Unweighted Cases^a^ |  | N | Percent |
| Selected Cases | Included in Analysis | 142 | 90.4 |
|  | Missing Cases | 15 | 9.6 |
|  | Total | 157 | 100.0 |
| Unselected Cases |  | 0 | .0 |
| Total |  | 157 | 100.0 |

| a. If weight is in effect, see classification table for the total number of cases. |  |  |  |
| --- | --- | --- | --- |

| **Dependent Variable Encoding** |  |
| --- | --- |
| Original Value | Internal Value |
| 0 | 0 |
| 1 | 1 |

| **Categorical Variables Codings** |  |  |  |  |  |  |
| --- | --- | --- | --- | --- | --- | --- |
|  |  | Frequency | Parameter coding |  |  |  |
|  |  |  | (1) | (2) | (3) | (4) |
| Age_category | <25 year | 19 | .000 | .000 | .000 | .000 |
|  | 26-30 ye | 54 | 1.000 | .000 | .000 | .000 |
|  | 31-35 ye | 44 | .000 | 1.000 | .000 | .000 |
|  | 36-40 ye | 17 | .000 | .000 | 1.000 | .000 |
|  | 41+ year | 8 | .000 | .000 | .000 | 1.000 |
| Gender_ID | F | 59 | .000 | .000 | .000 |  |
|  | M | 66 | 1.000 | .000 | .000 |  |
|  | N | 5 | .000 | 1.000 | .000 |  |
|  | U | 12 | .000 | .000 | 1.000 |  |
| Career_stage | PhD cand | 81 | .000 |  |  |  |
|  | Research | 61 | 1.000 |  |  |  |

**Block 0: Beginning Block**

| **Classification Table**^a,b^ |  |  |  |  |  |
| --- | --- | --- | --- | --- | --- |
|  | Observed |  | Predicted |  |  |
|  |  |  | DTR_NoDE |  | Percentage Correct |
|  |  |  | 0 | 1 |  |
| Step 0 | DTR_NoDE | 0 | 0 | 65 | .0 |
|  |  | 1 | 0 | 77 | 100.0 |
|  | Overall Percentage |  |  |  | 54.2 |

| a. Constant is included in the model. |  |  |  |  |  |
| --- | --- | --- | --- | --- | --- |
| b. The cut value is .500 |  |  |  |  |  |

| **Variables in the Equation** |  |  |  |  |  |  |  |
| --- | --- | --- | --- | --- | --- | --- | --- |
|  |  | B | S.E. | Wald | df | Sig. | Exp(B) |
| Step 0 | Constant | .169 | .168 | 1.012 | 1 | .315 | 1.185 |

| **Variables not in the Equation** |  |  |  |  |  |
| --- | --- | --- | --- | --- | --- |
|  |  |  | Score | df | Sig. |
| Step 0 | Variables | Career_stage(1) | 1.097 | 1 | .295 |
|  |  | Age_category | 15.653 | 4 | .004 |
|  |  | Age_category(1) | 13.830 | 1 | <.001 |
|  |  | Age_category(2) | 4.555 | 1 | .033 |
|  |  | Age_category(3) | 1.325 | 1 | .250 |
|  |  | Age_category(4) | 2.917 | 1 | .088 |
|  |  | Gender_ID | 11.191 | 3 | .011 |
|  |  | Gender_ID(1) | 9.677 | 1 | .002 |
|  |  | Gender_ID(2) | .423 | 1 | .516 |
|  |  | Gender_ID(3) | .089 | 1 | .765 |
|  | Overall Statistics |  | 28.639 | 8 | <.001 |

**Block 1: Method = Enter**

| **Omnibus Tests of Model Coefficients** |  |  |  |  |
| --- | --- | --- | --- | --- |
|  |  | Chi-square | df | Sig. |
| Step 1 | Step | 31.430 | 8 | <.001 |
|  | Block | 31.430 | 8 | <.001 |
|  | Model | 31.430 | 8 | <.001 |

| **Model Summary** |  |  |  |
| --- | --- | --- | --- |
| Step | -2 Log likelihood | Cox & Snell R Square | Nagelkerke R Square |
| 1 | 164.409^a^ | .199 | .265 |

| a. Estimation terminated at iteration number 4 because parameter estimates changed by less than .001. |  |  |  |
| --- | --- | --- | --- |

| **Hosmer and Lemeshow Test** |  |  |  |
| --- | --- | --- | --- |
| Step | Chi-square | df | Sig. |
| 1 | 1.144 | 8 | .997 |

| **Contingency Table for Hosmer and Lemeshow Test** |  |  |  |  |  |  |
| --- | --- | --- | --- | --- | --- | --- |
|  |  | DTR_NoDE = 0 |  | DTR_NoDE = 1 |  | Total |
|  |  | Observed | Expected | Observed | Expected |  |
| Step 1 | 1 | 8 | 8.323 | 2 | 1.677 | 10 |
|  | 2 | 9 | 8.525 | 2 | 2.475 | 11 |
|  | 3 | 9 | 9.291 | 4 | 3.709 | 13 |
|  | 4 | 10 | 9.586 | 6 | 6.414 | 16 |
|  | 5 | 7 | 7.145 | 8 | 7.855 | 15 |
|  | 6 | 5 | 5.971 | 9 | 8.029 | 14 |
|  | 7 | 6 | 4.763 | 6 | 7.237 | 12 |
|  | 8 | 5 | 5.147 | 9 | 8.853 | 14 |
|  | 9 | 3 | 3.354 | 10 | 9.646 | 13 |
|  | 10 | 3 | 2.895 | 21 | 21.105 | 24 |

| **Classification Table**^a^ |  |  |  |  |  |
| --- | --- | --- | --- | --- | --- |
|  | Observed |  | Predicted |  |  |
|  |  |  | DTR_NoDE |  | Percentage Correct |
|  |  |  | 0 | 1 |  |
| Step 1 | DTR_NoDE | 0 | 36 | 29 | 55.4 |
|  |  | 1 | 14 | 63 | 81.8 |
|  | Overall Percentage |  |  |  | 69.7 |

| a. The cut value is .500 |  |  |  |  |  |
| --- | --- | --- | --- | --- | --- |

| **Variables in the Equation** |  |  |  |  |  |  |  |
| --- | --- | --- | --- | --- | --- | --- | --- |
|  |  | B | S.E. | Wald | df | Sig. | Exp(B) |
|  |  |  |  |  |  |  |  |
| Step 1^a^ | Career_stage(1) | -.224 | .446 | .252 | 1 | .615 | .799 |
|  | Age_category |  |  | 15.055 | 4 | .005 |  |
|  | Age_category(1) | .885 | .610 | 2.107 | 1 | .147 | 2.422 |
|  | Age_category(2) | -.670 | .649 | 1.068 | 1 | .301 | .512 |
|  | Age_category(3) | -.898 | .803 | 1.251 | 1 | .263 | .408 |
|  | Age_category(4) | -1.719 | 1.030 | 2.786 | 1 | .095 | .179 |
|  | Gender_ID |  |  | 13.549 | 3 | .004 |  |
|  | Gender_ID(1) | 1.533 | .434 | 12.484 | 1 | <.001 | 4.631 |
|  | Gender_ID(2) | -.512 | 1.009 | .258 | 1 | .612 | .599 |
|  | Gender_ID(3) | .472 | .696 | .461 | 1 | .497 | 1.603 |
|  | Constant | -.342 | .509 | .452 | 1 | .501 | .710 |

| **Variables in the Equation** |  |  |  |
| --- | --- | --- | --- |
|  |  | 95% C.I.for EXP(B) |  |
|  |  | Lower | Upper |
| Step 1^a^ | Career_stage(1) | .333 | 1.917 |
|  | Age_category |  |  |
|  | Age_category(1) | .733 | 7.998 |
|  | Age_category(2) | .144 | 1.824 |
|  | Age_category(3) | .085 | 1.965 |
|  | Age_category(4) | .024 | 1.349 |
|  | Gender_ID |  |  |
|  | Gender_ID(1) | 1.979 | 10.836 |
|  | Gender_ID(2) | .083 | 4.331 |
|  | Gender_ID(3) | .410 | 6.268 |
|  | Constant |  |  |

|  |  |  |  |  |  |  |  |
| --- | --- | --- | --- | --- | --- | --- | --- |

| a. Variable(s) entered on step 1: Career_stage, Age_category, Gender_ID. |  |  |  |
| --- | --- | --- | --- |
