# Supplementary material for: #Yourpalaeolife: Interrogating the Status of Fieldwork Among Early Career Palaeontology Researchers
Source: Ecol Evol. 2026 Jul 29;16(8):e74032. doi: 10.1002/ece3.74032 (PMC13420382; doi:10.1002/ece3.74032)
Supplement: Supplementary file 1 — Data S1: ece374032‐sup‐0001‐Supinfo1.zip. [file ECE3-16-e74032-s003.zip › M8 BLR_BarriersFS_DiscxRC.docx]

**Logistic Regression**

| **Notes** |  |  |
| --- | --- | --- |
| Output Created |  | 03-FEB-2026 15:04:25 |
| Comments |  |  |
| Input | Active Dataset | DataSet3 |
|  | Filter | <none> |
|  | Weight | <none> |
|  | Split File | <none> |
|  | N of Rows in Working Data File | 157 |
| Missing Value Handling | Definition of Missing | User-defined missing values are treated as missing |
| Syntax |  | LOGISTIC REGRESSION VARIABLES BTR_Disc /METHOD=ENTER Age_category Career_stage Gender_ID /CONTRAST (Age_category)=Indicator(1) /CONTRAST (Career_stage)=Indicator(1) /CONTRAST (Gender_ID)=Indicator(1) /PRINT=GOODFIT CI(95) /CRITERIA=PIN(0.05) POUT(0.10) ITERATE(20) CUT(0.5). |
| Resources | Processor Time | 00:00:00.00 |
|  | Elapsed Time | 00:00:00.01 |

| **Warnings** |
| --- |
| Text: Age_category Command: LOGISTIC REGRESSION This procedure cannot use string variables longer than 8 bytes. The values will be truncated. |
| Text: Career_stage Command: LOGISTIC REGRESSION This procedure cannot use string variables longer than 8 bytes. The values will be truncated. |

| **Case Processing Summary** |  |  |  |
| --- | --- | --- | --- |
| Unweighted Cases^a^ |  | N | Percent |
| Selected Cases | Included in Analysis | 156 | 99.4 |
|  | Missing Cases | 1 | .6 |
|  | Total | 157 | 100.0 |
| Unselected Cases |  | 0 | .0 |
| Total |  | 157 | 100.0 |

| a. If weight is in effect, see classification table for the total number of cases. |  |  |  |
| --- | --- | --- | --- |

| **Dependent Variable Encoding** |  |
| --- | --- |
| Original Value | Internal Value |
| 0 | 0 |
| 1 | 1 |

| **Categorical Variables Codings** |  |  |  |  |  |  |
| --- | --- | --- | --- | --- | --- | --- |
|  |  | Frequency | Parameter coding |  |  |  |
|  |  |  | (1) | (2) | (3) | (4) |
| Age_category | <25 year | 22 | .000 | .000 | .000 | .000 |
|  | 26-30 ye | 57 | 1.000 | .000 | .000 | .000 |
|  | 31-35 ye | 51 | .000 | 1.000 | .000 | .000 |
|  | 36-40 ye | 18 | .000 | .000 | 1.000 | .000 |
|  | 41+ year | 8 | .000 | .000 | .000 | 1.000 |
| Gender_ID | F | 68 | .000 | .000 | .000 |  |
|  | M | 69 | 1.000 | .000 | .000 |  |
|  | N | 6 | .000 | 1.000 | .000 |  |
|  | U | 13 | .000 | .000 | 1.000 |  |
| Career_stage | PhD cand | 88 | .000 |  |  |  |
|  | Research | 68 | 1.000 |  |  |  |

**Block 0: Beginning Block**

| **Classification Table**^a,b^ |  |  |  |  |  |
| --- | --- | --- | --- | --- | --- |
|  | Observed |  | Predicted |  |  |
|  |  |  | BTR_Disc |  | Percentage Correct |
|  |  |  | 0 | 1 |  |
| Step 0 | BTR_Disc | 0 | 142 | 0 | 100.0 |
|  |  | 1 | 14 | 0 | .0 |
|  | Overall Percentage |  |  |  | 91.0 |

| a. Constant is included in the model. |  |  |  |  |  |
| --- | --- | --- | --- | --- | --- |
| b. The cut value is .500 |  |  |  |  |  |

| **Variables in the Equation** |  |  |  |  |  |  |  |
| --- | --- | --- | --- | --- | --- | --- | --- |
|  |  | B | S.E. | Wald | df | Sig. | Exp(B) |
| Step 0 | Constant | -2.317 | .280 | 68.400 | 1 | <.001 | .099 |

| **Variables not in the Equation** |  |  |  |  |  |
| --- | --- | --- | --- | --- | --- |
|  |  |  | Score | df | Sig. |
| Step 0 | Variables | Age_category | 17.030 | 4 | .002 |
|  |  | Age_category(1) | 8.855 | 1 | .003 |
|  |  | Age_category(2) | 4.179 | 1 | .041 |
|  |  | Age_category(3) | .114 | 1 | .736 |
|  |  | Age_category(4) | 8.400 | 1 | .004 |
|  |  | Career_stage(1) | 4.848 | 1 | .028 |
|  |  | Gender_ID | .769 | 3 | .857 |
|  |  | Gender_ID(1) | .012 | 1 | .914 |
|  |  | Gender_ID(2) | .615 | 1 | .433 |
|  |  | Gender_ID(3) | .029 | 1 | .866 |
|  | Overall Statistics |  | 18.814 | 8 | .016 |

**Block 1: Method = Enter**

| **Omnibus Tests of Model Coefficients** |  |  |  |  |
| --- | --- | --- | --- | --- |
|  |  | Chi-square | df | Sig. |
| Step 1 | Step | 21.181 | 8 | .007 |
|  | Block | 21.181 | 8 | .007 |
|  | Model | 21.181 | 8 | .007 |

| **Model Summary** |  |  |  |
| --- | --- | --- | --- |
| Step | -2 Log likelihood | Cox & Snell R Square | Nagelkerke R Square |
| 1 | 73.026^a^ | .127 | .280 |

| a. Estimation terminated at iteration number 20 because maximum iterations has been reached. Final solution cannot be found. |  |  |  |
| --- | --- | --- | --- |

| **Hosmer and Lemeshow Test** |  |  |  |
| --- | --- | --- | --- |
| Step | Chi-square | df | Sig. |
| 1 | 1.885 | 7 | .966 |

| **Contingency Table for Hosmer and Lemeshow Test** |  |  |  |  |  |  |
| --- | --- | --- | --- | --- | --- | --- |
|  |  | BTR_Disc = 0 |  | BTR_Disc = 1 |  | Total |
|  |  | Observed | Expected | Observed | Expected |  |
| Step 1 | 1 | 18 | 18.000 | 0 | .000 | 18 |
|  | 2 | 18 | 18.000 | 0 | .000 | 18 |
|  | 3 | 14 | 14.000 | 0 | .000 | 14 |
|  | 4 | 16 | 15.803 | 0 | .197 | 16 |
|  | 5 | 15 | 15.174 | 1 | .826 | 16 |
|  | 6 | 16 | 16.347 | 2 | 1.653 | 18 |
|  | 7 | 7 | 7.968 | 2 | 1.032 | 9 |
|  | 8 | 14 | 14.364 | 3 | 2.636 | 17 |
|  | 9 | 24 | 22.345 | 6 | 7.655 | 30 |

| **Classification Table**^a^ |  |  |  |  |  |
| --- | --- | --- | --- | --- | --- |
|  | Observed |  | Predicted |  |  |
|  |  |  | BTR_Disc |  | Percentage Correct |
|  |  |  | 0 | 1 |  |
| Step 1 | BTR_Disc | 0 | 142 | 0 | 100.0 |
|  |  | 1 | 12 | 2 | 14.3 |
|  | Overall Percentage |  |  |  | 92.3 |

| a. The cut value is .500 |  |  |  |  |  |
| --- | --- | --- | --- | --- | --- |

| **Variables in the Equation** |  |  |  |  |  |  |  |
| --- | --- | --- | --- | --- | --- | --- | --- |
|  |  | B | S.E. | Wald | df | Sig. | Exp(B) |
|  |  |  |  |  |  |  |  |
| Step 1^a^ | Age_category |  |  | 3.756 | 4 | .440 |  |
|  | Age_category(1) | -18.419 | 5203.757 | .000 | 1 | .997 | .000 |
|  | Age_category(2) | .861 | 1.243 | .479 | 1 | .489 | 2.365 |
|  | Age_category(3) | .446 | 1.420 | .099 | 1 | .754 | 1.562 |
|  | Age_category(4) | 2.147 | 1.378 | 2.426 | 1 | .119 | 8.558 |
|  | Career_stage(1) | .827 | .763 | 1.176 | 1 | .278 | 2.287 |
|  | Gender_ID |  |  | .805 | 3 | .848 |  |
|  | Gender_ID(1) | -.477 | .642 | .552 | 1 | .457 | .621 |
|  | Gender_ID(2) | -18.429 | 14499.916 | .000 | 1 | .999 | .000 |
|  | Gender_ID(3) | .426 | 1.248 | .116 | 1 | .733 | 1.531 |
|  | Constant | -2.906 | 1.041 | 7.800 | 1 | .005 | .055 |

| **Variables in the Equation** |  |  |  |
| --- | --- | --- | --- |
|  |  | 95% C.I.for EXP(B) |  |
|  |  | Lower | Upper |
| Step 1^a^ | Age_category |  |  |
|  | Age_category(1) | .000 | . |
|  | Age_category(2) | .207 | 27.056 |
|  | Age_category(3) | .097 | 25.248 |
|  | Age_category(4) | .574 | 127.494 |
|  | Career_stage(1) | .513 | 10.192 |
|  | Gender_ID |  |  |
|  | Gender_ID(1) | .177 | 2.183 |
|  | Gender_ID(2) | .000 | . |
|  | Gender_ID(3) | .133 | 17.676 |
|  | Constant |  |  |

|  |  |  |  |  |  |  |  |
| --- | --- | --- | --- | --- | --- | --- | --- |

| a. Variable(s) entered on step 1: Age_category, Career_stage, Gender_ID. |  |  |  |
| --- | --- | --- | --- |
