# Supplementary material for: #Yourpalaeolife: Interrogating the Status of Fieldwork Among Early Career Palaeontology Researchers
Source: Ecol Evol. 2026 Jul 29;16(8):e74032. doi: 10.1002/ece3.74032 (PMC13420382; doi:10.1002/ece3.74032)
Supplement: Supplementary file 2 — Data S2: ece374032‐sup‐0002‐Supinfo2.zip. [file ECE3-16-e74032-s002.zip › M57 BLR_DiscFW_GendxRC.docx]

**Logistic Regression**

| **Notes** |  |  |
| --- | --- | --- |
| Output Created |  | 03-FEB-2026 16:08:07 |
| Comments |  |  |
| Input | Active Dataset | DataSet7 |
|  | Filter | <none> |
|  | Weight | <none> |
|  | Split File | <none> |
|  | N of Rows in Working Data File | 157 |
| Missing Value Handling | Definition of Missing | User-defined missing values are treated as missing |
| Syntax |  | LOGISTIC REGRESSION VARIABLES DFW_FwGe /METHOD=ENTER Career_stage Age_category Gender_ID /CONTRAST (Career_stage)=Indicator(1) /CONTRAST (Age_category)=Indicator(1) /CONTRAST (Gender_ID)=Indicator(1) /PRINT=GOODFIT CI(95) /CRITERIA=PIN(0.05) POUT(0.10) ITERATE(20) CUT(0.5). |
| Resources | Processor Time | 00:00:00.02 |
|  | Elapsed Time | 00:00:00.01 |

| **Warnings** |
| --- |
| Text: Career_stage Command: LOGISTIC REGRESSION This procedure cannot use string variables longer than 8 bytes. The values will be truncated. |
| Text: Age_category Command: LOGISTIC REGRESSION This procedure cannot use string variables longer than 8 bytes. The values will be truncated. |

| **Case Processing Summary** |  |  |  |
| --- | --- | --- | --- |
| Unweighted Cases^a^ |  | N | Percent |
| Selected Cases | Included in Analysis | 135 | 86.0 |
|  | Missing Cases | 22 | 14.0 |
|  | Total | 157 | 100.0 |
| Unselected Cases |  | 0 | .0 |
| Total |  | 157 | 100.0 |

| a. If weight is in effect, see classification table for the total number of cases. |  |  |  |
| --- | --- | --- | --- |

| **Dependent Variable Encoding** |  |
| --- | --- |
| Original Value | Internal Value |
| 0 | 0 |
| 1 | 1 |

| **Categorical Variables Codings** |  |  |  |  |  |  |
| --- | --- | --- | --- | --- | --- | --- |
|  |  | Frequency | Parameter coding |  |  |  |
|  |  |  | (1) | (2) | (3) | (4) |
| Age_category | <25 year | 20 | .000 | .000 | .000 | .000 |
|  | 26-30 ye | 53 | 1.000 | .000 | .000 | .000 |
|  | 31-35 ye | 40 | .000 | 1.000 | .000 | .000 |
|  | 36-40 ye | 15 | .000 | .000 | 1.000 | .000 |
|  | 41+ year | 7 | .000 | .000 | .000 | 1.000 |
| Gender_ID | F | 58 | .000 | .000 | .000 |  |
|  | M | 62 | 1.000 | .000 | .000 |  |
|  | N | 5 | .000 | 1.000 | .000 |  |
|  | U | 10 | .000 | .000 | 1.000 |  |
| Career_stage | PhD cand | 81 | .000 |  |  |  |
|  | Research | 54 | 1.000 |  |  |  |

**Block 0: Beginning Block**

| **Classification Table**^a,b^ |  |  |  |  |  |
| --- | --- | --- | --- | --- | --- |
|  | Observed |  | Predicted |  |  |
|  |  |  | DFW_FwGe |  | Percentage Correct |
|  |  |  | 0 | 1 |  |
| Step 0 | DFW_FwGe | 0 | 107 | 0 | 100.0 |
|  |  | 1 | 28 | 0 | .0 |
|  | Overall Percentage |  |  |  | 79.3 |

| a. Constant is included in the model. |  |  |  |  |  |
| --- | --- | --- | --- | --- | --- |
| b. The cut value is .500 |  |  |  |  |  |

| **Variables in the Equation** |  |  |  |  |  |  |  |
| --- | --- | --- | --- | --- | --- | --- | --- |
|  |  | B | S.E. | Wald | df | Sig. | Exp(B) |
| Step 0 | Constant | -1.341 | .212 | 39.886 | 1 | <.001 | .262 |

| **Variables not in the Equation** |  |  |  |  |  |
| --- | --- | --- | --- | --- | --- |
|  |  |  | Score | df | Sig. |
| Step 0 | Variables | Career_stage(1) | .120 | 1 | .729 |
|  |  | Age_category | 5.736 | 4 | .220 |
|  |  | Age_category(1) | 1.692 | 1 | .193 |
|  |  | Age_category(2) | .019 | 1 | .890 |
|  |  | Age_category(3) | .006 | 1 | .940 |
|  |  | Age_category(4) | .187 | 1 | .665 |
|  |  | Gender_ID | 23.128 | 3 | <.001 |
|  |  | Gender_ID(1) | 21.397 | 1 | <.001 |
|  |  | Gender_ID(2) | 1.172 | 1 | .279 |
|  |  | Gender_ID(3) | .004 | 1 | .952 |
|  | Overall Statistics |  | 29.809 | 8 | <.001 |

**Block 1: Method = Enter**

| **Omnibus Tests of Model Coefficients** |  |  |  |  |
| --- | --- | --- | --- | --- |
|  |  | Chi-square | df | Sig. |
| Step 1 | Step | 34.402 | 8 | <.001 |
|  | Block | 34.402 | 8 | <.001 |
|  | Model | 34.402 | 8 | <.001 |

| **Model Summary** |  |  |  |
| --- | --- | --- | --- |
| Step | -2 Log likelihood | Cox & Snell R Square | Nagelkerke R Square |
| 1 | 103.433^a^ | .225 | .352 |

| a. Estimation terminated at iteration number 6 because parameter estimates changed by less than .001. |  |  |  |
| --- | --- | --- | --- |

| **Hosmer and Lemeshow Test** |  |  |  |
| --- | --- | --- | --- |
| Step | Chi-square | df | Sig. |
| 1 | 5.213 | 8 | .735 |

| **Contingency Table for Hosmer and Lemeshow Test** |  |  |  |  |  |  |
| --- | --- | --- | --- | --- | --- | --- |
|  |  | DFW_FwGe = 0 |  | DFW_FwGe = 1 |  | Total |
|  |  | Observed | Expected | Observed | Expected |  |
| Step 1 | 1 | 16 | 15.826 | 0 | .174 | 16 |
|  | 2 | 11 | 10.806 | 0 | .194 | 11 |
|  | 3 | 8 | 7.694 | 0 | .306 | 8 |
|  | 4 | 14 | 13.336 | 0 | .664 | 14 |
|  | 5 | 11 | 12.339 | 2 | .661 | 13 |
|  | 6 | 7 | 6.931 | 1 | 1.069 | 8 |
|  | 7 | 11 | 12.035 | 4 | 2.965 | 15 |
|  | 8 | 11 | 11.016 | 4 | 3.984 | 15 |
|  | 9 | 9 | 9.428 | 9 | 8.572 | 18 |
|  | 10 | 9 | 7.590 | 8 | 9.410 | 17 |

| **Classification Table**^a^ |  |  |  |  |  |
| --- | --- | --- | --- | --- | --- |
|  | Observed |  | Predicted |  |  |
|  |  |  | DFW_FwGe |  | Percentage Correct |
|  |  |  | 0 | 1 |  |
| Step 1 | DFW_FwGe | 0 | 95 | 12 | 88.8 |
|  |  | 1 | 14 | 14 | 50.0 |
|  | Overall Percentage |  |  |  | 80.7 |

| a. The cut value is .500 |  |  |  |  |  |
| --- | --- | --- | --- | --- | --- |

| **Variables in the Equation** |  |  |  |  |  |  |  |
| --- | --- | --- | --- | --- | --- | --- | --- |
|  |  | B | S.E. | Wald | df | Sig. | Exp(B) |
|  |  |  |  |  |  |  |  |
| Step 1^a^ | Career_stage(1) | 1.266 | .612 | 4.289 | 1 | .038 | 3.548 |
|  | Age_category |  |  | 5.489 | 4 | .241 |  |
|  | Age_category(1) | -1.595 | .714 | 5.000 | 1 | .025 | .203 |
|  | Age_category(2) | -1.371 | .773 | 3.147 | 1 | .076 | .254 |
|  | Age_category(3) | -1.325 | 1.028 | 1.661 | 1 | .198 | .266 |
|  | Age_category(4) | -1.945 | 1.399 | 1.932 | 1 | .164 | .143 |
|  | Gender_ID |  |  | 15.964 | 3 | .001 |  |
|  | Gender_ID(1) | -3.089 | .793 | 15.181 | 1 | <.001 | .046 |
|  | Gender_ID(2) | .620 | 1.037 | .358 | 1 | .550 | 1.859 |
|  | Gender_ID(3) | -.555 | .894 | .385 | 1 | .535 | .574 |
|  | Constant | .194 | .531 | .134 | 1 | .714 | 1.215 |

| **Variables in the Equation** |  |  |  |
| --- | --- | --- | --- |
|  |  | 95% C.I.for EXP(B) |  |
|  |  | Lower | Upper |
| Step 1^a^ | Career_stage(1) | 1.070 | 11.763 |
|  | Age_category |  |  |
|  | Age_category(1) | .050 | .821 |
|  | Age_category(2) | .056 | 1.155 |
|  | Age_category(3) | .035 | 1.994 |
|  | Age_category(4) | .009 | 2.220 |
|  | Gender_ID |  |  |
|  | Gender_ID(1) | .010 | .215 |
|  | Gender_ID(2) | .244 | 14.181 |
|  | Gender_ID(3) | .100 | 3.312 |
|  | Constant |  |  |

|  |  |  |  |  |  |  |  |
| --- | --- | --- | --- | --- | --- | --- | --- |

| a. Variable(s) entered on step 1: Career_stage, Age_category, Gender_ID. |  |  |  |
| --- | --- | --- | --- |
