# Supplementary material for: #Yourpalaeolife: Interrogating the Status of Fieldwork Among Early Career Palaeontology Researchers
Source: Ecol Evol. 2026 Jul 29;16(8):e74032. doi: 10.1002/ece3.74032 (PMC13420382; doi:10.1002/ece3.74032)
Supplement: Supplementary file 2 — Data S2: ece374032‐sup‐0002‐Supinfo2.zip. [file ECE3-16-e74032-s002.zip › M59 BLR_DiscFW_ModexRC.docx]

**Logistic Regression**

| **Notes** |  |  |
| --- | --- | --- |
| Output Created |  | 03-FEB-2026 16:12:16 |
| Comments |  |  |
| Input | Active Dataset | DataSet7 |
|  | Filter | <none> |
|  | Weight | <none> |
|  | Split File | <none> |
|  | N of Rows in Working Data File | 157 |
| Missing Value Handling | Definition of Missing | User-defined missing values are treated as missing |
| Syntax |  | LOGISTIC REGRESSION VARIABLES DFW_FwMo /METHOD=ENTER Career_stage Age_category Gender_ID /CONTRAST (Career_stage)=Indicator(1) /CONTRAST (Age_category)=Indicator(1) /CONTRAST (Gender_ID)=Indicator(1) /PRINT=GOODFIT CI(95) /CRITERIA=PIN(0.05) POUT(0.10) ITERATE(20) CUT(0.5). |
| Resources | Processor Time | 00:00:00.02 |
|  | Elapsed Time | 00:00:00.01 |

| **Warnings** |
| --- |
| Text: Career_stage Command: LOGISTIC REGRESSION This procedure cannot use string variables longer than 8 bytes. The values will be truncated. |
| Text: Age_category Command: LOGISTIC REGRESSION This procedure cannot use string variables longer than 8 bytes. The values will be truncated. |

| **Case Processing Summary** |  |  |  |
| --- | --- | --- | --- |
| Unweighted Cases^a^ |  | N | Percent |
| Selected Cases | Included in Analysis | 135 | 86.0 |
|  | Missing Cases | 22 | 14.0 |
|  | Total | 157 | 100.0 |
| Unselected Cases |  | 0 | .0 |
| Total |  | 157 | 100.0 |

| a. If weight is in effect, see classification table for the total number of cases. |  |  |  |
| --- | --- | --- | --- |

| **Dependent Variable Encoding** |  |
| --- | --- |
| Original Value | Internal Value |
| 0 | 0 |
| 1 | 1 |

| **Categorical Variables Codings** |  |  |  |  |  |  |
| --- | --- | --- | --- | --- | --- | --- |
|  |  | Frequency | Parameter coding |  |  |  |
|  |  |  | (1) | (2) | (3) | (4) |
| Age_category | <25 year | 20 | .000 | .000 | .000 | .000 |
|  | 26-30 ye | 53 | 1.000 | .000 | .000 | .000 |
|  | 31-35 ye | 40 | .000 | 1.000 | .000 | .000 |
|  | 36-40 ye | 15 | .000 | .000 | 1.000 | .000 |
|  | 41+ year | 7 | .000 | .000 | .000 | 1.000 |
| Gender_ID | F | 58 | .000 | .000 | .000 |  |
|  | M | 62 | 1.000 | .000 | .000 |  |
|  | N | 5 | .000 | 1.000 | .000 |  |
|  | U | 10 | .000 | .000 | 1.000 |  |
| Career_stage | PhD cand | 81 | .000 |  |  |  |
|  | Research | 54 | 1.000 |  |  |  |

**Block 0: Beginning Block**

| **Classification Table**^a,b^ |  |  |  |  |  |
| --- | --- | --- | --- | --- | --- |
|  | Observed |  | Predicted |  |  |
|  |  |  | DFW_FwMo |  | Percentage Correct |
|  |  |  | 0 | 1 |  |
| Step 0 | DFW_FwMo | 0 | 129 | 0 | 100.0 |
|  |  | 1 | 6 | 0 | .0 |
|  | Overall Percentage |  |  |  | 95.6 |

| a. Constant is included in the model. |  |  |  |  |  |
| --- | --- | --- | --- | --- | --- |
| b. The cut value is .500 |  |  |  |  |  |

| **Variables in the Equation** |  |  |  |  |  |  |  |
| --- | --- | --- | --- | --- | --- | --- | --- |
|  |  | B | S.E. | Wald | df | Sig. | Exp(B) |
| Step 0 | Constant | -3.068 | .418 | 53.968 | 1 | <.001 | .047 |

| **Variables not in the Equation** |  |  |  |  |  |
| --- | --- | --- | --- | --- | --- |
|  |  |  | Score | df | Sig. |
| Step 0 | Variables | Career_stage(1) | 1.424 | 1 | .233 |
|  |  | Age_category | 9.419 | 4 | .051 |
|  |  | Age_category(1) | 4.058 | 1 | .044 |
|  |  | Age_category(2) | 4.131 | 1 | .042 |
|  |  | Age_category(3) | 3.140 | 1 | .076 |
|  |  | Age_category(4) | .343 | 1 | .558 |
|  |  | Gender_ID | 3.472 | 3 | .324 |
|  |  | Gender_ID(1) | .042 | 1 | .838 |
|  |  | Gender_ID(2) | 2.958 | 1 | .085 |
|  |  | Gender_ID(3) | .502 | 1 | .478 |
|  | Overall Statistics |  | 19.769 | 8 | .011 |

**Block 1: Method = Enter**

| **Omnibus Tests of Model Coefficients** |  |  |  |  |
| --- | --- | --- | --- | --- |
|  |  | Chi-square | df | Sig. |
| Step 1 | Step | 22.088 | 8 | .005 |
|  | Block | 22.088 | 8 | .005 |
|  | Model | 22.088 | 8 | .005 |

| **Model Summary** |  |  |  |
| --- | --- | --- | --- |
| Step | -2 Log likelihood | Cox & Snell R Square | Nagelkerke R Square |
| 1 | 27.003^a^ | .151 | .495 |

| a. Estimation terminated at iteration number 20 because maximum iterations has been reached. Final solution cannot be found. |  |  |  |
| --- | --- | --- | --- |

| **Hosmer and Lemeshow Test** |  |  |  |
| --- | --- | --- | --- |
| Step | Chi-square | df | Sig. |
| 1 | .875 | 8 | .999 |

| **Contingency Table for Hosmer and Lemeshow Test** |  |  |  |  |  |  |
| --- | --- | --- | --- | --- | --- | --- |
|  |  | DFW_FwMo = 0 |  | DFW_FwMo = 1 |  | Total |
|  |  | Observed | Expected | Observed | Expected |  |
| Step 1 | 1 | 13 | 13.000 | 0 | .000 | 13 |
|  | 2 | 8 | 8.000 | 0 | .000 | 8 |
|  | 3 | 15 | 15.000 | 0 | .000 | 15 |
|  | 4 | 12 | 12.000 | 0 | .000 | 12 |
|  | 5 | 15 | 15.000 | 0 | .000 | 15 |
|  | 6 | 15 | 15.000 | 0 | .000 | 15 |
|  | 7 | 13 | 12.878 | 0 | .122 | 13 |
|  | 8 | 14 | 13.664 | 0 | .336 | 14 |
|  | 9 | 10 | 10.458 | 1 | .542 | 11 |
|  | 10 | 14 | 14.000 | 5 | 5.000 | 19 |

| **Classification Table**^a^ |  |  |  |  |  |
| --- | --- | --- | --- | --- | --- |
|  | Observed |  | Predicted |  |  |
|  |  |  | DFW_FwMo |  | Percentage Correct |
|  |  |  | 0 | 1 |  |
| Step 1 | DFW_FwMo | 0 | 129 | 0 | 100.0 |
|  |  | 1 | 5 | 1 | 16.7 |
|  | Overall Percentage |  |  |  | 96.3 |

| a. The cut value is .500 |  |  |  |  |  |
| --- | --- | --- | --- | --- | --- |

| **Variables in the Equation** |  |  |  |  |  |  |
| --- | --- | --- | --- | --- | --- | --- |
|  |  | B | S.E. | Wald | df | Sig. |
|  |  |  |  |  |  |  |
| Step 1^a^ | Career_stage(1) | -2.506 | 1.218 | 4.232 | 1 | .040 |
|  | Age_category |  |  | .741 | 4 | .946 |
|  | Age_category(1) | -.046 | 8571.966 | .000 | 1 | 1.000 |
|  | Age_category(2) | 34.357 | 8353.233 | .000 | 1 | .997 |
|  | Age_category(3) | 35.281 | 8353.233 | .000 | 1 | .997 |
|  | Age_category(4) | 15.826 | 16286.768 | .000 | 1 | .999 |
|  | Gender_ID |  |  | .317 | 3 | .957 |
|  | Gender_ID(1) | .586 | 1.041 | .317 | 1 | .573 |
|  | Gender_ID(2) | 18.328 | 3913.160 | .000 | 1 | .996 |
|  | Gender_ID(3) | -17.328 | 9992.561 | .000 | 1 | .999 |
|  | Constant | -36.141 | 8353.233 | .000 | 1 | .997 |

| **Variables in the Equation** |  |  |  |  |
| --- | --- | --- | --- | --- |
|  |  | Exp(B) | 95% C.I.for EXP(B) |  |
|  |  |  | Lower | Upper |
| Step 1^a^ | Career_stage(1) | .082 | .007 | .888 |
|  | Age_category |  |  |  |
|  | Age_category(1) | .955 | .000 | . |
|  | Age_category(2) | 833993194025806.100 | .000 | . |
|  | Age_category(3) | 2099554083653557.000 | .000 | . |
|  | Age_category(4) | 7469730.646 | .000 | . |
|  | Gender_ID |  |  |  |
|  | Gender_ID(1) | 1.797 | .234 | 13.827 |
|  | Gender_ID(2) | 91156722.533 | .000 | . |
|  | Gender_ID(3) | .000 | .000 | . |
|  | Constant | .000 |  |  |

|  |  |  |  |  |  |  |
| --- | --- | --- | --- | --- | --- | --- |

| a. Variable(s) entered on step 1: Career_stage, Age_category, Gender_ID. |  |  |  |  |
| --- | --- | --- | --- | --- |
