# Supplementary material for: #Yourpalaeolife: Interrogating the Status of Fieldwork Among Early Career Palaeontology Researchers
Source: Ecol Evol. 2026 Jul 29;16(8):e74032. doi: 10.1002/ece3.74032 (PMC13420382; doi:10.1002/ece3.74032)
Supplement: Supplementary file 2 — Data S2: ece374032‐sup‐0002‐Supinfo2.zip. [file ECE3-16-e74032-s002.zip › M61 BLR_DiscFW_NonexRC.docx]

**Logistic Regression**

| **Notes** |  |  |
| --- | --- | --- |
| Output Created |  | 03-FEB-2026 16:15:49 |
| Comments |  |  |
| Input | Active Dataset | DataSet7 |
|  | Filter | <none> |
|  | Weight | <none> |
|  | Split File | <none> |
|  | N of Rows in Working Data File | 157 |
| Missing Value Handling | Definition of Missing | User-defined missing values are treated as missing |
| Syntax |  | LOGISTIC REGRESSION VARIABLES DFW_FwNo /METHOD=ENTER Career_stage Age_category Gender_ID /CONTRAST (Career_stage)=Indicator(1) /CONTRAST (Age_category)=Indicator(1) /CONTRAST (Gender_ID)=Indicator(1) /PRINT=GOODFIT CI(95) /CRITERIA=PIN(0.05) POUT(0.10) ITERATE(20) CUT(0.5). |
| Resources | Processor Time | 00:00:00.00 |
|  | Elapsed Time | 00:00:00.01 |

| **Warnings** |
| --- |
| Text: Career_stage Command: LOGISTIC REGRESSION This procedure cannot use string variables longer than 8 bytes. The values will be truncated. |
| Text: Age_category Command: LOGISTIC REGRESSION This procedure cannot use string variables longer than 8 bytes. The values will be truncated. |

| **Case Processing Summary** |  |  |  |
| --- | --- | --- | --- |
| Unweighted Cases^a^ |  | N | Percent |
| Selected Cases | Included in Analysis | 135 | 86.0 |
|  | Missing Cases | 22 | 14.0 |
|  | Total | 157 | 100.0 |
| Unselected Cases |  | 0 | .0 |
| Total |  | 157 | 100.0 |

| a. If weight is in effect, see classification table for the total number of cases. |  |  |  |
| --- | --- | --- | --- |

| **Dependent Variable Encoding** |  |
| --- | --- |
| Original Value | Internal Value |
| 0 | 0 |
| 1 | 1 |

| **Categorical Variables Codings** |  |  |  |  |  |  |
| --- | --- | --- | --- | --- | --- | --- |
|  |  | Frequency | Parameter coding |  |  |  |
|  |  |  | (1) | (2) | (3) | (4) |
| Age_category | <25 year | 20 | .000 | .000 | .000 | .000 |
|  | 26-30 ye | 53 | 1.000 | .000 | .000 | .000 |
|  | 31-35 ye | 40 | .000 | 1.000 | .000 | .000 |
|  | 36-40 ye | 15 | .000 | .000 | 1.000 | .000 |
|  | 41+ year | 7 | .000 | .000 | .000 | 1.000 |
| Gender_ID | F | 58 | .000 | .000 | .000 |  |
|  | M | 62 | 1.000 | .000 | .000 |  |
|  | N | 5 | .000 | 1.000 | .000 |  |
|  | U | 10 | .000 | .000 | 1.000 |  |
| Career_stage | PhD cand | 81 | .000 |  |  |  |
|  | Research | 54 | 1.000 |  |  |  |

**Block 0: Beginning Block**

| **Classification Table**^a,b^ |  |  |  |  |  |
| --- | --- | --- | --- | --- | --- |
|  | Observed |  | Predicted |  |  |
|  |  |  | DFW_FwNo |  | Percentage Correct |
|  |  |  | 0 | 1 |  |
| Step 0 | DFW_FwNo | 0 | 0 | 52 | .0 |
|  |  | 1 | 0 | 83 | 100.0 |
|  | Overall Percentage |  |  |  | 61.5 |

| a. Constant is included in the model. |  |  |  |  |  |
| --- | --- | --- | --- | --- | --- |
| b. The cut value is .500 |  |  |  |  |  |

| **Variables in the Equation** |  |  |  |  |  |  |  |
| --- | --- | --- | --- | --- | --- | --- | --- |
|  |  | B | S.E. | Wald | df | Sig. | Exp(B) |
| Step 0 | Constant | .468 | .177 | 6.990 | 1 | .008 | 1.596 |

| **Variables not in the Equation** |  |  |  |  |  |
| --- | --- | --- | --- | --- | --- |
|  |  |  | Score | df | Sig. |
| Step 0 | Variables | Career_stage(1) | .631 | 1 | .427 |
|  |  | Age_category | 6.141 | 4 | .189 |
|  |  | Age_category(1) | 5.398 | 1 | .020 |
|  |  | Age_category(2) | 1.008 | 1 | .315 |
|  |  | Age_category(3) | .016 | 1 | .900 |
|  |  | Age_category(4) | 1.081 | 1 | .298 |
|  |  | Gender_ID | 10.627 | 3 | .014 |
|  |  | Gender_ID(1) | 9.935 | 1 | .002 |
|  |  | Gender_ID(2) | 1.012 | 1 | .314 |
|  |  | Gender_ID(3) | .010 | 1 | .920 |
|  | Overall Statistics |  | 18.441 | 8 | .018 |

**Block 1: Method = Enter**

| **Omnibus Tests of Model Coefficients** |  |  |  |  |
| --- | --- | --- | --- | --- |
|  |  | Chi-square | df | Sig. |
| Step 1 | Step | 19.715 | 8 | .011 |
|  | Block | 19.715 | 8 | .011 |
|  | Model | 19.715 | 8 | .011 |

| **Model Summary** |  |  |  |
| --- | --- | --- | --- |
| Step | -2 Log likelihood | Cox & Snell R Square | Nagelkerke R Square |
| 1 | 160.253^a^ | .136 | .185 |

| a. Estimation terminated at iteration number 4 because parameter estimates changed by less than .001. |  |  |  |
| --- | --- | --- | --- |

| **Hosmer and Lemeshow Test** |  |  |  |
| --- | --- | --- | --- |
| Step | Chi-square | df | Sig. |
| 1 | 5.008 | 8 | .757 |

| **Contingency Table for Hosmer and Lemeshow Test** |  |  |  |  |  |  |
| --- | --- | --- | --- | --- | --- | --- |
|  |  | DFW_FwNo = 0 |  | DFW_FwNo = 1 |  | Total |
|  |  | Observed | Expected | Observed | Expected |  |
| Step 1 | 1 | 11 | 9.699 | 3 | 4.301 | 14 |
|  | 2 | 8 | 9.573 | 8 | 6.427 | 16 |
|  | 3 | 5 | 7.312 | 9 | 6.688 | 14 |
|  | 4 | 7 | 5.577 | 5 | 6.423 | 12 |
|  | 5 | 5 | 4.754 | 9 | 9.246 | 14 |
|  | 6 | 5 | 5.090 | 11 | 10.910 | 16 |
|  | 7 | 4 | 3.650 | 8 | 8.350 | 12 |
|  | 8 | 5 | 3.321 | 9 | 10.679 | 14 |
|  | 9 | 1 | 1.425 | 7 | 6.575 | 8 |
|  | 10 | 1 | 1.598 | 14 | 13.402 | 15 |

| **Classification Table**^a^ |  |  |  |  |  |
| --- | --- | --- | --- | --- | --- |
|  | Observed |  | Predicted |  |  |
|  |  |  | DFW_FwNo |  | Percentage Correct |
|  |  |  | 0 | 1 |  |
| Step 1 | DFW_FwNo | 0 | 24 | 28 | 46.2 |
|  |  | 1 | 20 | 63 | 75.9 |
|  | Overall Percentage |  |  |  | 64.4 |

| a. The cut value is .500 |  |  |  |  |  |
| --- | --- | --- | --- | --- | --- |

| **Variables in the Equation** |  |  |  |  |  |  |  |
| --- | --- | --- | --- | --- | --- | --- | --- |
|  |  | B | S.E. | Wald | df | Sig. | Exp(B) |
|  |  |  |  |  |  |  |  |
| Step 1^a^ | Career_stage(1) | -.597 | .460 | 1.689 | 1 | .194 | .550 |
|  | Age_category |  |  | 5.944 | 4 | .203 |  |
|  | Age_category(1) | 1.123 | .600 | 3.503 | 1 | .061 | 3.075 |
|  | Age_category(2) | .259 | .635 | .167 | 1 | .683 | 1.296 |
|  | Age_category(3) | .423 | .802 | .277 | 1 | .598 | 1.526 |
|  | Age_category(4) | -.322 | .989 | .106 | 1 | .744 | .724 |
|  | Gender_ID |  |  | 11.848 | 3 | .008 |  |
|  | Gender_ID(1) | 1.359 | .428 | 10.097 | 1 | .001 | 3.890 |
|  | Gender_ID(2) | -.805 | .996 | .653 | 1 | .419 | .447 |
|  | Gender_ID(3) | .059 | .731 | .006 | 1 | .936 | 1.060 |
|  | Constant | -.355 | .491 | .525 | 1 | .469 | .701 |

| **Variables in the Equation** |  |  |  |
| --- | --- | --- | --- |
|  |  | 95% C.I.for EXP(B) |  |
|  |  | Lower | Upper |
| Step 1^a^ | Career_stage(1) | .223 | 1.355 |
|  | Age_category |  |  |
|  | Age_category(1) | .948 | 9.970 |
|  | Age_category(2) | .374 | 4.496 |
|  | Age_category(3) | .317 | 7.354 |
|  | Age_category(4) | .104 | 5.033 |
|  | Gender_ID |  |  |
|  | Gender_ID(1) | 1.683 | 8.993 |
|  | Gender_ID(2) | .064 | 3.147 |
|  | Gender_ID(3) | .253 | 4.443 |
|  | Constant |  |  |

|  |  |  |  |  |  |  |  |
| --- | --- | --- | --- | --- | --- | --- | --- |

| a. Variable(s) entered on step 1: Career_stage, Age_category, Gender_ID. |  |  |  |
| --- | --- | --- | --- |
