# Supplementary material for: #Yourpalaeolife: Interrogating the Status of Fieldwork Among Early Career Palaeontology Researchers
Source: Ecol Evol. 2026 Jul 29;16(8):e74032. doi: 10.1002/ece3.74032 (PMC13420382; doi:10.1002/ece3.74032)
Supplement: Supplementary file 2 — Data S2: ece374032‐sup‐0002‐Supinfo2.zip. [file ECE3-16-e74032-s002.zip › M67 OLR_RCxEL.docx]

**PLUM - Ordinal Regression**

| **Notes** |  |  |
| --- | --- | --- |
| Output Created |  | 03-FEB-2026 17:07:40 |
| Comments |  |  |
| Input | Active Dataset | DataSet9 |
|  | Filter | <none> |
|  | Weight | <none> |
|  | Split File | <none> |
|  | N of Rows in Working Data File | 157 |
| Missing Value Handling | Definition of Missing | User-defined missing values are treated as missing. |
|  | Cases Used | Statistics are based on all cases with valid data for all variables in the model. |
| Syntax |  | PLUM CEL BY Career_stage Gender_ID Age_category WITH ELNT /CRITERIA=CIN(95) DELTA(0) LCONVERGE(0) MXITER(100) MXSTEP(5) PCONVERGE(1.0E-6) SINGULAR(1.0E-8) /LINK=LOGIT /PRINT=FIT PARAMETER SUMMARY TPARALLEL. |
| Resources | Processor Time | 00:00:00.00 |
|  | Elapsed Time | 00:00:00.01 |

| **Warnings** |
| --- |
| There are 138 (61.3%) cells (i.e., dependent variable levels by observed combinations of predictor variable values) with zero frequencies. |

| **Case Processing Summary** |  |  |  |
| --- | --- | --- | --- |
|  |  | N | Marginal Percentage |
| CEL | 1 | 14 | 9.6% |
|  | 2 | 28 | 19.2% |
|  | 3 | 37 | 25.3% |
|  | 4 | 43 | 29.5% |
|  | 5 | 24 | 16.4% |
| Career_stage | PhD candidate | 80 | 54.8% |
|  | Researcher in palaeontology up to 5 years post-PhD | 66 | 45.2% |
| Gender_ID | F | 63 | 43.2% |
|  | M | 64 | 43.8% |
|  | N | 6 | 4.1% |
|  | U | 13 | 8.9% |
| Age_category | <25 years old | 17 | 11.6% |
|  | 26-30 years old | 56 | 38.4% |
|  | 31-35 years old | 50 | 34.2% |
|  | 36-40 years old | 17 | 11.6% |
|  | 41+ years old | 6 | 4.1% |
| Valid |  | 146 | 100.0% |
| Missing |  | 11 |  |
| Total |  | 157 |  |

| **Model Fitting Information** |  |  |  |  |
| --- | --- | --- | --- | --- |
| Model | -2 Log Likelihood | Chi-Square | df | Sig. |
| Intercept Only | 296.055 |  |  |  |
| Final | 215.327 | 80.727 | 9 | <.001 |

| Link function: Logit. |  |  |  |  |
| --- | --- | --- | --- | --- |

| **Goodness-of-Fit** |  |  |  |
| --- | --- | --- | --- |
|  | Chi-Square | df | Sig. |
| Pearson | 168.329 | 167 | .457 |
| Deviance | 137.446 | 167 | .954 |

| Link function: Logit. |  |  |  |
| --- | --- | --- | --- |

| **Pseudo R-Square** |  |
| --- | --- |
| Cox and Snell | .425 |
| Nagelkerke | .445 |
| McFadden | .179 |

| Link function: Logit. |  |
| --- | --- |

| **Parameter Estimates** |  |  |  |  |  |  |
| --- | --- | --- | --- | --- | --- | --- |
|  |  | Estimate | Std. Error | Wald | df | Sig. |
|  |  |  |  |  |  |  |
| Threshold | [CEL = 1] | -6.711 | 1.118 | 36.057 | 1 | <.001 |
|  | [CEL = 2] | -4.925 | 1.060 | 21.595 | 1 | <.001 |
|  | [CEL = 3] | -3.271 | 1.011 | 10.473 | 1 | .001 |
|  | [CEL = 4] | -1.221 | .982 | 1.548 | 1 | .213 |
| Location | ELNT | -2.821 | .390 | 52.434 | 1 | <.001 |
|  | [Career_stage=PhD candidate] | -.690 | .361 | 3.648 | 1 | .056 |
|  | [Career_stage=Researcher in palaeontology up to 5 years post-PhD] | 0^a^ | . | . | 0 | . |
|  | [Gender_ID=F] | -.713 | .574 | 1.545 | 1 | .214 |
|  | [Gender_ID=M] | -.094 | .577 | .026 | 1 | .871 |
|  | [Gender_ID=N] | -1.005 | .915 | 1.205 | 1 | .272 |
|  | [Gender_ID=U] | 0^a^ | . | . | 0 | . |
|  | [Age_category=<25 years old] | -1.745 | .938 | 3.465 | 1 | .063 |
|  | [Age_category=26-30 years old] | -2.158 | .841 | 6.575 | 1 | .010 |
|  | [Age_category=31-35 years old] | -1.478 | .827 | 3.196 | 1 | .074 |
|  | [Age_category=36-40 years old] | -.773 | .908 | .724 | 1 | .395 |
|  | [Age_category=41+ years old] | 0^a^ | . | . | 0 | . |

| **Parameter Estimates** |  |  |  |
| --- | --- | --- | --- |
|  |  | 95% Confidence Interval |  |
|  |  | Lower Bound | Upper Bound |
| Threshold | [CEL = 1] | -8.901 | -4.520 |
|  | [CEL = 2] | -7.003 | -2.848 |
|  | [CEL = 3] | -5.252 | -1.290 |
|  | [CEL = 4] | -3.145 | .702 |
| Location | ELNT | -3.584 | -2.057 |
|  | [Career_stage=PhD candidate] | -1.397 | .018 |
|  | [Career_stage=Researcher in palaeontology up to 5 years post-PhD] | . | . |
|  | [Gender_ID=F] | -1.837 | .411 |
|  | [Gender_ID=M] | -1.225 | 1.038 |
|  | [Gender_ID=N] | -2.798 | .789 |
|  | [Gender_ID=U] | . | . |
|  | [Age_category=<25 years old] | -3.583 | .092 |
|  | [Age_category=26-30 years old] | -3.807 | -.508 |
|  | [Age_category=31-35 years old] | -3.099 | .142 |
|  | [Age_category=36-40 years old] | -2.553 | 1.007 |
|  | [Age_category=41+ years old] | . | . |

|  |  |  |  |  |  |  |
| --- | --- | --- | --- | --- | --- | --- |
|  |  |  |  |  |  |  |

| Link function: Logit. |  |  |  |
| --- | --- | --- | --- |
| a. This parameter is set to zero because it is redundant. |  |  |  |

| **Test of Parallel Lines**^a^ |  |  |  |  |
| --- | --- | --- | --- | --- |
| Model | -2 Log Likelihood | Chi-Square | df | Sig. |
| Null Hypothesis | 215.327 |  |  |  |
| General | 184.675^b^ | 30.652^c^ | 27 | .286 |

| The null hypothesis states that the location parameters (slope coefficients) are the same across response categories.^a^ |  |  |  |  |
| --- | --- | --- | --- | --- |
| a. Link function: Logit. |  |  |  |  |
| b. The log-likelihood value cannot be further increased after maximum number of step-halving. |  |  |  |  |
| c. The Chi-Square statistic is computed based on the log-likelihood value of the last iteration of the general model. Validity of the test is uncertain. |  |  |  |  |
