# Supplementary material for: #Yourpalaeolife: Interrogating the Status of Fieldwork Among Early Career Palaeontology Researchers
Source: Ecol Evol. 2026 Jul 29;16(8):e74032. doi: 10.1002/ece3.74032 (PMC13420382; doi:10.1002/ece3.74032)
Supplement: Supplementary file 2 — Data S2: ece374032‐sup‐0002‐Supinfo2.zip. [file ECE3-16-e74032-s002.zip › M69 OLR_RCxFEX.docx]

**PLUM - Ordinal Regression**

| **Notes** |  |  |
| --- | --- | --- |
| Output Created |  | 03-FEB-2026 16:56:53 |
| Comments |  |  |
| Input | Active Dataset | DataSet9 |
|  | Filter | <none> |
|  | Weight | <none> |
|  | Split File | <none> |
|  | N of Rows in Working Data File | 157 |
| Missing Value Handling | Definition of Missing | User-defined missing values are treated as missing. |
|  | Cases Used | Statistics are based on all cases with valid data for all variables in the model. |
| Syntax |  | PLUM CFEX BY Career_stage Gender_ID Age_category WITH FENT /CRITERIA=CIN(95) DELTA(0) LCONVERGE(0) MXITER(100) MXSTEP(5) PCONVERGE(1.0E-6) SINGULAR(1.0E-8) /LINK=LOGIT /PRINT=FIT PARAMETER SUMMARY TPARALLEL. |
| Resources | Processor Time | 00:00:00.00 |
|  | Elapsed Time | 00:00:00.01 |

| **Warnings** |
| --- |
| There are 123 (63.1%) cells (i.e., dependent variable levels by observed combinations of predictor variable values) with zero frequencies. |

| **Case Processing Summary** |  |  |  |
| --- | --- | --- | --- |
|  |  | N | Marginal Percentage |
| CFEX | 1 | 4 | 2.6% |
|  | 2 | 15 | 9.9% |
|  | 3 | 18 | 11.9% |
|  | 4 | 64 | 42.4% |
|  | 5 | 50 | 33.1% |
| Career_stage | PhD candidate | 84 | 55.6% |
|  | Researcher in palaeontology up to 5 years post-PhD | 67 | 44.4% |
| Gender_ID | F | 66 | 43.7% |
|  | M | 67 | 44.4% |
|  | N | 5 | 3.3% |
|  | U | 13 | 8.6% |
| Age_category | <25 years old | 19 | 12.6% |
|  | 26-30 years old | 57 | 37.7% |
|  | 31-35 years old | 50 | 33.1% |
|  | 36-40 years old | 18 | 11.9% |
|  | 41+ years old | 7 | 4.6% |
| Valid |  | 151 | 100.0% |
| Missing |  | 6 |  |
| Total |  | 157 |  |

| **Model Fitting Information** |  |  |  |  |
| --- | --- | --- | --- | --- |
| Model | -2 Log Likelihood | Chi-Square | df | Sig. |
| Intercept Only | 233.087 |  |  |  |
| Final | 183.158 | 49.929 | 9 | <.001 |

| Link function: Logit. |  |  |  |  |
| --- | --- | --- | --- | --- |

| **Goodness-of-Fit** |  |  |  |
| --- | --- | --- | --- |
|  | Chi-Square | df | Sig. |
| Pearson | 232.318 | 143 | <.001 |
| Deviance | 118.614 | 143 | .932 |

| Link function: Logit. |  |  |  |
| --- | --- | --- | --- |

| **Pseudo R-Square** |  |
| --- | --- |
| Cox and Snell | .282 |
| Nagelkerke | .304 |
| McFadden | .126 |

| Link function: Logit. |  |
| --- | --- |

| **Parameter Estimates** |  |  |  |  |  |  |
| --- | --- | --- | --- | --- | --- | --- |
|  |  | Estimate | Std. Error | Wald | df | Sig. |
|  |  |  |  |  |  |  |
| Threshold | [CFEX = 1] | -5.005 | 1.115 | 20.165 | 1 | <.001 |
|  | [CFEX = 2] | -3.064 | .996 | 9.470 | 1 | .002 |
|  | [CFEX = 3] | -2.016 | .970 | 4.316 | 1 | .038 |
|  | [CFEX = 4] | .305 | .953 | .102 | 1 | .749 |
| Location | FENT | -2.464 | .479 | 26.511 | 1 | <.001 |
|  | [Career_stage=PhD candidate] | .086 | .364 | .055 | 1 | .814 |
|  | [Career_stage=Researcher in palaeontology up to 5 years post-PhD] | 0^a^ | . | . | 0 | . |
|  | [Gender_ID=F] | -.640 | .584 | 1.201 | 1 | .273 |
|  | [Gender_ID=M] | .600 | .590 | 1.036 | 1 | .309 |
|  | [Gender_ID=N] | .823 | 1.024 | .647 | 1 | .421 |
|  | [Gender_ID=U] | 0^a^ | . | . | 0 | . |
|  | [Age_category=<25 years old] | -.548 | .883 | .385 | 1 | .535 |
|  | [Age_category=26-30 years old] | -.392 | .793 | .244 | 1 | .621 |
|  | [Age_category=31-35 years old] | -.579 | .788 | .540 | 1 | .462 |
|  | [Age_category=36-40 years old] | .820 | .898 | .834 | 1 | .361 |
|  | [Age_category=41+ years old] | 0^a^ | . | . | 0 | . |

| **Parameter Estimates** |  |  |  |
| --- | --- | --- | --- |
|  |  | 95% Confidence Interval |  |
|  |  | Lower Bound | Upper Bound |
| Threshold | [CFEX = 1] | -7.189 | -2.820 |
|  | [CFEX = 2] | -5.015 | -1.112 |
|  | [CFEX = 3] | -3.917 | -.114 |
|  | [CFEX = 4] | -1.563 | 2.172 |
| Location | FENT | -3.402 | -1.526 |
|  | [Career_stage=PhD candidate] | -.628 | .799 |
|  | [Career_stage=Researcher in palaeontology up to 5 years post-PhD] | . | . |
|  | [Gender_ID=F] | -1.785 | .505 |
|  | [Gender_ID=M] | -.556 | 1.756 |
|  | [Gender_ID=N] | -1.183 | 2.830 |
|  | [Gender_ID=U] | . | . |
|  | [Age_category=<25 years old] | -2.278 | 1.182 |
|  | [Age_category=26-30 years old] | -1.946 | 1.162 |
|  | [Age_category=31-35 years old] | -2.124 | .966 |
|  | [Age_category=36-40 years old] | -.940 | 2.581 |
|  | [Age_category=41+ years old] | . | . |

|  |  |  |  |  |  |  |
| --- | --- | --- | --- | --- | --- | --- |
|  |  |  |  |  |  |  |

| Link function: Logit. |  |  |  |
| --- | --- | --- | --- |
| a. This parameter is set to zero because it is redundant. |  |  |  |

| **Test of Parallel Lines**^a^ |  |  |  |  |
| --- | --- | --- | --- | --- |
| Model | -2 Log Likelihood | Chi-Square | df | Sig. |
| Null Hypothesis | 183.158 |  |  |  |
| General | 146.208^b^ | 36.950^c^ | 27 | .096 |

| The null hypothesis states that the location parameters (slope coefficients) are the same across response categories.^a^ |  |  |  |  |
| --- | --- | --- | --- | --- |
| a. Link function: Logit. |  |  |  |  |
| b. The log-likelihood value cannot be further increased after maximum number of step-halving. |  |  |  |  |
| c. The Chi-Square statistic is computed based on the log-likelihood value of the last iteration of the general model. Validity of the test is uncertain. |  |  |  |  |
