# Supplementary material for: #Yourpalaeolife: Interrogating the Status of Fieldwork Among Early Career Palaeontology Researchers
Source: Ecol Evol. 2026 Jul 29;16(8):e74032. doi: 10.1002/ece3.74032 (PMC13420382; doi:10.1002/ece3.74032)
Supplement: Supplementary file 2 — Data S2: ece374032‐sup‐0002‐Supinfo2.zip. [file ECE3-16-e74032-s002.zip › M71 OLR_RCxFID.docx]

**PLUM - Ordinal Regression**

| **Notes** |  |  |
| --- | --- | --- |
| Output Created |  | 03-FEB-2026 16:47:55 |
| Comments |  |  |
| Input | Active Dataset | DataSet9 |
|  | Filter | <none> |
|  | Weight | <none> |
|  | Split File | <none> |
|  | N of Rows in Working Data File | 157 |
| Missing Value Handling | Definition of Missing | User-defined missing values are treated as missing. |
|  | Cases Used | Statistics are based on all cases with valid data for all variables in the model. |
| Syntax |  | PLUM CFID BY Career_stage Gender_ID Age_category WITH FINT /CRITERIA=CIN(95) DELTA(0) LCONVERGE(0) MXITER(100) MXSTEP(5) PCONVERGE(1.0E-6) SINGULAR(1.0E-8) /LINK=LOGIT /PRINT=FIT PARAMETER SUMMARY TPARALLEL. |
| Resources | Processor Time | 00:00:00.00 |
|  | Elapsed Time | 00:00:00.03 |

[DataSet9]

| **Warnings** |
| --- |
| There are 111 (58.4%) cells (i.e., dependent variable levels by observed combinations of predictor variable values) with zero frequencies. |

| **Case Processing Summary** |  |  |  |
| --- | --- | --- | --- |
|  |  | N | Marginal Percentage |
| CFID | 1 | 6 | 4.0% |
|  | 2 | 16 | 10.7% |
|  | 3 | 30 | 20.1% |
|  | 4 | 59 | 39.6% |
|  | 5 | 38 | 25.5% |
| Career_stage | PhD candidate | 81 | 54.4% |
|  | Researcher in palaeontology up to 5 years post-PhD | 68 | 45.6% |
| Gender_ID | F | 62 | 41.6% |
|  | M | 68 | 45.6% |
|  | N | 6 | 4.0% |
|  | U | 13 | 8.7% |
| Age_category | <25 years old | 18 | 12.1% |
|  | 26-30 years old | 56 | 37.6% |
|  | 31-35 years old | 50 | 33.6% |
|  | 36-40 years old | 18 | 12.1% |
|  | 41+ years old | 7 | 4.7% |
| Valid |  | 149 | 100.0% |
| Missing |  | 8 |  |
| Total |  | 157 |  |

| **Model Fitting Information** |  |  |  |  |
| --- | --- | --- | --- | --- |
| Model | -2 Log Likelihood | Chi-Square | df | Sig. |
| Intercept Only | 217.814 |  |  |  |
| Final | 185.771 | 32.043 | 9 | <.001 |

| Link function: Logit. |  |  |  |  |
| --- | --- | --- | --- | --- |

| **Goodness-of-Fit** |  |  |  |
| --- | --- | --- | --- |
|  | Chi-Square | df | Sig. |
| Pearson | 118.531 | 139 | .895 |
| Deviance | 104.986 | 139 | .986 |

| Link function: Logit. |  |  |  |
| --- | --- | --- | --- |

| **Pseudo R-Square** |  |
| --- | --- |
| Cox and Snell | .194 |
| Nagelkerke | .206 |
| McFadden | .076 |

| Link function: Logit. |  |
| --- | --- |

| **Parameter Estimates** |  |  |  |  |  |  |
| --- | --- | --- | --- | --- | --- | --- |
|  |  | Estimate | Std. Error | Wald | df | Sig. |
|  |  |  |  |  |  |  |
| Threshold | [CFID = 1] | -4.729 | 1.026 | 21.230 | 1 | <.001 |
|  | [CFID = 2] | -3.144 | .951 | 10.929 | 1 | <.001 |
|  | [CFID = 3] | -1.850 | .926 | 3.991 | 1 | .046 |
|  | [CFID = 4] | .082 | .911 | .008 | 1 | .928 |
| Location | FINT | -2.448 | .569 | 18.523 | 1 | <.001 |
|  | [Career_stage=PhD candidate] | -.108 | .356 | .093 | 1 | .761 |
|  | [Career_stage=Researcher in palaeontology up to 5 years post-PhD] | 0^a^ | . | . | 0 | . |
|  | [Gender_ID=F] | -.849 | .577 | 2.167 | 1 | .141 |
|  | [Gender_ID=M] | -.116 | .575 | .041 | 1 | .840 |
|  | [Gender_ID=N] | -.549 | .910 | .364 | 1 | .546 |
|  | [Gender_ID=U] | 0^a^ | . | . | 0 | . |
|  | [Age_category=<25 years old] | -.795 | .868 | .838 | 1 | .360 |
|  | [Age_category=26-30 years old] | -.551 | .764 | .519 | 1 | .471 |
|  | [Age_category=31-35 years old] | -.601 | .758 | .630 | 1 | .427 |
|  | [Age_category=36-40 years old] | .634 | .844 | .564 | 1 | .452 |
|  | [Age_category=41+ years old] | 0^a^ | . | . | 0 | . |

| **Parameter Estimates** |  |  |  |
| --- | --- | --- | --- |
|  |  | 95% Confidence Interval |  |
|  |  | Lower Bound | Upper Bound |
| Threshold | [CFID = 1] | -6.740 | -2.717 |
|  | [CFID = 2] | -5.009 | -1.280 |
|  | [CFID = 3] | -3.666 | -.035 |
|  | [CFID = 4] | -1.703 | 1.867 |
| Location | FINT | -3.563 | -1.333 |
|  | [Career_stage=PhD candidate] | -.806 | .589 |
|  | [Career_stage=Researcher in palaeontology up to 5 years post-PhD] | . | . |
|  | [Gender_ID=F] | -1.979 | .281 |
|  | [Gender_ID=M] | -1.243 | 1.011 |
|  | [Gender_ID=N] | -2.333 | 1.234 |
|  | [Gender_ID=U] | . | . |
|  | [Age_category=<25 years old] | -2.496 | .907 |
|  | [Age_category=26-30 years old] | -2.049 | .948 |
|  | [Age_category=31-35 years old] | -2.087 | .884 |
|  | [Age_category=36-40 years old] | -1.020 | 2.289 |
|  | [Age_category=41+ years old] | . | . |

|  |  |  |  |  |  |  |
| --- | --- | --- | --- | --- | --- | --- |
|  |  |  |  |  |  |  |

| Link function: Logit. |  |  |  |
| --- | --- | --- | --- |
| a. This parameter is set to zero because it is redundant. |  |  |  |

| **Test of Parallel Lines**^a^ |  |  |  |  |
| --- | --- | --- | --- | --- |
| Model | -2 Log Likelihood | Chi-Square | df | Sig. |
| Null Hypothesis | 185.771 |  |  |  |
| General | 130.831^b^ | 54.940^c^ | 27 | .001 |

| The null hypothesis states that the location parameters (slope coefficients) are the same across response categories.^a^ |  |  |  |  |
| --- | --- | --- | --- | --- |
| a. Link function: Logit. |  |  |  |  |
| b. The log-likelihood value cannot be further increased after maximum number of step-halving. |  |  |  |  |
| c. The Chi-Square statistic is computed based on the log-likelihood value of the last iteration of the general model. Validity of the test is uncertain. |  |  |  |  |
