# Supplementary material for: #Yourpalaeolife: Interrogating the Status of Fieldwork Among Early Career Palaeontology Researchers
Source: Ecol Evol. 2026 Jul 29;16(8):e74032. doi: 10.1002/ece3.74032 (PMC13420382; doi:10.1002/ece3.74032)
Supplement: Supplementary file 2 — Data S2: ece374032‐sup‐0002‐Supinfo2.zip. [file ECE3-16-e74032-s002.zip › M73 OLR_RCxFJ.docx]

**PLUM - Ordinal Regression**

| **Notes** |  |  |
| --- | --- | --- |
| Output Created |  | 03-FEB-2026 16:59:30 |
| Comments |  |  |
| Input | Active Dataset | DataSet9 |
|  | Filter | <none> |
|  | Weight | <none> |
|  | Split File | <none> |
|  | N of Rows in Working Data File | 157 |
| Missing Value Handling | Definition of Missing | User-defined missing values are treated as missing. |
|  | Cases Used | Statistics are based on all cases with valid data for all variables in the model. |
| Syntax |  | PLUM CFJ BY Career_stage Gender_ID Age_category WITH FJNT /CRITERIA=CIN(95) DELTA(0) LCONVERGE(0) MXITER(100) MXSTEP(5) PCONVERGE(1.0E-6) SINGULAR(1.0E-8) /LINK=LOGIT /PRINT=FIT PARAMETER SUMMARY TPARALLEL. |
| Resources | Processor Time | 00:00:00.00 |
|  | Elapsed Time | 00:00:00.01 |

| **Warnings** |
| --- |
| There are 134 (62.3%) cells (i.e., dependent variable levels by observed combinations of predictor variable values) with zero frequencies. |

| **Case Processing Summary** |  |  |  |
| --- | --- | --- | --- |
|  |  | N | Marginal Percentage |
| CFJ | 1 | 35 | 24.0% |
|  | 2 | 19 | 13.0% |
|  | 3 | 18 | 12.3% |
|  | 4 | 39 | 26.7% |
|  | 5 | 35 | 24.0% |
| Career_stage | PhD candidate | 80 | 54.8% |
|  | Researcher in palaeontology up to 5 years post-PhD | 66 | 45.2% |
| Gender_ID | F | 62 | 42.5% |
|  | M | 66 | 45.2% |
|  | N | 5 | 3.4% |
|  | U | 13 | 8.9% |
| Age_category | <25 years old | 16 | 11.0% |
|  | 26-30 years old | 57 | 39.0% |
|  | 31-35 years old | 48 | 32.9% |
|  | 36-40 years old | 18 | 12.3% |
|  | 41+ years old | 7 | 4.8% |
| Valid |  | 146 | 100.0% |
| Missing |  | 11 |  |
| Total |  | 157 |  |

| **Model Fitting Information** |  |  |  |  |
| --- | --- | --- | --- | --- |
| Model | -2 Log Likelihood | Chi-Square | df | Sig. |
| Intercept Only | 302.903 |  |  |  |
| Final | 191.907 | 110.995 | 9 | <.001 |

| Link function: Logit. |  |  |  |  |
| --- | --- | --- | --- | --- |

| **Goodness-of-Fit** |  |  |  |
| --- | --- | --- | --- |
|  | Chi-Square | df | Sig. |
| Pearson | 167.978 | 159 | .298 |
| Deviance | 119.964 | 159 | .991 |

| Link function: Logit. |  |  |  |
| --- | --- | --- | --- |

| **Pseudo R-Square** |  |
| --- | --- |
| Cox and Snell | .532 |
| Nagelkerke | .557 |
| McFadden | .244 |

| Link function: Logit. |  |
| --- | --- |

| **Parameter Estimates** |  |  |  |  |  |  |
| --- | --- | --- | --- | --- | --- | --- |
|  |  | Estimate | Std. Error | Wald | df | Sig. |
|  |  |  |  |  |  |  |
| Threshold | [CFJ = 1] | -4.013 | 1.026 | 15.286 | 1 | <.001 |
|  | [CFJ = 2] | -2.705 | .989 | 7.473 | 1 | .006 |
|  | [CFJ = 3] | -1.590 | .964 | 2.722 | 1 | .099 |
|  | [CFJ = 4] | .225 | .953 | .056 | 1 | .813 |
| Location | FJNT | -3.882 | .464 | 69.874 | 1 | <.001 |
|  | [Career_stage=PhD candidate] | -.113 | .369 | .094 | 1 | .759 |
|  | [Career_stage=Researcher in palaeontology up to 5 years post-PhD] | 0^a^ | . | . | 0 | . |
|  | [Gender_ID=F] | -.301 | .593 | .258 | 1 | .611 |
|  | [Gender_ID=M] | .720 | .591 | 1.483 | 1 | .223 |
|  | [Gender_ID=N] | 1.787 | 1.041 | 2.946 | 1 | .086 |
|  | [Gender_ID=U] | 0^a^ | . | . | 0 | . |
|  | [Age_category=<25 years old] | -.805 | .894 | .809 | 1 | .368 |
|  | [Age_category=26-30 years old] | -.733 | .782 | .877 | 1 | .349 |
|  | [Age_category=31-35 years old] | -.507 | .776 | .426 | 1 | .514 |
|  | [Age_category=36-40 years old] | -.399 | .858 | .216 | 1 | .642 |
|  | [Age_category=41+ years old] | 0^a^ | . | . | 0 | . |

| **Parameter Estimates** |  |  |  |
| --- | --- | --- | --- |
|  |  | 95% Confidence Interval |  |
|  |  | Lower Bound | Upper Bound |
| Threshold | [CFJ = 1] | -6.024 | -2.001 |
|  | [CFJ = 2] | -4.644 | -.766 |
|  | [CFJ = 3] | -3.479 | .299 |
|  | [CFJ = 4] | -1.642 | 2.092 |
| Location | FJNT | -4.793 | -2.972 |
|  | [Career_stage=PhD candidate] | -.837 | .611 |
|  | [Career_stage=Researcher in palaeontology up to 5 years post-PhD] | . | . |
|  | [Gender_ID=F] | -1.464 | .861 |
|  | [Gender_ID=M] | -.439 | 1.879 |
|  | [Gender_ID=N] | -.254 | 3.827 |
|  | [Gender_ID=U] | . | . |
|  | [Age_category=<25 years old] | -2.557 | .948 |
|  | [Age_category=26-30 years old] | -2.266 | .800 |
|  | [Age_category=31-35 years old] | -2.028 | 1.015 |
|  | [Age_category=36-40 years old] | -2.080 | 1.282 |
|  | [Age_category=41+ years old] | . | . |

|  |  |  |  |  |  |  |
| --- | --- | --- | --- | --- | --- | --- |
|  |  |  |  |  |  |  |

| Link function: Logit. |  |  |  |
| --- | --- | --- | --- |
| a. This parameter is set to zero because it is redundant. |  |  |  |

| **Test of Parallel Lines**^a^ |  |  |  |  |
| --- | --- | --- | --- | --- |
| Model | -2 Log Likelihood | Chi-Square | df | Sig. |
| Null Hypothesis | 191.907 |  |  |  |
| General | 55.735^b^ | 136.172^c^ | 27 | <.001 |

| The null hypothesis states that the location parameters (slope coefficients) are the same across response categories.^a^ |  |  |  |  |
| --- | --- | --- | --- | --- |
| a. Link function: Logit. |  |  |  |  |
| b. The log-likelihood value cannot be further increased after maximum number of step-halving. |  |  |  |  |
| c. The Chi-Square statistic is computed based on the log-likelihood value of the last iteration of the general model. Validity of the test is uncertain. |  |  |  |  |
