# Supplementary material for: #Yourpalaeolife: Interrogating the Status of Fieldwork Among Early Career Palaeontology Researchers
Source: Ecol Evol. 2026 Jul 29;16(8):e74032. doi: 10.1002/ece3.74032 (PMC13420382; doi:10.1002/ece3.74032)
Supplement: Supplementary file 2 — Data S2: ece374032‐sup‐0002‐Supinfo2.zip. [file ECE3-16-e74032-s002.zip › M75 OLR_RCxFTM.docx]

**PLUM - Ordinal Regression**

| **Notes** |  |  |
| --- | --- | --- |
| Output Created |  | 03-FEB-2026 17:06:48 |
| Comments |  |  |
| Input | Active Dataset | DataSet9 |
|  | Filter | <none> |
|  | Weight | <none> |
|  | Split File | <none> |
|  | N of Rows in Working Data File | 157 |
| Missing Value Handling | Definition of Missing | User-defined missing values are treated as missing. |
|  | Cases Used | Statistics are based on all cases with valid data for all variables in the model. |
| Syntax |  | PLUM CFTM BY Career_stage Gender_ID Age_category WITH FTNT /CRITERIA=CIN(95) DELTA(0) LCONVERGE(0) MXITER(100) MXSTEP(5) PCONVERGE(1.0E-6) SINGULAR(1.0E-8) /LINK=LOGIT /PRINT=FIT PARAMETER SUMMARY TPARALLEL. |
| Resources | Processor Time | 00:00:00.02 |
|  | Elapsed Time | 00:00:00.01 |

| **Warnings** |
| --- |
| There are 134 (60.9%) cells (i.e., dependent variable levels by observed combinations of predictor variable values) with zero frequencies. |

| **Case Processing Summary** |  |  |  |
| --- | --- | --- | --- |
|  |  | N | Marginal Percentage |
| CFTM | 1 | 22 | 15.4% |
|  | 2 | 23 | 16.1% |
|  | 3 | 25 | 17.5% |
|  | 4 | 45 | 31.5% |
|  | 5 | 28 | 19.6% |
| Career_stage | PhD candidate | 79 | 55.2% |
|  | Researcher in palaeontology up to 5 years post-PhD | 64 | 44.8% |
| Gender_ID | F | 60 | 42.0% |
|  | M | 64 | 44.8% |
|  | N | 6 | 4.2% |
|  | U | 13 | 9.1% |
| Age_category | <25 years old | 16 | 11.2% |
|  | 26-30 years old | 56 | 39.2% |
|  | 31-35 years old | 48 | 33.6% |
|  | 36-40 years old | 17 | 11.9% |
|  | 41+ years old | 6 | 4.2% |
| Valid |  | 143 | 100.0% |
| Missing |  | 14 |  |
| Total |  | 157 |  |

| **Model Fitting Information** |  |  |  |  |
| --- | --- | --- | --- | --- |
| Model | -2 Log Likelihood | Chi-Square | df | Sig. |
| Intercept Only | 296.913 |  |  |  |
| Final | 237.220 | 59.694 | 9 | <.001 |

| Link function: Logit. |  |  |  |  |
| --- | --- | --- | --- | --- |

| **Goodness-of-Fit** |  |  |  |
| --- | --- | --- | --- |
|  | Chi-Square | df | Sig. |
| Pearson | 179.707 | 163 | .176 |
| Deviance | 160.131 | 163 | .549 |

| Link function: Logit. |  |  |  |
| --- | --- | --- | --- |

| **Pseudo R-Square** |  |
| --- | --- |
| Cox and Snell | .341 |
| Nagelkerke | .357 |
| McFadden | .133 |

| Link function: Logit. |  |
| --- | --- |

| **Parameter Estimates** |  |  |  |  |  |  |
| --- | --- | --- | --- | --- | --- | --- |
|  |  | Estimate | Std. Error | Wald | df | Sig. |
|  |  |  |  |  |  |  |
| Threshold | [CFTM = 1] | -4.133 | 1.027 | 16.197 | 1 | <.001 |
|  | [CFTM = 2] | -3.002 | 1.007 | 8.892 | 1 | .003 |
|  | [CFTM = 3] | -2.000 | .991 | 4.076 | 1 | .044 |
|  | [CFTM = 4] | -.020 | .971 | .000 | 1 | .984 |
| Location | FTNT | -2.063 | .358 | 33.196 | 1 | <.001 |
|  | [Career_stage=PhD candidate] | .010 | .356 | .001 | 1 | .977 |
|  | [Career_stage=Researcher in palaeontology up to 5 years post-PhD] | 0^a^ | . | . | 0 | . |
|  | [Gender_ID=F] | .147 | .573 | .066 | 1 | .798 |
|  | [Gender_ID=M] | .589 | .573 | 1.059 | 1 | .303 |
|  | [Gender_ID=N] | -.391 | .902 | .188 | 1 | .664 |
|  | [Gender_ID=U] | 0^a^ | . | . | 0 | . |
|  | [Age_category=<25 years old] | -1.942 | .951 | 4.166 | 1 | .041 |
|  | [Age_category=26-30 years old] | -1.390 | .846 | 2.697 | 1 | .101 |
|  | [Age_category=31-35 years old] | -1.097 | .833 | 1.734 | 1 | .188 |
|  | [Age_category=36-40 years old] | .192 | .919 | .044 | 1 | .835 |
|  | [Age_category=41+ years old] | 0^a^ | . | . | 0 | . |

| **Parameter Estimates** |  |  |  |
| --- | --- | --- | --- |
|  |  | 95% Confidence Interval |  |
|  |  | Lower Bound | Upper Bound |
| Threshold | [CFTM = 1] | -6.145 | -2.120 |
|  | [CFTM = 2] | -4.975 | -1.029 |
|  | [CFTM = 3] | -3.941 | -.058 |
|  | [CFTM = 4] | -1.924 | 1.884 |
| Location | FTNT | -2.765 | -1.361 |
|  | [Career_stage=PhD candidate] | -.687 | .707 |
|  | [Career_stage=Researcher in palaeontology up to 5 years post-PhD] | . | . |
|  | [Gender_ID=F] | -.977 | 1.270 |
|  | [Gender_ID=M] | -.533 | 1.712 |
|  | [Gender_ID=N] | -2.159 | 1.377 |
|  | [Gender_ID=U] | . | . |
|  | [Age_category=<25 years old] | -3.806 | -.077 |
|  | [Age_category=26-30 years old] | -3.048 | .269 |
|  | [Age_category=31-35 years old] | -2.730 | .536 |
|  | [Age_category=36-40 years old] | -1.610 | 1.994 |
|  | [Age_category=41+ years old] | . | . |

|  |  |  |  |  |  |  |
| --- | --- | --- | --- | --- | --- | --- |
|  |  |  |  |  |  |  |

| Link function: Logit. |  |  |  |
| --- | --- | --- | --- |
| a. This parameter is set to zero because it is redundant. |  |  |  |

| **Test of Parallel Lines**^a^ |  |  |  |  |
| --- | --- | --- | --- | --- |
| Model | -2 Log Likelihood | Chi-Square | df | Sig. |
| Null Hypothesis | 237.220 |  |  |  |
| General | 205.783^b^ | 31.437^c^ | 27 | .254 |

| The null hypothesis states that the location parameters (slope coefficients) are the same across response categories.^a^ |  |  |  |  |
| --- | --- | --- | --- | --- |
| a. Link function: Logit. |  |  |  |  |
| b. The log-likelihood value cannot be further increased after maximum number of step-halving. |  |  |  |  |
| c. The Chi-Square statistic is computed based on the log-likelihood value of the last iteration of the general model. Validity of the test is uncertain. |  |  |  |  |
