# Supplementary material for: #Yourpalaeolife: Interrogating the Status of Fieldwork Among Early Career Palaeontology Researchers
Source: Ecol Evol. 2026 Jul 29;16(8):e74032. doi: 10.1002/ece3.74032 (PMC13420382; doi:10.1002/ece3.74032)
Supplement: Supplementary file 2 — Data S2: ece374032‐sup‐0002‐Supinfo2.zip. [file ECE3-16-e74032-s002.zip › M77 OLR_RCxGM.docx]

**PLUM - Ordinal Regression**

| **Notes** |  |  |
| --- | --- | --- |
| Output Created |  | 03-FEB-2026 17:03:36 |
| Comments |  |  |
| Input | Active Dataset | DataSet9 |
|  | Filter | <none> |
|  | Weight | <none> |
|  | Split File | <none> |
|  | N of Rows in Working Data File | 157 |
| Missing Value Handling | Definition of Missing | User-defined missing values are treated as missing. |
|  | Cases Used | Statistics are based on all cases with valid data for all variables in the model. |
| Syntax |  | PLUM CGM BY Career_stage Gender_ID Age_category WITH GMNT /CRITERIA=CIN(95) DELTA(0) LCONVERGE(0) MXITER(100) MXSTEP(5) PCONVERGE(1.0E-6) SINGULAR(1.0E-8) /LINK=LOGIT /PRINT=FIT PARAMETER SUMMARY TPARALLEL. |
| Resources | Processor Time | 00:00:00.02 |
|  | Elapsed Time | 00:00:00.02 |

| **Warnings** |
| --- |
| There are 119 (58.0%) cells (i.e., dependent variable levels by observed combinations of predictor variable values) with zero frequencies. |

| **Case Processing Summary** |  |  |  |
| --- | --- | --- | --- |
|  |  | N | Marginal Percentage |
| CGM | 1 | 13 | 9.0% |
|  | 2 | 29 | 20.0% |
|  | 3 | 33 | 22.8% |
|  | 4 | 51 | 35.2% |
|  | 5 | 19 | 13.1% |
| Career_stage | PhD candidate | 79 | 54.5% |
|  | Researcher in palaeontology up to 5 years post-PhD | 66 | 45.5% |
| Gender_ID | F | 61 | 42.1% |
|  | M | 66 | 45.5% |
|  | N | 5 | 3.4% |
|  | U | 13 | 9.0% |
| Age_category | <25 years old | 16 | 11.0% |
|  | 26-30 years old | 56 | 38.6% |
|  | 31-35 years old | 49 | 33.8% |
|  | 36-40 years old | 18 | 12.4% |
|  | 41+ years old | 6 | 4.1% |
| Valid |  | 145 | 100.0% |
| Missing |  | 12 |  |
| Total |  | 157 |  |

| **Model Fitting Information** |  |  |  |  |
| --- | --- | --- | --- | --- |
| Model | -2 Log Likelihood | Chi-Square | df | Sig. |
| Intercept Only | 240.902 |  |  |  |
| Final | 206.714 | 34.188 | 9 | <.001 |

| Link function: Logit. |  |  |  |  |
| --- | --- | --- | --- | --- |

| **Goodness-of-Fit** |  |  |  |
| --- | --- | --- | --- |
|  | Chi-Square | df | Sig. |
| Pearson | 133.088 | 151 | .850 |
| Deviance | 120.687 | 151 | .967 |

| Link function: Logit. |  |  |  |
| --- | --- | --- | --- |

| **Pseudo R-Square** |  |
| --- | --- |
| Cox and Snell | .210 |
| Nagelkerke | .221 |
| McFadden | .078 |

| Link function: Logit. |  |
| --- | --- |

| **Parameter Estimates** |  |  |  |  |  |  |
| --- | --- | --- | --- | --- | --- | --- |
|  |  | Estimate | Std. Error | Wald | df | Sig. |
|  |  |  |  |  |  |  |
| Threshold | [CGM = 1] | -3.210 | .997 | 10.356 | 1 | .001 |
|  | [CGM = 2] | -1.455 | .952 | 2.335 | 1 | .127 |
|  | [CGM = 3] | -.317 | .945 | .113 | 1 | .737 |
|  | [CGM = 4] | 1.647 | .958 | 2.954 | 1 | .086 |
| Location | GMNT | -2.537 | .472 | 28.872 | 1 | <.001 |
|  | [Career_stage=PhD candidate] | .404 | .352 | 1.319 | 1 | .251 |
|  | [Career_stage=Researcher in palaeontology up to 5 years post-PhD] | 0^a^ | . | . | 0 | . |
|  | [Gender_ID=F] | .231 | .561 | .169 | 1 | .681 |
|  | [Gender_ID=M] | .351 | .563 | .388 | 1 | .533 |
|  | [Gender_ID=N] | .181 | .958 | .036 | 1 | .850 |
|  | [Gender_ID=U] | 0^a^ | . | . | 0 | . |
|  | [Age_category=<25 years old] | -1.351 | .912 | 2.196 | 1 | .138 |
|  | [Age_category=26-30 years old] | -.541 | .801 | .456 | 1 | .499 |
|  | [Age_category=31-35 years old] | -.630 | .796 | .627 | 1 | .428 |
|  | [Age_category=36-40 years old] | -.137 | .866 | .025 | 1 | .875 |
|  | [Age_category=41+ years old] | 0^a^ | . | . | 0 | . |

| **Parameter Estimates** |  |  |  |
| --- | --- | --- | --- |
|  |  | 95% Confidence Interval |  |
|  |  | Lower Bound | Upper Bound |
| Threshold | [CGM = 1] | -5.165 | -1.255 |
|  | [CGM = 2] | -3.322 | .411 |
|  | [CGM = 3] | -2.169 | 1.534 |
|  | [CGM = 4] | -.231 | 3.525 |
| Location | GMNT | -3.462 | -1.611 |
|  | [Career_stage=PhD candidate] | -.286 | 1.094 |
|  | [Career_stage=Researcher in palaeontology up to 5 years post-PhD] | . | . |
|  | [Gender_ID=F] | -.870 | 1.331 |
|  | [Gender_ID=M] | -.753 | 1.455 |
|  | [Gender_ID=N] | -1.697 | 2.058 |
|  | [Gender_ID=U] | . | . |
|  | [Age_category=<25 years old] | -3.139 | .436 |
|  | [Age_category=26-30 years old] | -2.112 | 1.030 |
|  | [Age_category=31-35 years old] | -2.190 | .929 |
|  | [Age_category=36-40 years old] | -1.835 | 1.562 |
|  | [Age_category=41+ years old] | . | . |

|  |  |  |  |  |  |  |
| --- | --- | --- | --- | --- | --- | --- |
|  |  |  |  |  |  |  |

| Link function: Logit. |  |  |  |
| --- | --- | --- | --- |
| a. This parameter is set to zero because it is redundant. |  |  |  |

| **Test of Parallel Lines**^a^ |  |  |  |  |
| --- | --- | --- | --- | --- |
| Model | -2 Log Likelihood | Chi-Square | df | Sig. |
| Null Hypothesis | 206.714 |  |  |  |
| General | 187.979^b^ | 18.735^c^ | 27 | .879 |

| The null hypothesis states that the location parameters (slope coefficients) are the same across response categories.^a^ |  |  |  |  |
| --- | --- | --- | --- | --- |
| a. Link function: Logit. |  |  |  |  |
| b. The log-likelihood value cannot be further increased after maximum number of step-halving. |  |  |  |  |
| c. The Chi-Square statistic is computed based on the log-likelihood value of the last iteration of the general model. Validity of the test is uncertain. |  |  |  |  |
