# Supplementary material for: #Yourpalaeolife: Interrogating the Status of Fieldwork Among Early Career Palaeontology Researchers
Source: Ecol Evol. 2026 Jul 29;16(8):e74032. doi: 10.1002/ece3.74032 (PMC13420382; doi:10.1002/ece3.74032)
Supplement: Supplementary file 2 — Data S2: ece374032‐sup‐0002‐Supinfo2.zip. [file ECE3-16-e74032-s002.zip › M79 OLR_RCxGS.docx]

**PLUM - Ordinal Regression**

| **Notes** |  |  |
| --- | --- | --- |
| Output Created |  | 03-FEB-2026 17:02:35 |
| Comments |  |  |
| Input | Active Dataset | DataSet9 |
|  | Filter | <none> |
|  | Weight | <none> |
|  | Split File | <none> |
|  | N of Rows in Working Data File | 157 |
| Missing Value Handling | Definition of Missing | User-defined missing values are treated as missing. |
|  | Cases Used | Statistics are based on all cases with valid data for all variables in the model. |
| Syntax |  | PLUM CGS BY Career_stage Gender_ID Age_category WITH GSNT /CRITERIA=CIN(95) DELTA(0) LCONVERGE(0) MXITER(100) MXSTEP(5) PCONVERGE(1.0E-6) SINGULAR(1.0E-8) /LINK=LOGIT /PRINT=FIT PARAMETER SUMMARY TPARALLEL. |
| Resources | Processor Time | 00:00:00.00 |
|  | Elapsed Time | 00:00:00.01 |

| **Warnings** |
| --- |
| There are 99 (53.5%) cells (i.e., dependent variable levels by observed combinations of predictor variable values) with zero frequencies. |

| **Case Processing Summary** |  |  |  |
| --- | --- | --- | --- |
|  |  | N | Marginal Percentage |
| CGS | 1 | 15 | 10.3% |
|  | 2 | 25 | 17.1% |
|  | 3 | 38 | 26.0% |
|  | 4 | 45 | 30.8% |
|  | 5 | 23 | 15.8% |
| Career_stage | PhD candidate | 79 | 54.1% |
|  | Researcher in palaeontology up to 5 years post-PhD | 67 | 45.9% |
| Gender_ID | F | 63 | 43.2% |
|  | M | 65 | 44.5% |
|  | N | 5 | 3.4% |
|  | U | 13 | 8.9% |
| Age_category | <25 years old | 17 | 11.6% |
|  | 26-30 years old | 57 | 39.0% |
|  | 31-35 years old | 49 | 33.6% |
|  | 36-40 years old | 18 | 12.3% |
|  | 41+ years old | 5 | 3.4% |
| Valid |  | 146 | 100.0% |
| Missing |  | 11 |  |
| Total |  | 157 |  |

| **Model Fitting Information** |  |  |  |  |
| --- | --- | --- | --- | --- |
| Model | -2 Log Likelihood | Chi-Square | df | Sig. |
| Intercept Only | 241.903 |  |  |  |
| Final | 206.358 | 35.546 | 9 | <.001 |

| Link function: Logit. |  |  |  |  |
| --- | --- | --- | --- | --- |

| **Goodness-of-Fit** |  |  |  |
| --- | --- | --- | --- |
|  | Chi-Square | df | Sig. |
| Pearson | 117.331 | 135 | .861 |
| Deviance | 113.594 | 135 | .909 |

| Link function: Logit. |  |  |  |
| --- | --- | --- | --- |

| **Pseudo R-Square** |  |
| --- | --- |
| Cox and Snell | .216 |
| Nagelkerke | .226 |
| McFadden | .079 |

| Link function: Logit. |  |
| --- | --- |

| **Parameter Estimates** |  |  |  |  |  |  |
| --- | --- | --- | --- | --- | --- | --- |
|  |  | Estimate | Std. Error | Wald | df | Sig. |
|  |  |  |  |  |  |  |
| Threshold | [CGS = 1] | -4.338 | 1.062 | 16.687 | 1 | <.001 |
|  | [CGS = 2] | -2.928 | 1.019 | 8.252 | 1 | .004 |
|  | [CGS = 3] | -1.612 | 1.001 | 2.597 | 1 | .107 |
|  | [CGS = 4] | .113 | .997 | .013 | 1 | .910 |
| Location | GSNT | -2.356 | .462 | 25.983 | 1 | <.001 |
|  | [Career_stage=PhD candidate] | -.625 | .358 | 3.049 | 1 | .081 |
|  | [Career_stage=Researcher in palaeontology up to 5 years post-PhD] | 0^a^ | . | . | 0 | . |
|  | [Gender_ID=F] | -.241 | .559 | .186 | 1 | .666 |
|  | [Gender_ID=M] | -.092 | .568 | .026 | 1 | .871 |
|  | [Gender_ID=N] | .267 | .970 | .076 | 1 | .783 |
|  | [Gender_ID=U] | 0^a^ | . | . | 0 | . |
|  | [Age_category=<25 years old] | -1.209 | .980 | 1.521 | 1 | .218 |
|  | [Age_category=26-30 years old] | -.698 | .876 | .635 | 1 | .426 |
|  | [Age_category=31-35 years old] | -1.466 | .869 | 2.845 | 1 | .092 |
|  | [Age_category=36-40 years old] | -.499 | .928 | .289 | 1 | .591 |
|  | [Age_category=41+ years old] | 0^a^ | . | . | 0 | . |

| **Parameter Estimates** |  |  |  |
| --- | --- | --- | --- |
|  |  | 95% Confidence Interval |  |
|  |  | Lower Bound | Upper Bound |
| Threshold | [CGS = 1] | -6.419 | -2.256 |
|  | [CGS = 2] | -4.926 | -.930 |
|  | [CGS = 3] | -3.573 | .349 |
|  | [CGS = 4] | -1.842 | 2.067 |
| Location | GSNT | -3.262 | -1.450 |
|  | [Career_stage=PhD candidate] | -1.326 | .076 |
|  | [Career_stage=Researcher in palaeontology up to 5 years post-PhD] | . | . |
|  | [Gender_ID=F] | -1.336 | .854 |
|  | [Gender_ID=M] | -1.206 | 1.022 |
|  | [Gender_ID=N] | -1.633 | 2.168 |
|  | [Gender_ID=U] | . | . |
|  | [Age_category=<25 years old] | -3.130 | .713 |
|  | [Age_category=26-30 years old] | -2.416 | 1.019 |
|  | [Age_category=31-35 years old] | -3.169 | .238 |
|  | [Age_category=36-40 years old] | -2.318 | 1.320 |
|  | [Age_category=41+ years old] | . | . |

|  |  |  |  |  |  |  |
| --- | --- | --- | --- | --- | --- | --- |
|  |  |  |  |  |  |  |

| Link function: Logit. |  |  |  |
| --- | --- | --- | --- |
| a. This parameter is set to zero because it is redundant. |  |  |  |

| **Test of Parallel Lines**^a^ |  |  |  |  |
| --- | --- | --- | --- | --- |
| Model | -2 Log Likelihood | Chi-Square | df | Sig. |
| Null Hypothesis | 206.358 |  |  |  |
| General | 61.179^b^ | 145.178^c^ | 27 | <.001 |

| The null hypothesis states that the location parameters (slope coefficients) are the same across response categories.^a^ |  |  |  |  |
| --- | --- | --- | --- | --- |
| a. Link function: Logit. |  |  |  |  |
| b. The log-likelihood value cannot be further increased after maximum number of step-halving. |  |  |  |  |
| c. The Chi-Square statistic is computed based on the log-likelihood value of the last iteration of the general model. Validity of the test is uncertain. |  |  |  |  |
