# Supplementary material for: #Yourpalaeolife: Interrogating the Status of Fieldwork Among Early Career Palaeontology Researchers
Source: Ecol Evol. 2026 Jul 29;16(8):e74032. doi: 10.1002/ece3.74032 (PMC13420382; doi:10.1002/ece3.74032)
Supplement: Supplementary file 2 — Data S2: ece374032‐sup‐0002‐Supinfo2.zip. [file ECE3-16-e74032-s002.zip › M81 OLR_RCxNMT.docx]

**PLUM - Ordinal Regression**

| **Notes** |  |  |
| --- | --- | --- |
| Output Created |  | 03-FEB-2026 17:01:15 |
| Comments |  |  |
| Input | Active Dataset | DataSet9 |
|  | Filter | <none> |
|  | Weight | <none> |
|  | Split File | <none> |
|  | N of Rows in Working Data File | 157 |
| Missing Value Handling | Definition of Missing | User-defined missing values are treated as missing. |
|  | Cases Used | Statistics are based on all cases with valid data for all variables in the model. |
| Syntax |  | PLUM CNM BY Career_stage Gender_ID Age_category WITH NMNT /CRITERIA=CIN(95) DELTA(0) LCONVERGE(0) MXITER(100) MXSTEP(5) PCONVERGE(1.0E-6) SINGULAR(1.0E-8) /LINK=LOGIT /PRINT=FIT PARAMETER SUMMARY TPARALLEL. |
| Resources | Processor Time | 00:00:00.02 |
|  | Elapsed Time | 00:00:00.02 |

| **Warnings** |
| --- |
| There are 127 (60.5%) cells (i.e., dependent variable levels by observed combinations of predictor variable values) with zero frequencies. |

| **Case Processing Summary** |  |  |  |
| --- | --- | --- | --- |
|  |  | N | Marginal Percentage |
| CNM | 1 | 27 | 18.6% |
|  | 2 | 22 | 15.2% |
|  | 3 | 35 | 24.1% |
|  | 4 | 34 | 23.4% |
|  | 5 | 27 | 18.6% |
| Career_stage | PhD candidate | 81 | 55.9% |
|  | Researcher in palaeontology up to 5 years post-PhD | 64 | 44.1% |
| Gender_ID | F | 63 | 43.4% |
|  | M | 64 | 44.1% |
|  | N | 5 | 3.4% |
|  | U | 13 | 9.0% |
| Age_category | <25 years old | 17 | 11.7% |
|  | 26-30 years old | 57 | 39.3% |
|  | 31-35 years old | 48 | 33.1% |
|  | 36-40 years old | 17 | 11.7% |
|  | 41+ years old | 6 | 4.1% |
| Valid |  | 145 | 100.0% |
| Missing |  | 12 |  |
| Total |  | 157 |  |

| **Model Fitting Information** |  |  |  |  |
| --- | --- | --- | --- | --- |
| Model | -2 Log Likelihood | Chi-Square | df | Sig. |
| Intercept Only | 306.506 |  |  |  |
| Final | 213.250 | 93.257 | 9 | <.001 |

| Link function: Logit. |  |  |  |  |
| --- | --- | --- | --- | --- |

| **Goodness-of-Fit** |  |  |  |
| --- | --- | --- | --- |
|  | Chi-Square | df | Sig. |
| Pearson | 147.212 | 155 | .660 |
| Deviance | 136.782 | 155 | .851 |

| Link function: Logit. |  |  |  |
| --- | --- | --- | --- |

| **Pseudo R-Square** |  |
| --- | --- |
| Cox and Snell | .474 |
| Nagelkerke | .495 |
| McFadden | .202 |

| Link function: Logit. |  |
| --- | --- |

| **Parameter Estimates** |  |  |  |  |  |  |
| --- | --- | --- | --- | --- | --- | --- |
|  |  | Estimate | Std. Error | Wald | df | Sig. |
|  |  |  |  |  |  |  |
| Threshold | [CNM = 1] | -4.545 | 1.073 | 17.955 | 1 | <.001 |
|  | [CNM = 2] | -3.133 | 1.028 | 9.284 | 1 | .002 |
|  | [CNM = 3] | -1.449 | 1.000 | 2.099 | 1 | .147 |
|  | [CNM = 4] | .058 | .999 | .003 | 1 | .953 |
| Location | NMNT | -3.540 | .444 | 63.480 | 1 | <.001 |
|  | [Career_stage=PhD candidate] | -.258 | .359 | .516 | 1 | .473 |
|  | [Career_stage=Researcher in palaeontology up to 5 years post-PhD] | 0^a^ | . | . | 0 | . |
|  | [Gender_ID=F] | .719 | .581 | 1.531 | 1 | .216 |
|  | [Gender_ID=M] | 1.066 | .597 | 3.184 | 1 | .074 |
|  | [Gender_ID=N] | .655 | .974 | .452 | 1 | .502 |
|  | [Gender_ID=U] | 0^a^ | . | . | 0 | . |
|  | [Age_category=<25 years old] | -1.760 | .956 | 3.385 | 1 | .066 |
|  | [Age_category=26-30 years old] | -1.609 | .857 | 3.523 | 1 | .061 |
|  | [Age_category=31-35 years old] | -1.862 | .854 | 4.758 | 1 | .029 |
|  | [Age_category=36-40 years old] | -.825 | .922 | .800 | 1 | .371 |
|  | [Age_category=41+ years old] | 0^a^ | . | . | 0 | . |

| **Parameter Estimates** |  |  |  |
| --- | --- | --- | --- |
|  |  | 95% Confidence Interval |  |
|  |  | Lower Bound | Upper Bound |
| Threshold | [CNM = 1] | -6.647 | -2.443 |
|  | [CNM = 2] | -5.148 | -1.118 |
|  | [CNM = 3] | -3.408 | .511 |
|  | [CNM = 4] | -1.899 | 2.016 |
| Location | NMNT | -4.411 | -2.669 |
|  | [Career_stage=PhD candidate] | -.962 | .446 |
|  | [Career_stage=Researcher in palaeontology up to 5 years post-PhD] | . | . |
|  | [Gender_ID=F] | -.420 | 1.859 |
|  | [Gender_ID=M] | -.105 | 2.236 |
|  | [Gender_ID=N] | -1.255 | 2.564 |
|  | [Gender_ID=U] | . | . |
|  | [Age_category=<25 years old] | -3.634 | .115 |
|  | [Age_category=26-30 years old] | -3.290 | .071 |
|  | [Age_category=31-35 years old] | -3.535 | -.189 |
|  | [Age_category=36-40 years old] | -2.631 | .982 |
|  | [Age_category=41+ years old] | . | . |

|  |  |  |  |  |  |  |
| --- | --- | --- | --- | --- | --- | --- |
|  |  |  |  |  |  |  |

| Link function: Logit. |  |  |  |
| --- | --- | --- | --- |
| a. This parameter is set to zero because it is redundant. |  |  |  |

| **Test of Parallel Lines**^a^ |  |  |  |  |
| --- | --- | --- | --- | --- |
| Model | -2 Log Likelihood | Chi-Square | df | Sig. |
| Null Hypothesis | 213.250 |  |  |  |
| General | 192.527^b^ | 20.722^c^ | 27 | .799 |

| The null hypothesis states that the location parameters (slope coefficients) are the same across response categories.^a^ |  |  |  |  |
| --- | --- | --- | --- | --- |
| a. Link function: Logit. |  |  |  |  |
| b. The log-likelihood value cannot be further increased after maximum number of step-halving. |  |  |  |  |
| c. The Chi-Square statistic is computed based on the log-likelihood value of the last iteration of the general model. Validity of the test is uncertain. |  |  |  |  |
