# Supplementary material for: #Yourpalaeolife: Interrogating the Status of Fieldwork Among Early Career Palaeontology Researchers
Source: Ecol Evol. 2026 Jul 29;16(8):e74032. doi: 10.1002/ece3.74032 (PMC13420382; doi:10.1002/ece3.74032)
Supplement: Supplementary file 2 — Data S2: ece374032‐sup‐0002‐Supinfo2.zip. [file ECE3-16-e74032-s002.zip › M83 OLR_RCxSS.docx]

**PLUM - Ordinal Regression**

| **Notes** |  |  |
| --- | --- | --- |
| Output Created |  | 03-FEB-2026 17:05:37 |
| Comments |  |  |
| Input | Active Dataset | DataSet9 |
|  | Filter | <none> |
|  | Weight | <none> |
|  | Split File | <none> |
|  | N of Rows in Working Data File | 157 |
| Missing Value Handling | Definition of Missing | User-defined missing values are treated as missing. |
|  | Cases Used | Statistics are based on all cases with valid data for all variables in the model. |
| Syntax |  | PLUM CSS BY Career_stage Gender_ID Age_category WITH SSNT /CRITERIA=CIN(95) DELTA(0) LCONVERGE(0) MXITER(100) MXSTEP(5) PCONVERGE(1.0E-6) SINGULAR(1.0E-8) /LINK=LOGIT /PRINT=FIT PARAMETER SUMMARY TPARALLEL. |
| Resources | Processor Time | 00:00:00.00 |
|  | Elapsed Time | 00:00:00.01 |

| **Warnings** |
| --- |
| There are 134 (60.9%) cells (i.e., dependent variable levels by observed combinations of predictor variable values) with zero frequencies. |

| **Case Processing Summary** |  |  |  |
| --- | --- | --- | --- |
|  |  | N | Marginal Percentage |
| CSS | 1 | 7 | 4.8% |
|  | 2 | 23 | 15.9% |
|  | 3 | 30 | 20.7% |
|  | 4 | 60 | 41.4% |
|  | 5 | 25 | 17.2% |
| Career_stage | PhD candidate | 80 | 55.2% |
|  | Researcher in palaeontology up to 5 years post-PhD | 65 | 44.8% |
| Gender_ID | F | 60 | 41.4% |
|  | M | 66 | 45.5% |
|  | N | 6 | 4.1% |
|  | U | 13 | 9.0% |
| Age_category | <25 years old | 16 | 11.0% |
|  | 26-30 years old | 55 | 37.9% |
|  | 31-35 years old | 50 | 34.5% |
|  | 36-40 years old | 17 | 11.7% |
|  | 41+ years old | 7 | 4.8% |
| Valid |  | 145 | 100.0% |
| Missing |  | 12 |  |
| Total |  | 157 |  |

| **Model Fitting Information** |  |  |  |  |
| --- | --- | --- | --- | --- |
| Model | -2 Log Likelihood | Chi-Square | df | Sig. |
| Intercept Only | 268.378 |  |  |  |
| Final | 203.991 | 64.387 | 9 | <.001 |

| Link function: Logit. |  |  |  |  |
| --- | --- | --- | --- | --- |

| **Goodness-of-Fit** |  |  |  |
| --- | --- | --- | --- |
|  | Chi-Square | df | Sig. |
| Pearson | 172.056 | 163 | .298 |
| Deviance | 128.492 | 163 | .979 |

| Link function: Logit. |  |  |  |
| --- | --- | --- | --- |

| **Pseudo R-Square** |  |
| --- | --- |
| Cox and Snell | .359 |
| Nagelkerke | .380 |
| McFadden | .155 |

| Link function: Logit. |  |
| --- | --- |

| **Parameter Estimates** |  |  |  |  |  |  |
| --- | --- | --- | --- | --- | --- | --- |
|  |  | Estimate | Std. Error | Wald | df | Sig. |
|  |  |  |  |  |  |  |
| Threshold | [CSS = 1] | -5.383 | 1.066 | 25.497 | 1 | <.001 |
|  | [CSS = 2] | -3.384 | .992 | 11.633 | 1 | <.001 |
|  | [CSS = 3] | -2.020 | .963 | 4.405 | 1 | .036 |
|  | [CSS = 4] | .553 | .946 | .342 | 1 | .559 |
| Location | SSNT | -2.575 | .386 | 44.425 | 1 | <.001 |
|  | [Career_stage=PhD candidate] | .185 | .360 | .265 | 1 | .607 |
|  | [Career_stage=Researcher in palaeontology up to 5 years post-PhD] | 0^a^ | . | . | 0 | . |
|  | [Gender_ID=F] | -.672 | .592 | 1.292 | 1 | .256 |
|  | [Gender_ID=M] | .397 | .593 | .450 | 1 | .503 |
|  | [Gender_ID=N] | .525 | .935 | .315 | 1 | .575 |
|  | [Gender_ID=U] | 0^a^ | . | . | 0 | . |
|  | [Age_category=<25 years old] | -1.300 | .891 | 2.130 | 1 | .144 |
|  | [Age_category=26-30 years old] | -.931 | .777 | 1.438 | 1 | .231 |
|  | [Age_category=31-35 years old] | -.488 | .771 | .401 | 1 | .527 |
|  | [Age_category=36-40 years old] | .322 | .859 | .141 | 1 | .708 |
|  | [Age_category=41+ years old] | 0^a^ | . | . | 0 | . |

| **Parameter Estimates** |  |  |  |
| --- | --- | --- | --- |
|  |  | 95% Confidence Interval |  |
|  |  | Lower Bound | Upper Bound |
| Threshold | [CSS = 1] | -7.472 | -3.293 |
|  | [CSS = 2] | -5.329 | -1.439 |
|  | [CSS = 3] | -3.907 | -.134 |
|  | [CSS = 4] | -1.302 | 2.408 |
| Location | SSNT | -3.332 | -1.818 |
|  | [Career_stage=PhD candidate] | -.521 | .892 |
|  | [Career_stage=Researcher in palaeontology up to 5 years post-PhD] | . | . |
|  | [Gender_ID=F] | -1.832 | .487 |
|  | [Gender_ID=M] | -.764 | 1.559 |
|  | [Gender_ID=N] | -1.308 | 2.359 |
|  | [Gender_ID=U] | . | . |
|  | [Age_category=<25 years old] | -3.045 | .446 |
|  | [Age_category=26-30 years old] | -2.453 | .591 |
|  | [Age_category=31-35 years old] | -2.001 | 1.024 |
|  | [Age_category=36-40 years old] | -1.362 | 2.006 |
|  | [Age_category=41+ years old] | . | . |

|  |  |  |  |  |  |  |
| --- | --- | --- | --- | --- | --- | --- |
|  |  |  |  |  |  |  |

| Link function: Logit. |  |  |  |
| --- | --- | --- | --- |
| a. This parameter is set to zero because it is redundant. |  |  |  |

| **Test of Parallel Lines**^a^ |  |  |  |  |
| --- | --- | --- | --- | --- |
| Model | -2 Log Likelihood | Chi-Square | df | Sig. |
| Null Hypothesis | 203.991 |  |  |  |
| General | 159.886^b^ | 44.105^c^ | 27 | .020 |

| The null hypothesis states that the location parameters (slope coefficients) are the same across response categories.^a^ |  |  |  |  |
| --- | --- | --- | --- | --- |
| a. Link function: Logit. |  |  |  |  |
| b. The log-likelihood value cannot be further increased after maximum number of step-halving. |  |  |  |  |
| c. The Chi-Square statistic is computed based on the log-likelihood value of the last iteration of the general model. Validity of the test is uncertain. |  |  |  |  |
