# Supplementary material for: #Yourpalaeolife: Interrogating the Status of Fieldwork Among Early Career Palaeontology Researchers
Source: Ecol Evol. 2026 Jul 29;16(8):e74032. doi: 10.1002/ece3.74032 (PMC13420382; doi:10.1002/ece3.74032)
Supplement: Supplementary file 2 — Data S2: ece374032‐sup‐0002‐Supinfo2.zip. [file ECE3-16-e74032-s002.zip › M85 OLR_RCxTP.docx]

**PLUM - Ordinal Regression**

| **Notes** |  |  |
| --- | --- | --- |
| Output Created |  | 03-FEB-2026 17:04:34 |
| Comments |  |  |
| Input | Active Dataset | DataSet9 |
|  | Filter | <none> |
|  | Weight | <none> |
|  | Split File | <none> |
|  | N of Rows in Working Data File | 157 |
| Missing Value Handling | Definition of Missing | User-defined missing values are treated as missing. |
|  | Cases Used | Statistics are based on all cases with valid data for all variables in the model. |
| Syntax |  | PLUM CTP BY Career_stage Gender_ID Age_category WITH TPNT /CRITERIA=CIN(95) DELTA(0) LCONVERGE(0) MXITER(100) MXSTEP(5) PCONVERGE(1.0E-6) SINGULAR(1.0E-8) /LINK=LOGIT /PRINT=FIT PARAMETER SUMMARY TPARALLEL. |
| Resources | Processor Time | 00:00:00.02 |
|  | Elapsed Time | 00:00:00.03 |

| **Warnings** |
| --- |
| There are 121 (57.6%) cells (i.e., dependent variable levels by observed combinations of predictor variable values) with zero frequencies. |

| **Case Processing Summary** |  |  |  |
| --- | --- | --- | --- |
|  |  | N | Marginal Percentage |
| CTP | 1 | 16 | 10.7% |
|  | 2 | 27 | 18.1% |
|  | 3 | 33 | 22.1% |
|  | 4 | 54 | 36.2% |
|  | 5 | 19 | 12.8% |
| Career_stage | PhD candidate | 82 | 55.0% |
|  | Researcher in palaeontology up to 5 years post-PhD | 67 | 45.0% |
| Gender_ID | F | 64 | 43.0% |
|  | M | 67 | 45.0% |
|  | N | 5 | 3.4% |
|  | U | 13 | 8.7% |
| Age_category | <25 years old | 18 | 12.1% |
|  | 26-30 years old | 56 | 37.6% |
|  | 31-35 years old | 50 | 33.6% |
|  | 36-40 years old | 18 | 12.1% |
|  | 41+ years old | 7 | 4.7% |
| Valid |  | 149 | 100.0% |
| Missing |  | 8 |  |
| Total |  | 157 |  |

| **Model Fitting Information** |  |  |  |  |
| --- | --- | --- | --- | --- |
| Model | -2 Log Likelihood | Chi-Square | df | Sig. |
| Intercept Only | 265.548 |  |  |  |
| Final | 211.200 | 54.348 | 9 | <.001 |

| Link function: Logit. |  |  |  |  |
| --- | --- | --- | --- | --- |

| **Goodness-of-Fit** |  |  |  |
| --- | --- | --- | --- |
|  | Chi-Square | df | Sig. |
| Pearson | 124.427 | 155 | .966 |
| Deviance | 124.053 | 155 | .968 |

| Link function: Logit. |  |  |  |
| --- | --- | --- | --- |

| **Pseudo R-Square** |  |
| --- | --- |
| Cox and Snell | .306 |
| Nagelkerke | .321 |
| McFadden | .121 |

| Link function: Logit. |  |
| --- | --- |

| **Parameter Estimates** |  |  |  |  |  |  |
| --- | --- | --- | --- | --- | --- | --- |
|  |  | Estimate | Std. Error | Wald | df | Sig. |
|  |  |  |  |  |  |  |
| Threshold | [CTP = 1] | -4.317 | .993 | 18.898 | 1 | <.001 |
|  | [CTP = 2] | -2.701 | .953 | 8.034 | 1 | .005 |
|  | [CTP = 3] | -1.438 | .934 | 2.370 | 1 | .124 |
|  | [CTP = 4] | .754 | .927 | .663 | 1 | .416 |
| Location | TPNT | -2.493 | .410 | 36.972 | 1 | <.001 |
|  | [Career_stage=PhD candidate] | -.121 | .352 | .118 | 1 | .731 |
|  | [Career_stage=Researcher in palaeontology up to 5 years post-PhD] | 0^a^ | . | . | 0 | . |
|  | [Gender_ID=F] | -.582 | .570 | 1.042 | 1 | .307 |
|  | [Gender_ID=M] | .065 | .572 | .013 | 1 | .909 |
|  | [Gender_ID=N] | -.046 | .970 | .002 | 1 | .962 |
|  | [Gender_ID=U] | 0^a^ | . | . | 0 | . |
|  | [Age_category=<25 years old] | -1.244 | .861 | 2.091 | 1 | .148 |
|  | [Age_category=26-30 years old] | -.733 | .766 | .916 | 1 | .339 |
|  | [Age_category=31-35 years old] | -.921 | .768 | 1.440 | 1 | .230 |
|  | [Age_category=36-40 years old] | .066 | .840 | .006 | 1 | .937 |
|  | [Age_category=41+ years old] | 0^a^ | . | . | 0 | . |

| **Parameter Estimates** |  |  |  |
| --- | --- | --- | --- |
|  |  | 95% Confidence Interval |  |
|  |  | Lower Bound | Upper Bound |
| Threshold | [CTP = 1] | -6.264 | -2.371 |
|  | [CTP = 2] | -4.570 | -.833 |
|  | [CTP = 3] | -3.268 | .393 |
|  | [CTP = 4] | -1.062 | 2.571 |
| Location | TPNT | -3.296 | -1.689 |
|  | [Career_stage=PhD candidate] | -.811 | .568 |
|  | [Career_stage=Researcher in palaeontology up to 5 years post-PhD] | . | . |
|  | [Gender_ID=F] | -1.700 | .536 |
|  | [Gender_ID=M] | -1.055 | 1.186 |
|  | [Gender_ID=N] | -1.947 | 1.855 |
|  | [Gender_ID=U] | . | . |
|  | [Age_category=<25 years old] | -2.931 | .442 |
|  | [Age_category=26-30 years old] | -2.235 | .768 |
|  | [Age_category=31-35 years old] | -2.426 | .584 |
|  | [Age_category=36-40 years old] | -1.580 | 1.712 |
|  | [Age_category=41+ years old] | . | . |

|  |  |  |  |  |  |  |
| --- | --- | --- | --- | --- | --- | --- |
|  |  |  |  |  |  |  |

| Link function: Logit. |  |  |  |
| --- | --- | --- | --- |
| a. This parameter is set to zero because it is redundant. |  |  |  |

| **Test of Parallel Lines**^a^ |  |  |  |  |
| --- | --- | --- | --- | --- |
| Model | -2 Log Likelihood | Chi-Square | df | Sig. |
| Null Hypothesis | 211.200 |  |  |  |
| General | 194.146^b^ | 17.055^c^ | 27 | .930 |

| The null hypothesis states that the location parameters (slope coefficients) are the same across response categories.^a^ |  |  |  |  |
| --- | --- | --- | --- | --- |
| a. Link function: Logit. |  |  |  |  |
| b. The log-likelihood value cannot be further increased after maximum number of step-halving. |  |  |  |  |
| c. The Chi-Square statistic is computed based on the log-likelihood value of the last iteration of the general model. Validity of the test is uncertain. |  |  |  |  |
