# Supplementary material for: #Yourpalaeolife: Interrogating the Status of Fieldwork Among Early Career Palaeontology Researchers
Source: Ecol Evol. 2026 Jul 29;16(8):e74032. doi: 10.1002/ece3.74032 (PMC13420382; doi:10.1002/ece3.74032)
Supplement: Supplementary file 2 — Data S2: ece374032‐sup‐0002‐Supinfo2.zip. [file ECE3-16-e74032-s002.zip › M88 ConfidencexCareer Stage SPSS.docx]

**NPar Tests**

| **Notes** |  |  |
| --- | --- | --- |
| Output Created |  | 26-JUN-2026 14:46:41 |
| Comments |  |  |
| Input | Active Dataset | DataSet0 |
|  | Filter | <none> |
|  | Weight | <none> |
|  | Split File | <none> |
|  | N of Rows in Working Data File | 157 |
| Missing Value Handling | Definition of Missing | User-defined missing values are treated as missing. |
|  | Cases Used | Statistics for each test are based on all cases with valid data for the variable(s) used in that test. |
| Syntax |  | NPAR TESTS /M-W= CFID CFEX CFJ CNM CGS CGM CTP CSS CFTM CEL BY Group(1 2) /MISSING ANALYSIS. |
| Resources | Processor Time | 00:00:00.02 |
|  | Elapsed Time | 00:00:00.01 |
|  | Number of Cases Allowed^a^ | 196608 |

| a. Based on availability of workspace memory. |  |  |
| --- | --- | --- |

[DataSet0]

**Mann-Whitney Test**

| **Ranks** |  |  |  |  |
| --- | --- | --- | --- | --- |
|  | Career_stage | N | Mean Rank | Sum of Ranks |
| CFID | 1.00 | 88 | 74.82 | 6584.00 |
|  | 2.00 | 68 | 83.26 | 5662.00 |
|  | Total | 156 |  |  |
| CFEX | 1.00 | 88 | 75.06 | 6605.50 |
|  | 2.00 | 68 | 82.95 | 5640.50 |
|  | Total | 156 |  |  |
| CFJ | 1.00 | 88 | 74.63 | 6567.00 |
|  | 2.00 | 67 | 82.43 | 5523.00 |
|  | Total | 155 |  |  |
| CNM | 1.00 | 89 | 74.20 | 6603.50 |
|  | 2.00 | 67 | 84.22 | 5642.50 |
|  | Total | 156 |  |  |
| CGS | 1.00 | 88 | 73.23 | 6444.50 |
|  | 2.00 | 67 | 84.26 | 5645.50 |
|  | Total | 155 |  |  |
| CGM | 1.00 | 89 | 77.38 | 6887.00 |
|  | 2.00 | 67 | 79.99 | 5359.00 |
|  | Total | 156 |  |  |
| CTP | 1.00 | 88 | 72.26 | 6359.00 |
|  | 2.00 | 67 | 85.54 | 5731.00 |
|  | Total | 155 |  |  |
| CSS | 1.00 | 88 | 77.20 | 6793.50 |
|  | 2.00 | 67 | 79.05 | 5296.50 |
|  | Total | 155 |  |  |
| CFTM | 1.00 | 89 | 72.48 | 6450.50 |
|  | 2.00 | 66 | 85.45 | 5639.50 |
|  | Total | 155 |  |  |
| CEL | 1.00 | 89 | 71.31 | 6346.50 |
|  | 2.00 | 68 | 89.07 | 6056.50 |
|  | Total | 157 |  |  |

| **Test Statistics**^a^ |  |  |  |  |  |  |
| --- | --- | --- | --- | --- | --- | --- |
|  | CFID | CFEX | CFJ | CNM | CGS | CGM |
| Mann-Whitney U | 2668.000 | 2689.500 | 2651.000 | 2598.500 | 2528.500 | 2882.000 |
| Wilcoxon W | 6584.000 | 6605.500 | 6567.000 | 6603.500 | 6444.500 | 6887.000 |
| Z | -1.208 | -1.146 | -1.100 | -1.402 | -1.557 | -.367 |
| Asymp. Sig. (2-tailed) | .227 | .252 | .271 | .161 | .119 | .713 |

| **Test Statistics**^a^ |  |  |  |  |
| --- | --- | --- | --- | --- |
|  | CTP | CSS | CFTM | CEL |
| Mann-Whitney U | 2443.000 | 2877.500 | 2445.500 | 2341.500 |
| Wilcoxon W | 6359.000 | 6793.500 | 6450.500 | 6346.500 |
| Z | -1.887 | -.266 | -1.822 | -2.491 |
| Asymp. Sig. (2-tailed) | .059 | .790 | .068 | .013 |

|  |  |  |  |  |  |  |
| --- | --- | --- | --- | --- | --- | --- |

| a. Grouping Variable: Career_stage |  |  |  |  |
| --- | --- | --- | --- | --- |
