# Supplementary material for: #Yourpalaeolife: Interrogating the Status of Fieldwork Among Early Career Palaeontology Researchers
Source: Ecol Evol. 2026 Jul 29;16(8):e74032. doi: 10.1002/ece3.74032 (PMC13420382; doi:10.1002/ece3.74032)
Supplement: Supplementary file 2 — Data S2: ece374032‐sup‐0002‐Supinfo2.zip. [file ECE3-16-e74032-s002.zip › M89 ConfidencexGenderID SPSS.docx]

**Mann-Whitney Test**

| **Ranks** | | | | |
| --- | --- | --- | --- | --- |
|  | Gender_ID | N | Mean Rank | Sum of Ranks |
| CFID | 1 | 68 | 60.16 | 4091.00 |
|  | 2 | 69 | 77.71 | 5362.00 |
|  | Total | 137 |  |  |
| CFEX | 1 | 68 | 56.38 | 3833.50 |
|  | 2 | 69 | 81.44 | 5619.50 |
|  | Total | 137 |  |  |
| CFJ | 1 | 68 | 57.84 | 3933.00 |
|  | 2 | 68 | 79.16 | 5383.00 |
|  | Total | 136 |  |  |
| CNM | 1 | 69 | 70.05 | 4833.50 |
|  | 2 | 68 | 67.93 | 4619.50 |
|  | Total | 137 |  |  |
| CGS | 1 | 68 | 66.88 | 4548.00 |
|  | 2 | 68 | 70.12 | 4768.00 |
|  | Total | 136 |  |  |
| CGM | 1 | 69 | 66.37 | 4579.50 |
|  | 2 | 68 | 71.67 | 4873.50 |
|  | Total | 137 |  |  |
| CTP | 1 | 68 | 62.34 | 4239.00 |
|  | 2 | 68 | 74.66 | 5077.00 |
|  | Total | 136 |  |  |
| CSS | 1 | 68 | 61.97 | 4214.00 |
|  | 2 | 68 | 75.03 | 5102.00 |
|  | Total | 136 |  |  |
| CFTM | 1 | 69 | 60.55 | 4178.00 |
|  | 2 | 67 | 76.69 | 5138.00 |
|  | Total | 136 |  |  |
| CEL | 1 | 69 | 64.98 | 4483.50 |
|  | 2 | 69 | 74.02 | 5107.50 |
|  | Total | 138 |  |  |

| **Test Statistics**^a^ | | | | | | |
| --- | --- | --- | --- | --- | --- | --- |
|  | CFID | CFEX | CFJ | CNM | CGS | CGM |
| Mann-Whitney U | 1745.000 | 1487.500 | 1587.000 | 2273.500 | 2202.000 | 2164.500 |
| Wilcoxon W | 4091.000 | 3833.500 | 3933.000 | 4619.500 | 4548.000 | 4579.500 |
| Z | -2.706 | -3.930 | -3.236 | -.319 | -.493 | -.806 |
| Asymp. Sig. (2-tailed) | .007 | <.001 | .001 | .749 | .622 | .420 |

| **Test Statistics**^a^ | | | | |  |  |
| --- | --- | --- | --- | --- | --- | --- |
|  | CTP | CSS | CFTM | CEL |  |  |
| Mann-Whitney U | 1893.000 | 1868.000 | 1763.000 | 2068.500 |  |  |
| Wilcoxon W | 4239.000 | 4214.000 | 4178.000 | 4483.500 |  |  |
| Z | -1.895 | -2.023 | -2.444 | -1.367 |  |  |
| Asymp. Sig. (2-tailed) | .058 | .043 | .015 | .172 |  |  |
|  |  |  |  |  |  |  |

| a. Grouping Variable: Gender_ID |
| --- |
