# Supplementary material for: #Yourpalaeolife: Interrogating the Status of Fieldwork Among Early Career Palaeontology Researchers
Source: Ecol Evol. 2026 Jul 29;16(8):e74032. doi: 10.1002/ece3.74032 (PMC13420382; doi:10.1002/ece3.74032)
Supplement: Supplementary file 3 — Data S3: ece374032‐sup‐0003‐Supinfo3.zip. [file ECE3-16-e74032-s001.zip › D1 #Yourpalaeolife_ the experience of early career palaeontologists and fieldwork PDF version.pdf]

#Yourpalaeolife: the experience of early career palaeontologists and fieldwork

Hello everybody!

Welcome to my anonymous survey about fieldwork in palaeontology. I want to know about your experiences: what is the place of fieldwork in our research lives, how to we view it, and how do we participate if we want it? The survey is completely anonymous and I want to hear the unfiltered truth about your opinions of fieldwork, its ethical quandaries, the barriers you and your peers have or have not faced in finding those early career fieldwork trips and skills training opportunities. Even if you have no interest in fieldwork at all and have never sought it out – that's important too. Eligible candidates are **current PhD students and post-docs (less than 5 years post-PhD) whose field of work is Palaeontology**. Responses from anywhere in the world are invited - this is a global research community. We will start with four quick consent questions, and end with some optional demographic questions. No direct feedback will be provided to candidates after the survey, so do follow @whosyourmammal.bsky.social or #yourpalaeolife on Bluesky for further updates regarding results and publications.

This survey will close at 12pm GMT on 16th November 2025. Participants are reminded that if they require support regarding sensitive issues mentioned in this survey, they can contact their local services.

* Indicates required question

1. I understand that the survey is anonymous and that no attempt to contact or identify me will be made by the research team as a result of my responses. I also understand that the anonymous nature of the survey will mean I cannot withdraw my response once the survey form is submitted, but that I can stop filling in the survey form at any time before submission and my data will not be collected. *

Mark only one oval.

- I understand and consent
- I do not understand/consent and do not wish to participate in the survey

2. I understand and consent that the data I provide will be used to produce a conference poster and/or journal article for open access publication, and that the raw data of the answers to multiple choice and 'select all that apply' questions will be published as Supplementary Material. *

Mark only one oval.

- I understand and consent, and wish to participate in the survey
- I do not consent and do not wish to participate in the survey

3. I consent to the text of my free-response answers (open text box answers) being published, whole or in part, as part of the conference poster and/or journal article and/or Supplementary Material. *

Mark only one oval.

- I consent to my answers being used in this way
- I do not consent to my answers being used in this way but I DO wish to participate in the survey
- I do not consent to my answers being used in this way and I do NOT wish to participate further

4. I understand that the response data will be stored in accordance with University of Birmingham (UK) standard practice for post-graduate research projects. *

Mark only one oval.

- I understand and consent
- I do not understand/consent and do not wish to participate in the survey

5. Question 1: What stage are you at in your palaeontology career? *

Mark only one oval.

- PhD candidate
- Researcher in palaeontology up to 5 years post-PhD

6. Question 2: Do you have, or have you ever had, a personal interest in pursuing fieldwork as part of your research? *

Mark only one oval.

- Yes, I have an active interest in pursuing fieldwork
- Yes, I have previously had an interest in fieldwork, but now I do not
- No, I have never had an interest in pursuing fieldwork

7. Question 3: Did you have any fieldwork experience or field skills training (e.g. site identification and mapping, fossil excavation, geological sequencing etc.) prior to the start of your PhD? Check all that apply. *

Tick all that apply.

- [ ] Yes, I had fieldwork experience from my undergraduate programme
- [ ] Yes, I had fieldwork experience from my Masters degree programme
- [ ] I had field skills training from my undergraduate and/or Masters degree programmes
- [ ] I had no fieldwork experience or field skills training prior to my PhD studies because I chose not to participate in any
- [ ] I had no fieldwork experience or field skills training prior to my PhD studies because I had no viable opportunities to undertake any
- [ ] I did not study an undergraduate or Masters programme in Palaeontology or a closely related discipline
- [ ] Other: ______

8. Question 4: With which field skills have you had formal training so far? Select multiple answers if applicable.

Tick all that apply.

	Yes, training prior to my PhD	Yes, training during my PhD	Yes, training after completion of my PhD	No formal training
**Fossil identification and dating**	☐	☐	☐	☐
**Fossil excavation**	☐	☐	☐	☐
**Fossil jacketing**	☐	☐	☐	☐
**Non-macrofossil data collection (e.g. geological sampling, palynomorph sample collection, microfossil collection)**	☐	☐	☐	☐
**Geological sequence interpretation**	☐	☐	☐	☐
**Geological mapping and/or other field site mapping**	☐	☐	☐	☐
**Identification of taphonomic processes in the field**	☐	☐	☐	☐
**Safety and site management**	☐	☐	☐	☐
**Field team management**	☐	☐	☐	☐
**Practical application of fieldwork ethics and law**	☐	☐	☐	☐

9. Question 5: Please indicate your current level of confidence using the following field skills, regardless of formal training.

Mark only one oval per row.

	Not at all confident	Not very confident	Neutral	Quite confident	Very confident
**Fossil identification and dating**	⬭	⬭	⬭	⬭	⬭
**Fossil excavation**	⬭	⬭	⬭	⬭	⬭
**Fossil jacketing**	⬭	⬭	⬭	⬭	⬭
**Non-macrofossil data collection (e.g. geological sampling, palynomorph sample collection, microfossil collection)**	⬭	⬭	⬭	⬭	⬭
**Geological sequence interpretation**	⬭	⬭	⬭	⬭	⬭
**Geological mapping and/or other field site mapping**	⬭	⬭	⬭	⬭	⬭
**Identification of taphonomic processes in the field**	⬭	⬭	⬭	⬭	⬭
**Safety and site management**	⬭	⬭	⬭	⬭	⬭
**Field team management**	⬭	⬭	⬭	⬭	⬭
**Practical application of fieldwork ethics and law**	⬭	⬭	⬭	⬭	⬭

10. Question 6: Are there any other field skills you feel are particularly important? Have you been able to find/participate in any formal training for them?

____
____
____
____
____

11. Question 7: Have you faced any of the following barriers to obtaining field skills training? Select all that apply. If you have encountered no barriers to obtaining training, select 'No'.

Tick all that apply.

- ☐ Lack of professional funding
- ☐ Lack of training in my geographical region
- ☐ Lack of training provided by my institution
- ☐ Lack of logistical support from my institution or managerial team (e.g. time away from studies/work to participate in training)
- ☐ Lack of other personal development support from my institution or managerial team (e.g. encouragement)
- ☐ Poor advertising of training opportunities
- ☐ High cost of training courses (regardless of professional funding availability)
- ☐ Personal circumstances (e.g. ill health, family responsibilities)
- ☐ Requirement for accessibility adjustments
- ☐ Lack of personal or professional confidence
- ☐ Concerns about travel safety
- ☐ Uncertainty about which field skills I might need or find useful
- ☐ No
- ☐ Don't know where to start looking
- ☐ Discrimination (this will be addressed in more detail in question 8)
- ☐ Other: _______________________________

12. Question 8: Have you experienced or perceived any form of discrimination against yourself or others during your search for or experiences of palaeontology field skills training, on the basis of the following characteristics? Select all that apply.

Tick all that apply.

- [ ] Ethnicity
- [ ] Gender
- [ ] Sexual orientation
- [ ] Professional mode of work (e.g. part time work, remote work etc.)
- [ ] Professional colleague relationships within your institution (i.e. who you have or have not worked with previously, intradepartmental conflicts)
- [ ] Professional colleague relationships outside your institution (i.e. who you have or have not worked with previously, inter-institutional conflicts)
- [ ] Religion
- [ ] Pregnancy or parental leave
- [ ] Age
- [ ] Disability or requirement for accessibility accommodations
- [ ] No discrimination encountered
- [ ] Prefer not to say
- [ ] Other: ______________________

13. Question 9: Please use this question to tell us anything else about your experiences surrounding field skills training specifically, and any suggestions you may have for improving access to such training for a person in your position.

____
____
____
____
____

14. Question 10: In the last 3 years, have you sought to participate in any palaeontology research fieldwork organised by a person other than yourself?

Tick all that apply.

	Lead by a person within my own institution	Lead by a person outside my own institution	Open-application trip
**Yes, and I successfully did so**	☐	☐	☐
**Yes, but I was not successful in doing so**	☐	☐	☐
**No, I have not**	☐	☐	☐

15. Question 11: In the last 3 years, have you attempted to organise any paleontology fieldwork for yourself and/or others?

Tick all that apply.

	Solo fieldwork	Fieldwork with others from my institution	Fieldwork including others from outside my institution (including collaboration with local researchers)
**Yes, and I successfully did so**	☐	☐	☐
**Yes, but I was not successful**	☐	☐	☐
**No, I have not**	☐	☐	☐

16. Question 12: If you have attempted to participate in or organise fieldwork in the last 3 years, what barriers have you encountered in doing so? Select all that apply. If you have not encountered any barriers, select 'None'.

Tick all that apply.

- [ ] Turned down for research funding to participate in a trip
- [ ] Turned down for research funding to instigate a trip
- [ ] Lack of enthusiasm from colleagues or logistical support from institution
- [ ] Concern regarding the ethics of fieldwork and fossil collecting
- [ ] Locations deemed dangerous for travel
- [ ] Lack of professional contacts in the fieldwork target region
- [ ] Protectionism over fieldwork locations (i.e. sites 'claimed' by other palaeontologists)
- [ ] Protectionism over fieldwork trips (i.e. organised trip does not accept external applications or volunteers)
- [ ] Protectionism over data (i.e. researchers not wishing to share credit for their discoveries)
- [ ] Put off by the administrative burden
- [ ] Lack of information regarding the legalities of fossil collecting in the target area
- [ ] Personal circumstances (e.g. ill health, family responsibilities)
- [ ] Requirement for accessibility adjustments
- [ ] Lack of personal or professional confidence
- [ ] Insufficient training in field skills
- [ ] None
- [ ] Discrimination (this will be addressed in more detail in question 14)
- [ ] Other: ______________________

17. Question 13: If you have not attempted to participate in or organise palaeontology fieldwork in the last 3 years, please tell us why.

18. Question 14: Have you experienced or perceived any form of discrimination against yourself or others during your search for or experiences of palaeontology fieldwork, on the basis of the following characteristics? Select all that apply.

Tick all that apply.

- [ ] Ethnicity
- [ ] Gender
- [ ] Sexual orientation
- [ ] Pregnancy or parental leave
- [ ] Religion
- [ ] Professional mode of work (e.g. part time work, remote work etc.)
- [ ] Professional colleague relationships within your institution (i.e. who you have or have not worked with previously, intradepartmental conflicts)
- [ ] Professional colleague relationships outside your institution (i.e. who you have or have not worked with previously, inter-institutional conflicts)
- [ ] Age
- [ ] Disability or requirement for accessibility accommodations
- [ ] No discrimination encountered
- [ ] Prefer not to say
- [ ] Other: ______________________

19. Question 15: Do you have any concerns regarding the the ethical aspects of palaeontology fieldwork? Please give as much detail as you are able.

20. Question 16: Have you personally encountered any unethical or ethically dubious palaeontology fieldwork practices in the last 3 years? Please give as much detail as you are able, without naming any individuals involved, or including any other identifiers. If you are able, note whether the incident was reported to the relevant professional body or not.

21. Question 17: Please use this question to tell us anything else about your experiences around palaeontology fieldwork, and any suggestions you have for improving the approach to fieldwork by the field of Palaeontology.

22. Question 18: What is your age category?

Mark only one oval.

- <25 years old
- 26-30 years old
- 31-35 years old
- 36-40 years old
- >41 years old

23. Question 19: What is your gender identity?

24. Question 20: In what country is your academic institution? If you have worked or studied in multiple countries over the last 3 years, please provide list.

This content is neither created nor endorsed by Google.

Google Forms
