# Supplementary material for: #Yourpalaeolife: Interrogating the Status of Fieldwork Among Early Career Palaeontology Researchers
Source: Ecol Evol. 2026 Jul 29;16(8):e74032. doi: 10.1002/ece3.74032 (PMC13420382; doi:10.1002/ece3.74032)
Supplement: Supplementary file 3 — Data S3: ece374032‐sup‐0003‐Supinfo3.zip. [file ECE3-16-e74032-s001.zip › D3 Open question ethical concerns SI.docx]

I think that land rights and permitting are extremely important, but I also think that considering the opinions and beliefs of indigenous people who historically lived on the land you are collecting fossils on is important. For example, I do most of my fieldwork on BLM land in the US, and although we have the proper permits and are abiding by the laws, I don't feel like just doing a land acknowledgement in the acknowledgements section of a paper cuts it. I think paleontology as a field needs to reckon with the fact that we are doing fieldwork on land that is "empty" because people were forcibly removed from these lands, and I think that most paleontologists don't think about these things. I also think that although this is improving, there needs to be a much greater focus on safety and well being of those doing fieldwork over productivity. Fieldwork can be seriously dangerous, and I think a lot of field crews don't take that as seriously as they should, or they may seem to take it seriously on paper, but then in practice encourage unsafe field practices.

Ignorance, superficiality and maybe arrogance bring with them ethical issues. You must inform yourself on the rules and laws of the area where your choosen sampling location is. If it is inside a national park or a protected area, you must ask for the relevant permission to sample and, in case of doubt, consult the relevant authorities. You have to inform yourself on the local rules regarding handling fossils. For example, in Italy, macrofossils are equal to archaeological finds and subject to the same protection law; while Myanmar has very strict laws on the fossil ambers. If you don't know something, it is very fine to ask the relevant people and authorities, usually they are very happy to answer to your doubts. Sampling ethically and consciuosly permit to avoid a lot of headaches, complications and also legal issues that could be easily sorted before the sampling and permit to plan it more efficiently.

Collecting for the prestige, for credit, or to "claim" a site without any real thought to the scientific reason why the fossils are being collected--researchers need to put more time into considering the financial, environmental, and administrative burdens of field collecting before doing so. Collecting fossils to preserve them for some future researcher is a great goal, if the specimens warrant it, but collecting fossils just so that your institution can have the "biggest", "coolest", or just the most xyz taxon is unethical and we should push folks to stop this kind of trophy hunting mentality. More broadly, there are well established ethical concerns about commercial collecting (not to paint all commercial operations with the same brush, but as a broad generalization), i.e., commercial operations doing shoddy work and keeping potentially scientifically important specimens in private hands.

Yes. People are very protective of their sites and work. Sometimes this is because of their ego's, which is a them problem and is actually the rarest form of "resource guarding" I've seen in the field so far. The most common reason I've encountered is trust. Many scientist have been stolen from/or abused when they were up and coming professionals in their respective fields. Perpetrators are supervisors, well-known collaborators or fellow colleagues 'trying to get ahead'. This has bread a general sense of distrust in some and so they become very selective over whom they work with and share data with and earning those people's trust is a process you need to be willing to invest in for the long haul. That is sometimes an intimidating though if you are trying to picture your future in the field. Not something unique to our field alone though.

My concerns regarding the ethical aspects of the paleo fieldwork are from other category. The biggest problem, from my point of view, is not the lack of opportunities but the legislation: the laws aimed at protecting fossils, sites or areas of interest are so old and with such complex bureaucracy (where they exist) that it is ignored by everyone, including the authorities. There is no real legislation for collecting fossils (protected sites, national parks or mining areas) for professionals or amateurs. There is no legislation for selling fossils, for taking them out of the country or for restoring sites. So neither institutions nor researchers are interested in addressing this topic. Is it the fault of the local administration and the lack of legislation? Of course. Are the researchers taking advantage of this? Absolutely.

You're often stuck in the middle of nowhere with severe power imbalances regardless of whether the work is being conducted through a museum, university, or another entity. Gender and racial disparities always exist. Excessive alcohol consumption is normalized. This creates potential for a lot of outright illegal activity (e.g., sexual harassment) or behaviour that is not illegal but probably would not be condoned or permitted in a "typical" workplace setting with coworkers (e.g., the number of non-student personnel I have seen have "summer flings" is...not low). It can also result in isolating experiences for people who do not consume alcohol or have had previous bad experiences with it (e.g., family history of alcoholism) or who do not belong to majority demographics.

Yes absolutely. There is an argument to be made for the fossils of Australia belonging to the first nations people to an extent, or to the land itself as is true in much of their beliefs. To take things from that land without their knowledge or permission feels wrong because some would argue your taking a part of their identity. At the same time I do not think not taking things is the answer, I think collaboration, communication, consent and transparency are the answer. We can do palaeo work without stealing, hiding or lying. Even if it takes time. I also do think funding would help with this. Not to pay people to take the fossils but to support the engagement of people outside universities and to promote science communication of institutions more broadly.

With reference to question 14, the women in my department sometimes experience trouble in attending fieldtrips as it is stated by our institute that at least 2 people of each gender (male/female) are on a trip. I.e., if there are 2 men and 1 woman on a trip, the institute would stop the women from going on the basis of preventing any possible sexual harassment. Whilst this is understandable, it hinders the progress of female researchers if they are not able to attend field trips in what is mostly a male dominated field of research. I was fortunate enough in that my supervisor (male) insisted that the institute allow me to attend the trip, for which I was very grateful as I learnt a lot of valuable skills.

It is something I pay attention to, but have never had problems with, directly or indirectly. I go to the field almost every month with people who are both very experienced in field work and people who have had almost no field experience. From all genders, age, geographical provenance, professional background, different institutions, and I have to say I have never seen misconduct on the part of anyone. If anything, I notice the field work brings us all closer together. Traditional class barriers are broken and we become colleages. The emeritus professor and the entry-level student, all collaborating as a team.

Yes. I am a strong believer that we need to end the practice of parachute science, and I know a lot of fieldwork is still conducted with little to no input from people with local expertise. There are also both legal and ethical issues around the export and sale of fossils which are sometimes ignored for scientific purposes. Especially when joining fieldwork organised by someone else, I think you are at the mercy of their plans, and therefore ethics, so it would be difficult to speak up if you signed up for a trip only to find out that you would be complicit in questionable or illegal practices.

It depends who is participating on the fieldwork trip. There's still a lot of 'difficult' people in academia that can make an experience miserable with discriminatory or derogatory comments. And it can be difficult to execute fieldwork without certain committee members or find others willing to go with you. Thankfully, I never experienced this myself to a large extent. But, ethically, it would be great for access to resources so students know what would be good to know beforehand, how to tackle certain aspects, and available trainings (all beyond what you know from your own lab group).

Of course! As one interesting anecdote, at a conference recently, an audience member asked about the ethics of destructive sampling of the shells and otoliths with which I work. As these fossils are not charismatic (in the typical sense!), and because they are very abundant in the regions where I work, I had not thought seriously about this question--other than, I always work with the collections managers to ensure sufficient material for future researchers. I had not considered the intrinsic ethics of destroying the samples for the sake of the samples themselves.

As I wrote earlier, discrimination during fieldwork is sadly more common than many would think. I have only heard about it from others who have either experienced it themselves or were in fieldwork campaigns and witnessed something, and it includes gender and sexual orientation-based discrimination as well as physique-based forms of discrimination (e.g. overweight). But I'm afraid I cannot speak much about it since personally I've been lucky enough to have never been in such hostile fieldwork environment. I will leave this for others to share their experiences

I have not directly run into this issue, but I have heard from colleagues that researchers outside my main region of work have been known to collect and study specimens without working with the local palaeontologists/researchers. In some cases this is illegal, not to mention unethical. This sort of pseudo-colonial approach to science allows wealthier provinces and institutions to benefit from the material of other places without providing anything in return. It is unacceptable, and is an issue that needs to be addressed ASAP.

Many colleagues try to go as far as possible or at least as remote as possible, often in less developed countries, without a good support for paleontology locally and I feel it's neocolonialist. Sometimes they try to include local colleagues but they kind of exploit the countries that have potential for big discoveries, where no one is working because they don't have funding. Legally the fossils come back to their country after being studied, but all the glory is still credited to the "Old World"...

Currently unable to share a lot of detail due to time constraints, but I do have concerns regarding the competitiveness (sometimes related to a false sense of ownership) that can exist over collecting fossils from certain sites and/or regions. I also have some concerns with the way many attempt to bypass ‘parachute science’ by purchasing fossils from local collectors and then stating that the work was done with locals, but in reality this does nothing towards improving equitability in the field

If the matter is the ethics of fossil excavation itself: yes when it comes to international campaigns, but I only ever did fieldwork in my home country and residing country for which I have minimal concern and good knowledge of the legal statues of fossils. When it comes of the ethics of students in the field, aside from often not mentioning students as fossil collectors, we know and share information about people with known inappropriate behaviour in the field (often time misogyny).

I would like to see more involvement with indigenous communities in order to learn from and respect their long cultural histories with the lands on which we conduct fieldwork. I would also like to see fieldwork more broadly accessible to people who might need accommodations outside of the usual planning for fieldwork (e.g., how to make paleontological fieldwork more accessible to people with disabilities).

Not an "ethical" concern per se but I do feel a large amount of funding goes to fieldwork that could go to supporting professional careers. There is no shortage of understudied fossil material in collections worldwide as it is after all but few permanent positions to study them. This is not to say there is anything wrong with fieldwork in and of itself. Rather just a matter of prioritization.

I am concerned about the ethics of some institutions conducting palaeontological fieldwork outside of their own country without involving or enriching local communities where they work. I am concerned that this sort of fieldwork removes fossils from their country of origin with no resolve to return them or to involve local institutions or palaeontologists.

Local laws and customs should be respected at all times. If excavating or collecting on land that is owned by, or important/sacred/ancestral, to others, open and clear communication is paramount. Parachute science and colonial practices should be avoided. No is no, in all circumstances, and science does not go before everything else at all costs.

I am lucky to work in a lab with a lot representation (ethnically, gender, and sexual orientation), however I know this is not the case everywhere. I think in places where there is less representation things can be more insular and forms of toxic culture tend to have less checks and balances in terms of what is considered appropriate treatment.

What has bothered me most during my years studying Paleontology is the way people think women aren't capable of doing "hard work." When in reality, as I observe in the field of micropaleontology, they can do the same as men and even be more meticulous in describing outcrops and carefully handling, identifying, and collecting samples.

Depending on countries, the legislation can be quite difficult to understand. Starting a field campaign by myself could be quite challenging when the legislation is blurry... Who do I should contact? Which permits are necessary? Sometimes even people that worked many years in an area don´t know!

Big concern about parachute science, it is still common to do fieldwork in lower-income countries without including local researchers. Another major and perhaps even more important is the unavailability of resulting publications to the population (mostly because they don't speak English).

Yes, I feel conflicted as a white paleontologist from a western institution who has collected fossils (and brought them back to the US) and benefited from fieldwork in a country where there are fewer educational resources available (due to colonialism) to train local paleontologists.

There is always the question of who owns the data. Unfortunately, ownership of data depends on its quality, as some researchers or professionals believe they deserve the most ‘beautiful’ fossils, which end up on a shelf instead of in a collection available for research.

the permitting process can be extremely burdensome in certain locales, which leads to either temptation to collect without a permit, or not being able to collect. likewise it is sometimes unclear whether a permit is required and who to ask about getting one.

Our research group strives to make as many local connections as possible within the communities we work in, and this has had a tremendous impact on both the researchers and the communities. I do feel that this is not the norm, unfortunately.

Yes, plenty around who is doing the fieldwork, how extractive it is, and how stakeholders/caretakers of land are impacted. Particularly parachute science by the west into the global south and east and violating indigenous landowners

The fieldwork I have been involved in was lead by a research group from the country the fieldwork was conducted in. I would have concerns about undertaking fieldwork in a country with no or minimal involvement from local scientists.

I feel like we are taught very little about ethical considerations in palaeontology fieldwork. Everything I know is through informal conversations with colleagues and supervisors, and has never been a part of any formal training.

I don't think this issue is addressed enough in academic training with field work. It was mentioned in my undergraduate courses, but not enough. There should be more clear guidelines established for ethical field work.

No point collecting fossils that are not at risk if they are not being studied. Ethical concerns around the lack of engagement with Traditional Owners - this is improving in Australia, but still has a long way to go.

Some old boys club mentality there as is elsewhere, too. Have had some great experiences but also some where male older postdocs or professors either looked down on female students or hit on them. Not great.

Nothing immediately comes to mind - every site is its own consideration with normal common sense concerns about local laws, land ownership, disruption to the are, claims to the fossils and so on.

Scientific imperialism is a concern for me, though as I type out I feel like I'm rambling without much sense. It's a topic I think I need to do further research on to put my thoughts into words.

It will be regionally dependant, but the perceived ownership of research areas by one institution over another creates a conflict of interest that can hamper early career palaeontologists.

I don't have time to answer this as there's a million things to discuss here? This is the subject of whole careers, so I'm not sure it's something that can be answered in a form like this.

Concerned about proper permissions and cooperation with indigenous elders (Traditional Owners) not taking place in fieldwork that has been ongoing at a site for over a decade or so.

I have personally experienced, and I know of others who have experienced, sexual harassment during fieldwork. Unfortunately, there seem to be many similar cases in palaeontology.

During the fieldwork, paleontology is unknown or ignored by the local population and to take off some spécimens make them unhappy like taking off the ancestors' possession .

I do have some concerns, primarily regarding helicopter palaeontology and poor quality collaboration between local scientists/residents and visiting researchers.

I do not have any concerns. The findings should be conserved, documented and published online for anyone interested to access in order to promote free science.

It appears that most sites that are regularly worked are presided over by specific individuals, which often leads to unethical pravtices and gatekeeping.

No, as long as this topic has been discussed and agreed prior to the excavation. It is not something to do during the excavation.

When local collaborators are not invited to be involved in the publication of research derived from fossils in their region

Yes, travelling to other countries for fieldwork but not being directly involved with researchers from that country.

I haven't come in contact with these concerns yet, but I am aware that there are many debates about it.

Not enough formation on that and the laws about fossil protection (at least in my country France)

Yes. Sometimes some researchers are too competitive for materials and access to collections

Unfortunately, the institutions only care about archaeology and NOT about paleontology

Regrettably, safety is an aspect rarely taken into account in my country

Insufficient legal protection of scientifically important fossil sites

Yes. How do universities enforce inclusivity norms in fieldwork.

Not in the regions that I have been previoulsy working in.

None if it is done properly and following good practices.

If a journal prohibits, I will concern

This is a literal mine field

I can't say if there was

Illegale sale of fossils

Not usually.

See Q. 16

none

No.

Yes
