# Supplementary material for: #Yourpalaeolife: Interrogating the Status of Fieldwork Among Early Career Palaeontology Researchers
Source: Ecol Evol. 2026 Jul 29;16(8):e74032. doi: 10.1002/ece3.74032 (PMC13420382; doi:10.1002/ece3.74032)
Supplement: Supplementary file 3 — Data S3: ece374032‐sup‐0003‐Supinfo3.zip. [file ECE3-16-e74032-s001.zip › D4 Open question field skills comments and suggestions SI.docx]

I went to undergrad in an American state school with a dedicated Geology program, but a small one. While the capstone course was a well-funded field camp, cramming all the field techniques into a month or so did not help with retention. The department highly encouraged students to get field experience by taking on research in addition to their class load, and everyone I knew who was successful in the program did so. In turn, though, many became overwhelmed with their course load because of it. I was very fortunate that A. I was encouraged to reach out to the local museum's paleontology curator by a friend, and B. Said curator was well-versed in the department's research for credit system. I was able to get lots of practical field experience and I had time to do so because my research replaced upper level classes. Most of my classmates were not aware of this option, though, and struggled with workload. In short, my school had good field work resources but did an awful job communicating and streamlining those systems. A brief mention of my PhD program in China: I arrived in 2019, just in time for travel to shut down. While we had planned field work, it did not happen. I think it may have been for the best, as by that time I knew I preferred lab work and I got even more time honing those skills. This affects question 10 as no field work opportunities were available through my institution for exceptional reasons.

All of my knowledge around field trips and skills came from my undergraduate course. This included theory and practical experience with mapping, sampling, interpretation etc. How to read a cliff face essentially, and especially how to take comprehensible notes. This was all very clear and good quality training, but I've not experienced formal field work outside of that - the closest would be personal fossil hunting trips for leisure. It would help if there was some way to know, besides tea room gossip, if anybody was planning to run some field work, or where to find further training. Generally my biggest barrier is that if I want practical experience around the formal ins and outs of running a fieldwork excursion I need to be in some in-group that's already running it. Searching now, I can find places like BGS running some kinds of basic field training, but during my PhD these kind of resources have not been signposted to me previously. I'd have no idea where to look for anything beyond that. Even just an online list aggregating links to palaeo and geoscience training providers (maybe a way to review them, too...) would make some difference. It'd also be nice to have a place (besides mainstream social media) where individual researchers interested in fieldwork in a specific region can coordinate together to run joint trips together - perhaps a dedicated forum?

I have a good experience in prospecting, digging, microfossil sampling (screen-washing and picking), palynological sampling and logistics. I don’t want to place the blame on the students, but I believe that many of them show a lack of motivation when it comes to fieldwork. They often don’t seem to recognize how essential it is for their professional development, and as a result, they don’t make it a priority in their schedules. In my country, access to volunteer opportunities is quite open and straightforward, yet many students create their own barriers to participation. While it’s true that more specialized training requires pursuing a master’s program, the first step toward building practical skills is often through volunteering and hands-on experience. Unfortunately, many students miss these opportunities, either because they are uncomfortable with field conditions (such as camping, distance, or limited amenities) or because they struggle to balance fieldwork with other commitments. Maybe participating in low-commitment experiences would help students overcome their initial hesitation.

In the United States, I think many palaeontologists gain fieldwork expertise through professional experiences other than their education; I am hardly the only one who started out volunteering with a museum and accruing significant skillsets that I would never have learned in formal coursework or educational training (many universities do not have palaeontology programmes). However, this can be a dual-edged sword for improving access because some people have access to tremendous opportunities that others do not simply by happenstance of where they live. Most students will not be able to afford one of those "pay-to-dig" experiences, which are often too brief to really provide comprehensive or even basic training per se.

Regarding Q8, while i have not faced outward discrimination I was also not provided any kind of support when difficulties arised from my chronic disease. During my master especially, most of the support and help I got was from some of the other students but never from the professor who generally disregarded problems as something people have to individually deal with. This problem extend however beyond the scope of just field word and was especially troublesome during the pandemic which happened during my master years. Same issue with students who got their periods in the field, accommodations such as portable toilets was often not considered.

In my experience the problem isn't training or barriers, it's the rarity of fieldwork itself. To some extent there is a level of discrimination I suppose because when rare field trips do appear, those with a higher level of experience and expertise can be prioritised, and beyond that the people at the institution may be prioritised more than outside students or researchers. That's just how it is. More funding and more fieldwork would open up the possibility of more training and more spots to engage. Though overall my insitution does a great job of building skills from the undergrads up.

I never got formal fieldwork training. Even for my PhD, going into the field for a week at a time and organizing students to help, acquiring necessary equipment, etc., if it weren't for my highly organized personality, it could have been a big failure. My advisor never taught me how to choose sites or best practices (e.g., I wish I had recorded more lat/long coordinates), and barely helped me prepare for fieldwork. Overall, it was a success, but knowing certain things beforehand would have made the experience better as well as the quality of our science.

Field work training (in my experience) does not seem to be standardised, but it definitely needs to have standards that are met. Most of the time, those who go on field work is dependent on who relates to the subject of matter (e.g., what deposit is being worked on), but field work competence, safety or strategy is not as much of a priority and/or non-essential. A degree of competence in the latter should absolutely be enforced for any participants.

I believe this is a tough question to answer as I find there have been very few if any times I was formally trained. Any formal training came from my summer job rather than my academic career. Training has either been field courses during my undergrad or when out in the field I had experts teach me the ins and outs of field work. I have been in the field many times including very remote locations and most of the training was in the moment.

My experience is that when field trips are organised, the organisers rarely take into account the life experiences, lack of opportunities or cultural differences and many times people who are at the intersection of these factors, finding themselves slowly squeezed out of field training. No attempts are made to normalise the field competencies across these differences.

I think there needs to be a formal fieldwork program which PhD students can access for multiple field seasons. This is how the system works in other countries, someone will return again and again to the same fieldwork site as a student (including as a masters student) which allows them to gain skills and confidence and gradually take on leadership roles.

During my studies at the University, we had several field practices in different regions of our country and in different geological periods. In the senior years of study, each student individually underwent work practice with scientific researchers with a specific focus on a certain group of animals and the geological period that the student is studying.

I am grateful to my colleagues and friends outside of my institution who took the time to recommend key sources to read and review prior to fieldwork, who led me through fossil collecting and field techniques, etc which have resulted in published papers and posters. I am currently now working on my portfolio for a future PhD by Publication.

Finding field experience outside of my working lab group run by my supervisor is difficult. Many other labs have limited resources and they prioritize the members within their labs. This means that field experience is often limited to one group of people and one set of localities. Which is a start but I want more! I'm just being greedy...

I feel that my fieldwork experience prior to PhD was general but nonetheless very helpful during my PhD fieldwork. If there was a deficiency in my experience that became apparent during my PhD it was analytical - I felt that I was lacking in analytical knowledge and methods that others had, particularly with regards to using R.

The biggest hurdle I encountered was the pandemic, which impacted all undergraduates at that point (at least at my faculty). This mainly caused me to only experience local small-scale excavations, instead of participating in international trips to excavate in different environments.

I have never received formal training in the literal sense, but all of the skills that I have learned originally came from participating in fieldwork and watching or being shown how to do things by the leaders of that fieldwork and/or other participants.

In paleo people expect that you know your way around. Not knowing things feels rather frowned upon so people don’t ask and therefore people don’t tell. Now I know better but as a PhD student I was too intimidated to ask for basic field work techniques

Broader advertising to graduate students would be a start. There are disproportionately more undergrads and it may not be feasible to provide opportunities to all of them, but grad students are in need of these experiences and should be prioritized.

I have encountered sexist language from supervisors when teaching palaeontological skills to a mostly female student group, wherein the supervisor suggested that women were weaker and less able to do paleontological and geological work than men.

During most USA-based PhDs, we are obligated to take a semester-long course in ethics of science. My PhD was not in paleontology (it was in Geosciences), but some of the aforementioned training opportunities could be included in such a course?

I once had the opportunity to do field work, but the responsible professor would not pay for travel expenses (which were quite expensive) despite having funding and urged us to pay for it ourselves. I didn't take that opportunity, obviously.

As a man I have not experienced discrimination, however I have secondarily seen the "muchismo" culture than can sometimes pervade field camps that certainly made some female and people of different fitness levels feel uncomfortable.

I would suggest having more field trips and dedicating more time to each of the categories mentioned. My experience has been quite limited, only about 4 field trips, and the material we covered felt rushed and crammed together.

It would be great to have a formal master course dedicated to fieldwork practices. They exists but are very restricted in the number of students able to follow them and are generally concerning invertebrates.

Since I mainly worked on collections material, I had very few chances to do fieldwork, mainly in the form of brief excursions within bachelor/master courses or a brief fieldwork expedition during my Ph.D.

There does not seem to be much of a culture of incorporating paleontological field work and accompanying training into PhD programmes in the UK (compared to my perception of field work culture in the US)

If you join societies or associations related to paleontology/geology you will certainly find plenty of oportunities to go to the field with experienced professionals and learn from them.

The paleontology fieldwork that I've done during my PhD was a key moment to improve my confidence as a paleontologist. I would love to have more opportunities like that during my PhD.

My bachellor's and master's degree had field work and field trips included in the programming, some of them were mandatory, so there were not many barriers to access field training.

As suggestions, I think better advertising of the opportunities would be great, and developing means to provide financial aid for those interested but uncapable of affording it

I was lucky to be able to volunteer my time, unpaid, for excavations. Not everyone is, and more funded fieldtraining with open and advertised applications should be available.

It is important to have several options or days to visit specific dig sites. You might not be able to go because of reasons to one of them, but you might get other.

In my experience, field skills training via fieldtrips is rarely offered to PhD students, especially those with no or little previous experience.

It all boils down to funding, I don't think people have differing levels of access beyond simple differences in available funds

I've been lucky but have also worked my ass off to prove my worth and said please and yes to every possible opportunity.

We didn't have much training before the field, some formal classes, but most of the learning took place in the field.

The training i had in my undergraduate studies was basic and was provided by my supervisor but not by the institution

Researchers and students should look for non academic research institutions for training

funding to offer fieldwork which opens possibilities for in-field training and learning

For intuitional purpose, field trips were attended to see fossils as apart of course.

Increased funding and breaking certain stigmas for people with medical limitations.

It could be advertised via PaleoNet and offered some scholarship to attend

Try to win grants which offers workshops/field work applications

Mature aged woman, sometimes underestimated by male colleagues.

Reform of education system regarding paleontology teching

Small teams are more progressive

Motivtion ny YouTube and video

Be more flexible
