# Supplementary material for: #Yourpalaeolife: Interrogating the Status of Fieldwork Among Early Career Palaeontology Researchers
Source: Ecol Evol. 2026 Jul 29;16(8):e74032. doi: 10.1002/ece3.74032 (PMC13420382; doi:10.1002/ece3.74032)
Supplement: Supplementary file 3 — Data S3: ece374032‐sup‐0003‐Supinfo3.zip. [file ECE3-16-e74032-s001.zip › D5 Open question other comments or suggestions SI.docx]

I have not yet experienced any discrimination or barriers owing to pregnancy or parental leave, but I am currently pregnant and expect to do so. I do not feel able to organise or attend field work while pregnant because, for the most part, adequate health and safety is rarely taken into account in the field work I have attended to date. This has been a concern for me in the past when not pregnant and is more so now. For example, my field work is often in remote places that are very hot and dry, and so are far away from adequate healthcare, transportation, etc. We frequently have not had access to things like satellite phones for emergency. I have also had many vaccinations and other medical care that have been required for travel in the locations in which I have carried out field work (or conferences) - I have been informed these are not reimbursed, so this aspect of personal safety in unsafe conditions has been at my personal cost (which encourages unsafe behaviour!). I have also had several situations in which I have travelled to places in which I feel unsafe for field work. For example, carrying out fieldwork in a remote place when all the men in the group moved to another area for the day, without consideration of the fact that they would be leaving two women alone in a geographical location where it is not particularly safe to be on your own as a woman. Additionally, I have struggled through much of my research career with accommodations during field work for chronic fatigue. Field work, in my experience, is full-on, physical, often sharing rooms (so no alone time), with lots of drinking. Even when I have clearly expressed my needs to be able to have some rest time and a reasonable nights' sleep (and other logistical aspects like a rough daily schedule of leaving time and returning time), these are very rarely accommodated and have put me in difficult 'defensive' situations, even causing souring of relationships with colleagues because they feel constrained and/or insulted by a schedule and something akin to 'quiet hours'. Many people still have the attitude that field work is some kind of holiday, and they should be allowed to operate on little to no sleep and high levels of drinking (even when affecting their work the next day). This is not an inclusive attitude in any way, and does not facilitate those with any need for adjustment. Some of these experiences have even been while I was more senior on the field work (not a student), and have led to me reconsidering future professional relationships. However, these viewpoints and experiences have been also with early career researchers - it is not just senior researchers that have these types of 'party' attitudes towards field work. Apologies that I don't have any concrete suggestions for improvement here! I personally feel that much of the field needs an 'attitude adjustment' relating to field work, and this can probably only come by far more compulsory training than currently carried out.

For me, it's a big ask to go out somewhere remote, potentially somewhere dangerous, with people I don't know very well. There are so many risks involved, and I would have to really trust the organisers on so many counts. Will we be physically safe? If I complain about something, will I be respected and taken seriously? If someone behaves inappropriately, will they be removed? In truth, for a lot of people I _do_ know who do fieldwork, I would not have faith that they would give me the answer I wanted to all of these questions. I have a strong perception that if you drew a Venn diagram of "palaeontologists who do fieldwork" and "palaeontologists who are horrible/inethical people", the amount of crossover there is huge. I think palaeontology is competitive, and the fieldwork side even more so, and this means that you select for the worst people in this subfield. It's especially unfortunate that this results in bad people having control in high-stakes circumstances. Negative experiences can quickly fix your perception too. I remember, as a first year undergraduate, drawing rock outcrops as snow fell on us. I was so cold, and didn't feel like I was learning much because we couldn't see that well in the snow anyway. It left me with such a strong impression that fieldwork plans are stuck to, no matter the circumstances, including the weather. I figured that meant fieldwork wasn't for me, I was too 'weak' to put up with whatever the outdoors threw at me. Looking back now, I think I just hadn't been given enough information to be properly prepared, and this would have made a world of difference to how I felt in the moment, and my attitude to fieldwork over the following years. I did demonstrate on undergraduate field teaching as a PhD student - and I saw the other side of the coin, enjoying it much more because I had more autonomy and less to do, I could move around if I was cold, or find someone to talk to if I was bored. To compensate for this though, I ended up being given more pastoral responsibility because I was a woman (and we were short of those in the staff team). In a scenario where I had the skills to do so, if I could plan and conduct my own fieldwork (ie be in charge), I think I would probably enjoy it, and do a pretty good job too. Ethics/EDI are generally important to me, so I think I would be attentive to everything that needed considering, including any other team members. But the path to achieving that is completely innavigable - thankfully I am in a position to not need to attempt this, but it does make me sad that these are skills that I will probably never gain.

I think trainings and courses in field safety and field ethics through universities for graduate students should be required for anyone planning their own fieldwork, and optional for those participating in fieldwork. I think making these open, or even required, for professors would also be great. I took a wilderness first aid course recently that was open to grad students and professors and it was great and extremely helpful, but I wish everyone in my department doing fieldwork had taken it. Also, during the course, we asked a lot of questions regarding planning and managing field crews and field courses with as many as 20+ participants, and I think that having courses that are entirely focused on field safety planning would be extremely useful. Not just running first aid scenarios, but how to plan fieldwork to prevent these things from happening in the first place. There is a field ethics seminar at my university that I've heard other students took, but I didn't have room in my schedule to take. I think making opportunities like these more widely available, and making it so they fulfill graduation requirements (so that students can prioritize taking them) would be extremely helpful.

Where I am, field work is prized and people in our department are invited to go if they have relevance to it. For those that can go, these rewards are a bit like holidays away from the lab. This somewhat becomes frustrating though when the same people are invited to go, time and time again, to the exclusion of others, and it is taken for granted (that is, if their work almost integral to the lab--by chance). Some may be excluded because they are considered to be busy with other aspects of their work, but this is not articulated to them. While them being to busy is a valid reason not to go, the secrecy combined with the action creates a perspective where those who are working longer hours are effectively not rewarded for their efforts. A main problem underlying all of this is that field trip leaders or academic lab supervisors are not often trained in management, and so do not consider any of these things. They got to these positions via academic achievement which does not necessarily translate to managing and dealing with people well.

More funding for fieldwork of course. But more than that, more funding for work surrounding this topic as well. Funding for people to be paid to process, identify, categorise and store material. Funding to publish not just in scientific journals but in open communications available to the public to allow for their understanding and involvement where possible. Funding to cast things and cast things and make it available to more people and more research. Bones aren't doing much good sitting in a museum. Fossils should be shared for research and shared for fun and while proper storage is important, casting and new technologies around 3D modelling are a great way to share these things! But this all takes time and so all needs money. I genuinely believe there are tons of people who would love to do the work and that this would be a great thing to share with the world, but money makes the world go round.

I feel like I'm discouraged from initiating fieldwork, as if it were a distraction from 'real' paleontological work. I believe even simple checklists of what is known from a site and at what levels are well worth the effort spent; in fact I think most people agree we need to keep collecting data, but it's not encouraged. We either need random wealthy people with nothing to do to get out and obsessively document beetles and shells and such again, or lacking that, perhaps make it easy for smaller groups or individuals to opportunistically go out and collect. If you're going somewhere to dig for a week, I'd like it to be more normal to encourage others to tag along - we don't have to facilitate another person's work, but the organizational load feels a lot less if you're travelling with others.

I think every palaeontologist must participate to field work to improve their skills and better understand the geological context where their samples come from. It would be auspicalble that also students and general interested public have the possibility to participate in not very challenging locations because it is a great opportunity to divulgate science and make a significant scientific experience. It would be also a great idea to create a global database where field information (Log, map, general characteristics, main geological formations, fossil founded) is stored. This will help the information sharing, the planning of future field samplings and the creation of new international collaborations.

My main thought is that young people don't have a clear pathway to gaining field skills. Most institutions have roads in place for them, but leave it up to self-motivated students to seek them out. I suspect this may be purposeful so as to keep systems with limited funding from overloading. Once graduated, field work becomes much more difficult to participate in due to economic factors. Most of the world requires a lion's share of one's time spent on work to attain basic necessities, and field work is only doable over a weekend in very specific places. And, of course, Covid made sure no fieldwork happened in China and other highly-ffected areas during its height.

My experience of palaeontological fieldwork has been largely positive. The main issues I find are the poor advertising of fieldwork and training opportunities and the lack of fieldwork opportunities unless you are a doctoral or senior researcher (particularly in the UK). I attended a palaeontological field school on the Isle of Wight in 2022 during my penultimate year of university, aimed at undergraduates, which provided most of my field skills prior to starting my PhD. My suggestion would be more undergraduate opportunities specifically in palaeontological fieldwork and skills training.

The biggest issue I have faced with fieldwork is opportunity, costs, and time. Opportunities are infrequent, especially since I do not conduct fieldwork for my own PhD. The times I have had the change to conduct fieldwork for other projects have been very costly and I have severe financial restraints as a PhD student. Lastly, time is a major factor, as a PhD student you are expected to work on your project for the entirety of your degree. Taking several months to participate in fieldwork for someone else's project can be discouraged by your supervisors.

There is a consistent lack of attention to health, safety, and hygiene standards during palaeontological fieldwork. This issue particularly affects people with chronic conditions, such as those who require daily medication, and also those who menstruate. In many cases, hygiene and safety provisions for these participants are minimal or completely absent. Moreover, having such needs is often perceived as a disadvantage when applying to join field teams, which creates additional barriers and exclusion within the discipline.

Despite the fact that I have personally never experienced any discrimination or barriers to my participation in, or organizing of, fieldwork, I think there is a general sense of antiquity around the process that might feel like it's hard to break into. There are a lot of senior researchers in the field who run things the way they have always been run, and aren't necessarily receptive to changing with the times, especially when it comes to things like health and safety, and inclusivity.

I feel that all researchers organising fieldwork should make a designated space on their teams for an open-access volunteer external collaborator in an effort to spread experience and field skills further among early career researchers and promote good relations between institutions. I find that the attitude of many senior palaeontologists is ego-driven and competitive and I believe this is stifling scientific endeavour and the development of junior researchers.

It's a bit too bad that your questions only focus on the "negative aspects" : ‘dubious practices’ , ‘forms of discrimination’ etc. Field experience also has many positive aspects other than ‘field skills’ that are not mentioned in your questionnaire (networking, developing secondary languages during international fieldwork, discovering countries and cultures, etc.) and which would be worth addressing if you wanted to know about our full experiences.

PI promised to comp travel expenses, then refused later; this seems to me to be an assumption of financial means on my part that I simply don't possess, as PI was himself wealthy and seemed to not understand why this was a burden. PIs are often out of touch with what constitutes a financial burden for a graduate student, and when students are forced to pay for things out of pocket that can really frustrate their ability to conduct fieldwork.

Increasing the level of training and support provided to new hands on fieldwork before and during the trip. I have seen students fall apart during badly managed field work because they felt untrained and unsupported, which led to anxiety over doing something wrong. Provide a detailed field manual for each trip so everone knows what to expect. Mental health training for field trip leaders.

In the UK palaeontology funding and training seems to be shifting away from fieldwork even though it's foundational to the discipline. Although I don't believe it's essential for every palaeontologist to have fieldwork training the option must be there for those who want it. We need palaeontologists with a diverse range of skills to keep the discipline alive and flourishing.

I think it should be included more in the training of courses during bachelor/masters and even at the beginning of Ph.D, but I understand the logistic problems that may occur. Moreover, the fact that many paleontology research projects are collection-based implies that fieldwork is not necessary in that instance and often limited fundings should be used elsewhere.

My fieldwork experience in paleontology came with the challenges of each year of study, from undergraduate to PhD. I went and tried to go to the field as often and wherever I could, and I learned something from each outing. Unfortunately, I didn't learn how to approach fieldwork ethically but rather which areas to avoid because someone else is studying them.

My fieldwork experience over the last three years has always been quite solitary, involving only my thesis supervisors and myself. I have had no experience of fieldwork with other colleagues or of multidisciplinary research within a research group or research project. This is something I have missed, and it is largely due to conflicts within the department.

Collaborate with societies and associations as much as possible as it brings people from different places together. Not just the ones within an institution. This also improves the sharing of skills between specialties which might be useful in your specific type of study (e.g. using field skills from pristine areas into highly deformed areas, and viceversa).

I have only had very good experiences when I participated in fieldwork, all of them under the supervision of someone else. I think my only suggestion to improve it overall is to make the availability of opportunities a bit better announced and reassure the participants that they are going to a safe environment, i.e., discrimination-free

Have heard many stories about PI's/those organizing field work who expect everyone else to do everything, do not provide enough food, require people to pay their own way last minute because lack of funds. Formal training for those in charge would be helpful but I'm not sure they would take the training.

Sometimes I understand that improvisation is necessary in fieldwork, as unexpected problems arise... but you can´t be ALWAYS improvising with no permits, no plans or enough experienced people. If the excavation is something planned with time, please think about it with time and have a proper plan.

Senior researchers need to try and accommodate for more students to attend their field trips, or have participants on rotation (I.e., there are field trips that I would like to attend but they are already always full because the same researchers/students get invited each time)

I've checked that I have done paleontology fieldwork in the past 3 years but I want to clarify that it has been fieldwork collecting and culturing live organisms for insights into paleontology, not collecting fossils. (My previous work was more traditional paleo fieldwork.)

I think overall there is a push among up and coming academics to make fieldwork more accessible in people of all backgrounds and skillsets. I think representation and the creation of opportunities to do fieldwork is very important to growing the field in a safe way.

Fieldwork can be hard with no toilets for those who menstruate. Guidance on this in field training would be useful. It would help people to know what to expect more. I think this sort of stuff is often forgotten if people running the trips do not face these issues.

I have been actively conducting fieldwork every year for the past five years, participating in at least two expeditions annually, including international ones. So far, I have had the opportunity to work with two excellent teams without any issues.

Research goals should be open to public, especially at the institute giving out permits. If no conflict in research goals is present, and no history of vandalism or any other conflicts of sites, permit areas should not be arbitrarily limited.

I would like to suggest palaeontologists collecting fossils from or purchasing from locals collecting in regions lacking in sufficient support for maintaining fossil science and heritage should do more towards making the field more equitable

It needs to be taught as a proper subject. I learned just by doing and being in the field, just with "hands on" experience. I am able to operate professioally in an excavation site, but I am not able to have to deeper understanding of it.

Has not occurred personally, but i do think there needs to be more awareness/opportunities for women to be involved in long term fieldwork with things taken into account for their safety/needs throughout.

I have primary faced discrimination based on ethnicity, economic barriers and the ability to blend in well with cliques. Inclusivity needs to be emphasized in Paleontology.

I think we need to focus more as a field on including and giving back to the communities which we do fieldwork in, whether that be local to our institutions or abroad.

People need to advertise their field work and secure funding. I'm part of our paleontological association yet I have never seen field work advertised through it.

I am a coordinator of paleontology field work and management of laboratory, and we are are always open to have more students coming for field work with us.

My only experiences with paleontological fieldwork were during my undergrad and master's degree. All in all, my experiences have been very pleasant.

I enjoy to collect fossils and work in field. You should prefer to work in group because of ease in securing and transporting the specimens and

There should be a mandatory first day of teaching techniques and safety stuffs. Previous participants could revisit and revise or help out

We had some problems with the field site, with landowners blocking the research entrance, and some even coming to us armed.

To discover the fossiliferous'site, we show to the local that the fossils indicate the past life ( animal and vegetal)

More support for women in field work and more support for people in field work with disabilities or medical issues.

I attended a tracksite excavation as I was invited but it was not related to my project

Hypocrisy and empathy training to supervisors. Repercussions for PI's. No covering-up.

Discrimination against race has been a major issue, especially as woman.

More communication on social media on available fieldwork for students

A network in the UK where fieldwork opportunities can be advertised

Restore the natural relationship between life and earth sciences

need more efficient and less time consuming ways to get funding

Nothing else to had, due to my limited fieldwork experience

My experience has been quite positive

Information increases accessibility.

More fundings opportunities for ERC

No comment

No
