# Supplementary material for: #Yourpalaeolife: Interrogating the Status of Fieldwork Among Early Career Palaeontology Researchers
Source: Ecol Evol. 2026 Jul 29;16(8):e74032. doi: 10.1002/ece3.74032 (PMC13420382; doi:10.1002/ece3.74032)
Supplement: Supplementary file 3 — Data S3: ece374032‐sup‐0003‐Supinfo3.zip. [file ECE3-16-e74032-s001.zip › D6 Open question other field skills SI.docx]

No

None

People/community skills! When people are stuck in a remote corner of the world together for weeks, things can get a bit tense, so working with people is key, I think. 4-wheel driving is also good, and trailer training. Other skills fundamental for field work are basic camping ones (e.g., setting up tents, making a fire, cooking, willingness to be and work in a remote landscape), and a general interest in being involved, working together and taking responsibility. Nothing worse 3-weeks into field work than having a divided team because some aren't contributing. Mental health training and remote first aid is also essential, for trip leaders especially. Field work in remote and/or climatic/environmental extremes can push people to the limit, and be dangerous without any hospitals or towns nearby. Attendees need to be aware of this and be familiar with how to tackle this if/when the need arises.

Different methods of prospecting for fossils is a great skill to have. As well as basic knowledge of geology to assist in finding fossiliferous outcrops and assessing the stratigraphy of sites. I’ve had formal training on different prospecting techniques but nothing for the geology side. I also only had a brief introduction to constructing stratigraphic sections on sites and would like to have more training. I also attended a formal workshop on identifying dangerous snakes in the field and what to do in the case of a snake bite - this was very helpful and I think it should be something attended annually for anyone doing field work. Another course that should be done annually is a basic first aid course in the case of field injuries - I have not received any formal training for this topic but feel that it is important.

Communicating with non-scientists that you encounter in the field. This subject has only come up in conversations about field work, usually involving bad or uncomfortable interactions with people not understanding the work that scientists are doing on site. This should be covered in all field training courses, including preparation before leaving for the field to ensure all participants understand the local laws of the site and the potential interactions they may have with locals. I think consideration should be given towards how people from different backgrounds or ethnicities might be received at certain field sites, and training should be tailored for that and contingency/safety plans established beforehand.

I haven't participated in any specific training. However, I have a bachelor's, master's, and doctoral degree in Geology, always with a significant amount of fieldwork throughout my studies. During and after my doctorate, I had the opportunity to go on field trips with people who were collecting macrofossils (I'm in the field of Palynology), and I noticed that they don't have much of a basis for making log's to control sample collection at the outcrop. This is mandatory in micropaleontology. Good identification of sample bags is also essential. Always indicate the sample name next to the layer in the profile drawing.

Fieldwork often takes place while camping, often without amenities nearby. People who have no prior experience should receive an introduction before departure. No formal training needed, but an overlooked part for starting paleontologists from disadvantaged backgrounds. Could discourage people as well. Collection management while in the field is important as well; how do you deal with numerous finds over several days/weeks, organization-wise. Excavation mapping, how to set up a grid, how to interpolate coordinates, and how to reconstruct a map with finds from that.

During my master’s program, I completed several fieldwork courses, but even before that, I was already volunteering with a paleontological association, where I gained valuable practical experience. I believe that palynological and microfossil sampling is often overlooked by paleontologists who focus on macrofossils, even though it is a crucial tool for paleoecological reconstruction. Since 2021, we have incorporated this approach into all our fieldwork, and it has been yielding very promising results.

I find particularly important the identification of taphonomic processes and fossil identification (not taxonomical identification but stratigraphic position identification). I think these two issues are of high importance in field work because the information can only be taken in the filed, and not in the laboratory. I have not a formal training for them (I do not have done a course about it) but I consider that I have received good training from my supervisors during the field work.

Interpersonal skills. I work along roadcuts and it is necessary often for me to communicate with community members who stop and ask questions about my fieldwork (eg they see one to three people in safety vests and helmets and they have questions about what we are doing while we are measuring outcrops or collecting other data). This often also requires discussions with LEOs and engaging in cultural sensitivity esp with rural stakeholders who are suspicious of academic work.

For a site I have been too we had to do rope training due to the complicated nature of getting to the fossils within a cave system. We were specifically required to train for this rope ascent and descent prior to participating in that field trip. There were members of the crew that participated without this training but they could not go all the way to the fossil site, only help with transportation of gear and fossils.

Underwater paleontology - no formal training but applied in the field in freshwater/non-marine settings. Off-road driving - no formal training, applied in field. Operating devices for live feeds/social media/education and outreach from the field - no formal training, applied at remote/direct sunlight/hot field sites. Field site photography/photogrammetry - no formal training, not personally applied in field.

Hiking and prospecting are important components of fieldwork, particularly in the ability to hike far distances, identify potential localities, conduct preliminary examinations either individually or with a small team to establish the worth of opening up a quarry. I say this because many sites can be extensively remote and lack vehicle accessibility.

Communication with the public / interfacing with the community in which the fieldwork is being carried out. I had informal on-the-job training with this prior to my phd, purely by chance. I worked with a PI who placed a lot of emphasis on the importance of communicating and working with local stakeholders and landowners.

Planning & coordinating with other entities to get into the field (e.g., acquiring a boat and captain), permitting, Department liability forms, 12-passenger van driver safety training (this is the only formal training I received). Everything was learned from advice and learning by doing.

Every sites are different (bones vs tracks etc.) so important to know the differences and the different excavation approaches. But most importantly is to know the historical aspect of the field, whether you need prior agreements with the local (indigenous) community and work with them.

Driving, hiking, backpacking in different conditions, but informal training was effective and I haven't looked for formal training. I did get formal training in wilderness survival skills and wilderness first aid though, which is useful.

The only field skills taught were in numbered university courses, and training as a staff member at a palaeontological museum. More dedicated training provided by universities and supervisors would be needed to get more training.

Anything related to mapping, stratigraphy, taphonomy, and structural geology. Too many paleontologists aren't able to "read rocks". The biological aspects of paleontology only make sense from a good understanding of geology.

Interaction with local communities (in a South Africa-specific context). I haven't received formal training so to speak but I have learned through conversation and observation with and of my PhD advisor respectively.

Other skills that are important: Using a Brunton (or other geologic) compass, general camping/hiking skills, sketching and field note taking. I did have training for these skills in my undergrad field course

Photogrammetry and some fossil preparation itself can be quite helpful in the field too. I had courses on them during both my master´s and PhD, but not as part of the programme itself.

Being able to identify areas where there might be fossils to focus on those has been important for me, rather than just roaming around. No formal training, just experience overtime.

Sedimentary logging, palaeoenvironmental interpretation and field sketches- obtained formal training during undergraduate fieldtrips to Dorset, Pembrokeshire and Germany.

I'm not sure if GIS is included in the above but it is very sought after in collection data and I received no training in it nor opportunities to train.

Not sure this is already accounted for in the previous options, but teamwork in my view is very relevant for a minimally successful fieldwork excursion

Fossil preparation and what to do with samples after collection (ie. will it go into a museum, archive number, etc) No formal training for either.

Ensuring the safety of the crew and the specimen of specimens. Yes, through work with university and museum teams I have acquired this training

Psychology skills and physical fitness are very valuable on the field. I do not have any formal training for both of it, only enthusiast luck.

There never was any formal training offered. I studied biology and always just volunteered for field work and was kind of thrown into it

Field trip logistics (perhaps this is part of field team management), but even just getting a team out in to the field is challenging.

Being familar with operating offroad bakkies, what to do in cases you get stuck in the mud or get a flat tire. I have prior knowledge

In many places, it is important to contact and work with landowners. I have received no formal training in this.

Microfossil site work is important as well as macrofossil. I did formal training with this prior to my PhD

Apart from my master´s program I did not get the change to have any other type of formal training.

Statistical techniques to collect samples, I only had frontal lectures during my master degree

Made some workshops in Poitiers regarding taphonomy, jacketing and excavation methods

Communication and vulgarisation towards the inhabitants of the prospected region. No

would be nice to be trained in how to deal with the public, ie. landowners, etc.

Excavation techniques along with the ample knowledge of Strata are important.

Use of local knowledge, employment of locals in rural areas, and first aid

Field skills in different environments and conditions - limited training

Sample contamination prevention. I received training during my PhD.

To learn the skill of extracting DNA from sub fossilized material

Prospecting. Learned how to do this on a voluntary field season.

I have been able to learn and conduce a animal biology fieldwork

For me I think skill in navigation of unfavorable terrain

Using heavy equipment. No I have not had formal training.

Fossil excavation using power tools including a chainsaw

Structure and geological assemblages of the sediments

Gender perspective, and, no, I have "trained" myself

Off-road driving, snowmobiling, packing and supply

Communication skills with locals and government

Cooking (and I am not being facetious)

Geological sequence interpretation

Logistical planning for fieldwork

Management of volunteers (no)

None that I can think of now

Community life participation

I can't think of anything.

Funding acquisition

Biostratigraphy

First Aid

cooking!

Cooking
