# Supplementary material for: #Yourpalaeolife: Interrogating the Status of Fieldwork Among Early Career Palaeontology Researchers
Source: Ecol Evol. 2026 Jul 29;16(8):e74032. doi: 10.1002/ece3.74032 (PMC13420382; doi:10.1002/ece3.74032)
Supplement: Supplementary file 3 — Data S3: ece374032‐sup‐0003‐Supinfo3.zip. [file ECE3-16-e74032-s001.zip › D7 Open question reporting of unethical practices SI.docx]

No

None

N/A

none

NA

I have encountered a situation wherein two institutions had permits to do palaeontological field work in the same area, where one institution is was working directly in the other's field sites without asking for permission or to collaborate. The supervisor from the offending institution was also using bullying language and behavior when talking to students from the other institution. This situation is in the process of being reported. I have encountered several instances second-had from talks or casual conversations where it appeared that a palaeontologist was conducting fieldwork in a country other than their own and was not respecting, including, or giving back to the local communities and science institutions. This includes traveling to a country for fieldwork and not including locals on the field work team and extracting fossils from a country without promise to return them.

I gave a poster at a professional conference about a field site on private property I had negotiated access and a relationship with a landowner and several amateur collectors who had brought significant material to my attention. Two professionals from a major institution stopped by my poster and openly stated they would just poach the site without coordinating an effort with anyone local including myself. Since then I have seen clear evidence that the site has been hit by extensive excavation which was not coordinated or permission sought with the landowner. It is hard to not make an assumption that these are connected. This has made me uncomfortable presenting my work at professional conferences without already having the paper being presented already submitted and accepted for publication.

In the fieldwork that I have led with other colleagues, we have experienced our permit areas being limited from that of previous years because a museum employee (at the museum that approves the permits) didn't want us to be able to work on any of the areas that they work (even though our fields are only moderately overlapping as they do paleobotanical research and we had no intention of working the same sites as they do). They have also banned researchers from our institution from visiting the paleobotany collections at their museum. They also opened a quarry roughly a meter away from a quarry in which we had been excavating plant fossils, so clearly encroaching on other people's sites is okay for them, but not for anyone else.

Some colleagues found a fossil that was fairly interesting on land that we did not have permits to collect on, but was just over the boundary from land we did have permits to collect on (<10 m), and they decided to collect it anyways and fudge the GPS point of where they had collected it from. This incident has not been reported. I have done geological fieldwork a number of times with non-paleontologists, and there have been times that people have collected vertebrate fossils for personal collections on BLM land without permits. When I first got started, I didn't even know that this was illegal, and only learned later. These incidents have not been reported.

On field work some leaders have a tendency to become "macho" or have "gung-ho" attitudes. This is real problem as it creates a sense of elitism, inconsideration, and puts down others. It also creates an environment where people don't feel welcome to speak up, or trust in the leaders because of over-confidence or "she'll be right" attitude. This is unfortunately quite normal and people will be berated, belittled or not invited back if they do not fit into this regime (and so the cycle continues!). This is seldom reported upwards as the field trip leaders are usually those superiors, but is often talked about among students following field work.

Lack of Emergency Planning: Harsh working conditions with lack of rest. No oversight or emergency plan by the PI when a student tried to change a tire by themself on a very heavy vechicle and it fell and pinned their arm. PI proceeded to bring the person, WHO WAS IN SHOCK, back out to the field on bumpy dirt roads after the hospital visit. This likley prolonged the healing process of the affected arm. Everyone else involved also has a bit of PTSD from the incident. Not reported. Same PI, expeciting grad students to manage local field site that is not part of their dissertations on weekends for no pay.

Yes, I am aware of several cases involving fieldwork conducted in different regions of Africa where local collaborators or support staff were not treated ethically. In these situations, the local personnel were often overworked, underpaid, or not properly acknowledged for their contributions to the research. These practices reflect a lack of respect and fairness toward local communities and their essential role in palaeontological fieldwork. To my knowledge, these incidents were not formally reported to any professional body.

Not personally, only hearing about it from the news (e.g., illegal fossil trade, publishing on illegally-collected material, etc.). Still, I was personally faced ethically dubious opinions from colleagues who did not seem to bother too much about these things and preferred to 'look away' and tried to find reasons (more like excuses) to justify some of this unethical behaviour surrounding palaeontology fieldwork campaigns.

During a palaeontological excavation undertaken outside of the UK, one of the lead researchers made several derogatory comments among his colleagues, which were aimed at a few of the undergraduate students on the dig based on their sexual orientation and ethnicity. Whilst these were not aimed at myself, I felt slightly uncomfortable and offended by this behaviour. The incident was not reported.

It is still a field where the social climate is evolving. During a few trips with some very experienced and acclaimed male researchers in the field I am often expected to take on a more traditionally feminine role. I prepare the food. I manage social blow outs. I "mother" younger students while having to be the respectful "child" student to the older researchers. It's a lot to juggle.

I have seen instances where sites are poorly investigated in terms of context in favour of publishing showy taxon descriptions, leaving these largely in limbo and of limited usefulness. I have also seen the squirreling away of South American fossils by European researchers, without visible attempts to collaborate with the people of the source country. I find these practices dubious.

I was confronted to non-professionnal paleontologists excavating where they should not. They are perfectly aware that what they are doing is illegal and they had some issues in the past but they continue to do so, because they get money out of it (selling to people and even museums, and through exhibition).

Not in the last three years to be honest, but before absolutely yes: Systematically not asking for permits, forcing people to work in extremely dangerous conditions, ignoring good practices and proper velocity of work in order to work faster and recover more elements... etc

I've certainly experienced pressure in field settings to find something big, cool, or which would otherwise be a draw for prestige or for selling public tickets to my museum and institution. The explicit focus on collecting for the sake of trophy hunting is concerning.

Permit areas were greatly reduced from previous years due to overlapping interests, even though no direct incidents of competition for resources occurred. Our site on a limited permit area was then excavated by the palaeontologist who insisted our area be reduced.

I was previously rejected for a Paleontology and Cartography project because I'm a woman; they wanted a man. Since the top three candidates were women, the project was ultimately cancelled. No one was informed of this.

Yes. One of the leaders of the fieldwork told us that a mandatory meeting on non-discrimination among fieldwork participants was necessary. A few people skipped it, downplayed it and went to the fieldwork anyway.

Yes. A tenured faculty member forged permit documents to acquire fossils from another country. I was not part of the project they worked on, but I heard about the unethical behavior from another student.

No, only more broad conversations about whether things are and aren't ethical, how things haven't been ethical in the past and the way our institution and my peers and seniors are working to do better.

I found an archaeological artefact and was encouraged to leave it where I found it, as reporting it would take precedence over the paleontological work and risk having the expedition shut down

Ethics are pretty flexible and a lot of the times the ethics of fieldwork are determined by people in power. I've seen this first hand while attempting to work on Burmese amber.

Previous research teams conducted destructive excavation on a single bed of a section makes it difficult for subsequent researchers to continue collecting fossils.

Again I have been lucky to work with teams that have actually been very ethical in terms of the treatment of specimens both within the U.S and abroad.

None. I am proud to say I've not personally encountered (or at least noticed) unethical fieldwork practices in my whole career.

As said above, researchers which collects data for them and is therefore not or no longer available for research.

Since I haven't been to a lot of paleontological fieldwork, I have no information of something similar happening

Destruction of fossils by PI, fraud, victim blaming, retaliation, breaching and violation of contract conditions

I and many people I know have collected rock or fossil material from national parks or other protected areas.

Several people neglected safety measures. Some people neglected the limitations on fieldwork permits.

Language barrier for international students in countries that doesn't prefer to talk English.

Sometimes, there is no difficulty about ethical dubious at the North West of our country

Private collectors taking and selling fossils that would have been valuable for research

No, but vandalism by non researchers has occured once, and the individual was arrested.

I haven't personally encountered any delicate situations in the last three years.

Not involving or trying to engage with Traditional owners.

Yes, excavation in restriced area, keeping of fossis

Not personally, just heard about things.

Yes, but not from my organization

Not in the last 3 years, no.

Not in the last 3 years.

In the field, not yet.

Not to my knowledge.

No such encounters

Not that I recall

None encountered

I have not

Yes
