# Supplementary material for: #Yourpalaeolife: Interrogating the Status of Fieldwork Among Early Career Palaeontology Researchers
Source: Ecol Evol. 2026 Jul 29;16(8):e74032. doi: 10.1002/ece3.74032 (PMC13420382; doi:10.1002/ece3.74032)
Supplement: Supplementary file 3 — Data S3: ece374032‐sup‐0003‐Supinfo3.zip. [file ECE3-16-e74032-s001.zip › D8 Open question why not organise fieldwork SI.docx]

Fieldwork is not necessary for my research, I have always considered it something it would be beneficial to gain some experience of but I have not prioritised it. I do not have the skills or knowledge to organise my own trip, which would make me reliant on joining a trip organised by someone else - as a woman with existing health issues and a propensity to become exhausted by too much social contact, I do not trust others to organise a trip on which I would feel safe and comfortable. The last time I attended a teaching field trip, I asked to know when we would have access to toilets, and this was forgotten about - as we were on beaches, it was difficult to even find a sheltered/secluded spot in which to pee. As such, I would rather concentrate on my work (and doing what I do best) than subject myself to an ordeal in order to gain field experience.

I was only able to apply for the field work that I participated in because my advisor had experience doing so. If I didn't work with him, the lack of knowledge about how to apply for the field work project would have been prohibitive. I think this is a common problem where people want to apply for projects, but don't have the specific mentoring necessary to make a successful application. It may not have been a barrier to my participation, but I see it as a barrier for others, especially from countries outside the US and Europe, and people from marginalized communities or smaller geoscience programs.

As mentioned above Covid prevented travel in China, so I will stick to the two years since I returned to the US. My main concern has been instability. Time spent in the field is time not spent working a non-Paleo job to keep my lights on or not spent applying to Paleo jobs or not spent working on papers that can appeal to funders to get a postdoc. Field work is expensive and funding to live takes precedence.

I was advised against primary field collection by my supervisory team, although I was very interested at earlier stages in collecting and documenting from some areas myself. I still am interested in documenting species and stratigraphy at one UK site, but I will likely do this in my own time if at all.

After 2 successful field seasons, there was no need for additional fieldwork, and I needed to focus my time on analyses. My lab mate does not need fieldwork, so it's harder to join others nearby.

It was a very busy time for me personally, finishing up my PhD without funding. Furthermore, due to the pandemic, my PhD was reorganised completely so I did not need fieldwork.

I would have loved to do field work but it is outside of the scope of my PhD so I ultimately had to choose between spending my holiday visiting my family or in the field

My PhD research is on geochemical analysis of fossils, which means I go to collections for sampling and do no (sadly) get to participate in excavations/fieldwork myself.

I have not engaged in fieldwork in the last three years because of financial concerns, not needing it for current projects, and concerns about my health/disability.

I've been focused on my PhD degree for the past years and honestly couldn't find the time to think of extra academic activities besides my research

My project is in digital model and doesn’t require fieldwork. As I don’t come from a paleo background I don’t have much interest in fieldwork.

My current supervisors are uncomfortable with paleontological fieldwork, and such fieldwork is not explicitly required by my current project

In my PhD, I do micropaleontology and my samples comes from deep sea drilling projects. So I don't have to participate in fieldwork for now.

I was lucky to be included into already-organised fieldworks, but I did not organised any as my PhD subject relied on collection material.

Areas of interest have been claimed by other Palaeontologists. And the administrative work of getting a collection permit

It is not relevant to my research, and I believe it to be too expensive to participate in purely for skills development.

I haven't come across any independent courses, only within conferences and I did not have the funds to attend them

I've been busy finishing the text and analysis in my thesis, and then looking for post-doctorate grants.

I am still early in the career and felt it would be out of my league to organise fieldwork of my own.

Lack of awareness of opportunities coupled with a general lack of relevance to my scientific focus

Not relevant to my postdoctoral work and I have plenty of experience from my PhD

I have a bit, but my lab doesn’t really do fieldwork often.

No opportunities nearby / difficulty finding contacts

Not enough time during PhD and not enough money, too

Lack of funding by my institution and research group

Field sites are permanently closed (coal seams)

I have no more interest in doing fieldwork

Lack of time, focusing on my PhD research

I usually just buy fossils for research

I was still getting the practice

I'm not interested in fieldwork

Not necessary for my project

Not enough knowledge

No need to do so
